# Efficacy and Benefit of Postoperative Chemotherapy in Micropapillray or Solid Predominant Pattern in Stage IB Lung Adenocarcinoma: A Systematic Review and Meta-Analysis

**DOI:** 10.3389/fsurg.2021.795921

**Published:** 2021-12-21

**Authors:** Congcong Xu, Kanghao Zhu, Dong Chen, Yuhang Ruan, Zixian Jin, Hongbin Qiu, Baofu Chen, Jianfei Shen

**Affiliations:** ^1^Key Laboratory of Minimally Invasive Techniques & Rapid Rehabilitation of Digestive System Tumor of Zhejiang Province, Department of Cardiothoracic Surgery, Taizhou Hospital of Zhejiang Province Affiliated to Wenzhou Medical University, Linhai, China; ^2^Key Laboratory of Minimally Invasive Techniques & Rapid Rehabilitation of Digestive System Tumor of Zhejiang Province, Department of Cardiothoracic Surgery, Taizhou Hospital, Zhejiang University, Linhai, China

**Keywords:** postoperative chemotherapy (POCT), overall survival (OS), disease-free survival (DFS), micropapillary or solid pattern, lung adenocarcinoma

## Abstract

**Background:** The benefit of postoperative chemotherapy remains controversial for patients with either a micropapillary or solid pattern in stage IB non-small cell lung cancer. This study is designed to explore the significance of postoperative chemotherapy in patients with either a micropapillary or solid pattern in stage IB lung adenocarcinoma.

**Method:** To conduct the meta-analysis, PubMed, Cochrane Library, Embase and Medline were used to collect literature on long-term follow-up studies published before March, 2021, involving postoperative chemotherapy for patients with both a micropapillary or solid pattern in stage IB lung adenocarcinoma as compared to non-postoperative chemotherapy. Survival data was extracted from the literature, including the overall survival and disease-free survival. Based on overall survival and disease-free survival, hazard ratios and their 95% of confidence intervals were applied to assess the prognostic effect of postoperative chemotherapy. Review Manager software was used to merge the effect size for the meta-analysis.

**Result:** In total, 6 papers with 956 patients were included. In terms of the prognosis of patients suffering from lung cancer when receiving postoperative chemotherapy, this study comprehensively reviews and evaluates the available evidence of micropapillary or solid patterns. After excluding the heterogeneity between the studies, we found that the pooled results from 6 studies report that postoperative chemotherapy was associated with a better overall survival rate when compared with non-postoperative chemotherapy (hazard ratio = 0.58, 95% confidence interval, 0.44–0.77; *P* = 0.0002). Postoperative chemotherapy also significantly improved the disease-free survival in patients with either a micropapillary or a solid pattern in stage IB lung adenocarcinoma (postoperative chemotherapy vs. non-postoperative chemotherapy, hazard ratio = 0.51, 95% confidence interval, 0.40–0.64; *P* < 0.001). However, a subgroup analysis showed that compared with non-postoperative chemotherapy, tumor size was unrelated to the prognosis of patients in stage IB undergoing postoperative chemotherapy (hazard ratio = 0.98, 95% confidence interval, 0.94–1.02; *P* = 0.27).

**Conclusion:** Postoperative chemotherapy results in a better long-term survival rate for patients with either a solid or a micropapillary pattern in stage IB lung adenocarcinoma. Multi-center, prospective, clinical trials are needed to validate our findings.

## Introduction

Non-small cell lung cancer (NSCLC) remains the most common cause of malignant tumors and cancer-related deaths in the world, of which adenocarcinoma is the most common histological subtype (1). At present, surgical resection is the primary treatment for early-stage NSCLC (2). Additional treatment for NSCLC includes chemotherapy, radiotherapy, immunotherapy and the other treatments. Postoperative chemotherapy is defined as the application of chemical drugs to tumor patients after surgery to shrink the primary tumor, and at the same time may eliminate the remaining micrometastasis, reduce the chance of tumor recurrence and metastasis, and improve the cure rate. According to NCCN clinical practice guidelines, postoperative chemotherapy for patients with IB stage non-small cell lung cancer is recommended from time to time occasionally (3). However, it has been demonstrated that chemotherapy may bring survival benefit for micropapollary predominant subtype patients in stage IB (4). Several meta-analyses and randomized controlled trials have shown that postoperative chemotherapy (POCT) substantially improves survival in patients with resected stage II and IIIA NSCLC (5–10); however, it is unclear and inconclusive whether POCT can improve the overall survival (OS) and disease-free survival (DFS) of patients with resected stage IB NSCLC remains ambiguous and inconclusive. Further studies are necessary to determine its efficacy in patients with stage I NSCLC. Adjuvant Navelbine International Trialist Association test stratified analysis showed that postoperative chemotherapy did not significantly improve the survival rate of patients with stage IB (7). A lack of convincing evidence results in it still unclear whether these patterns in stage IB patients receive any survival benefits from POCT.

According to the predominant pattern, invasive lung adenocarcinoma is categorized into several subtypes, including lepidic, acinar, papillary, solid, and micropapillary pattern lung adenocarcinoma. Micropapillary or solid subtype lung cancer is a poorly differentiated lung adenocarcinoma with a poor prognosis. Several studies have shown that postoperative chemotherapy can improve the prognosis of patients with either a micropapillary or solid pattern in stage IB lung adenocarcinoma (11, 12), while some studies have reported that postoperative chemotherapy is unrelated to the prognosis (13). Therefore, this study aims to determine whether patients with either micropapillary or solid pattern in stage IB lung adenocarcinoma, based on the 8th TNM classification, could benefit from POCT. Given the development of computed tomography scan and the attention to health, the discovery rate of early-stage lung adenocarcinomas is rapidly increasing and we wish that our findings will have a beneficial impact on the management of stage IB patients with either a micropapillary or solid pattern lung adenocarcinoma, which could translate into improving the outcomes of stage IB lung adenocarcinomas patients with micropapillary or solid pattern.

## Materials and Methods

### Search Strategy

**Figure 1:**
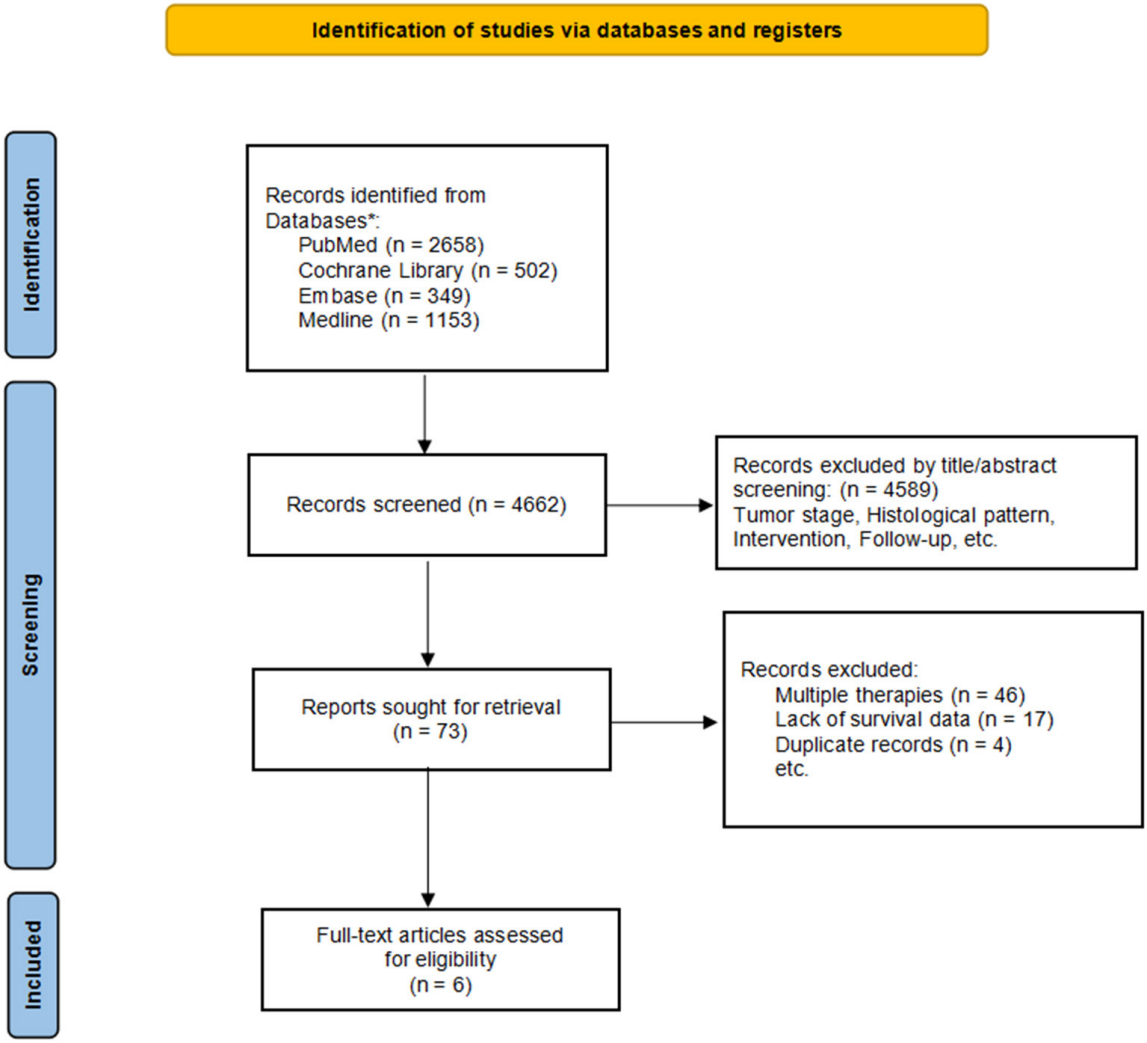
Flow chart of literature screening.

This study used the following keywords to search for literature in PubMed, Embase, the Cochrane library, and Medline. We used medical keywords to search for lung adenocarcinoma (including lung neoplasm and lung cancer), chemotherapy (including postoperative chemotherapy, and adjuvant chemotherapy) and early stage. The search was limited to publications published in English. Our search process was based on systematic reviews and meta-analysis guidelines. The specific search process is shown in Figure 1.

### Selection Criteria

Eligible researches that meet the criteria are selected according to the following criteria: (1) All patients were diagnosed as stage IB NSCLC; (2) The selected patients were diagnosed as micropapillary or solid pattern lung adenocarcinoma; (3) There was a comparison of POCT and no-POCT for lung adenocarcinoma; (4) There were survival data for long-term follow-up; and (5). Each treatment cohort must have >10 people. All studies were published in the English language. Duplicated publications were excluded, as were publications which do not contain the original data, such as review articles, case reports, and letters. Two researchers independently reviewed the titles and abstracts of the retrieved articles using the above selection criteria. If it is obviously unqualified, the article will be rejected. If the abstract could not be determined, the full text was further evaluated. Any differences are resolved through discussion with the third author.

### Statistical Analysis

All data from each eligible study were extracted, including the hazard ratio (HR) and 95% confidence interval (CI) in order to analyze the overall survival (OS) and disease-free survival (DFS) benefits of the treatment methods. The HR and 95% CI values were best obtained directly from the literature, but if this was not possible, we used the method described by Parmer et al. to estimate the values based on other relevant data in the article (14). After obtaining the HR and 95% CI interval values, we convert them to logHR and SE, done automatically using the Review manager software. Before conducting a meta-analysis, we first assessed the heterogeneity between studies using *I*^2^ to quantify the degree of heterogeneity as follows: 0–40% was low heterogeneity; 40–70% was moderate heterogeneity; and >70% was high heterogeneity. According to the heterogeneity, we chose the random effect model or fixed effect model to combine the effect size. We chose the fixed effect model in low heterogeneity while random effect model in high heterogeneity. For high heterogeneity, we performed meta-regression to identify potential sources of bias. Two-sided *p* ≤ 0.05 was considered statistically significant. All the analyses were performed in Revman 5.30 version.

## Results

### Literature Results

A total of 4,662 records were retrieved from PubMed, EMBASE, Cochrane library, and Medline, in which 4,589 articles were considered ineligible on the basis of title and abstract and 73 were potentially relevant. Upon further review, 67 articles were excluded: 46 papers presented mixed cohorts of patients undergoing radiotherapy, chemotherapy, and targeted therapy, 17 did not clearly describe the survival data related to this study, and 4 were removed due to duplicates. Finally, 6 studies were selected for the analysis and the main characteristics are summarized in Table 1.

**Table 1:** Baseline characteristics of the included study.

**Researches**	**Country**	**No. All**		**No. Micropapillary**		**Outcomes**	**Follow-up**	**Sex**		**Age**	**Type of**
				**or solid pattern**			**(months)**				**surgery**
		**Surgery alone**	**POCT**	**Surgery alone**	**POCT**			**Male**	**Female**		
Luo et al. (11)	China	419	509	37	51	OS/DFS	46.53 (0–83)	457	471	<55 years 230 55–70 years 546 >70 years 152	Lobectomy 816 Wedge resection 94 Others 18
Qian et al. (12)	China	510	621	51	89	OS/DFS	46.8	544	587	NR	Lobectomy 1018 Segmentectomy 113
Tsao et al. (13)	Toronto	293	282	164	141	OS/DFS	67.2	365	210	<55 years 200 55–64 years 216 ≥65 years 159	NR
Ma et al. (15)	China	348	149	28	23	DFS	38.6 (1.7–96.6)	209	288	<65 years 355 ≥65 years 142	Lobectomy 475 Segmentectomy 22
Hung et al. (16)	China	173	70	37	26	DFS	45.1 (4.8–110.0)	126	117	NR	NR
Cao et al. (17)	China	116	193	116	193	OS/DFS	41 (7–98)	119	110	<60 years 158 ≥60 years 151	Lobectomy 305 Segmentectomy 4

*OS, overall survival; DFS, disease-free survival; NR, not report*.

### Overall Survival Rate

**Figure 2:**
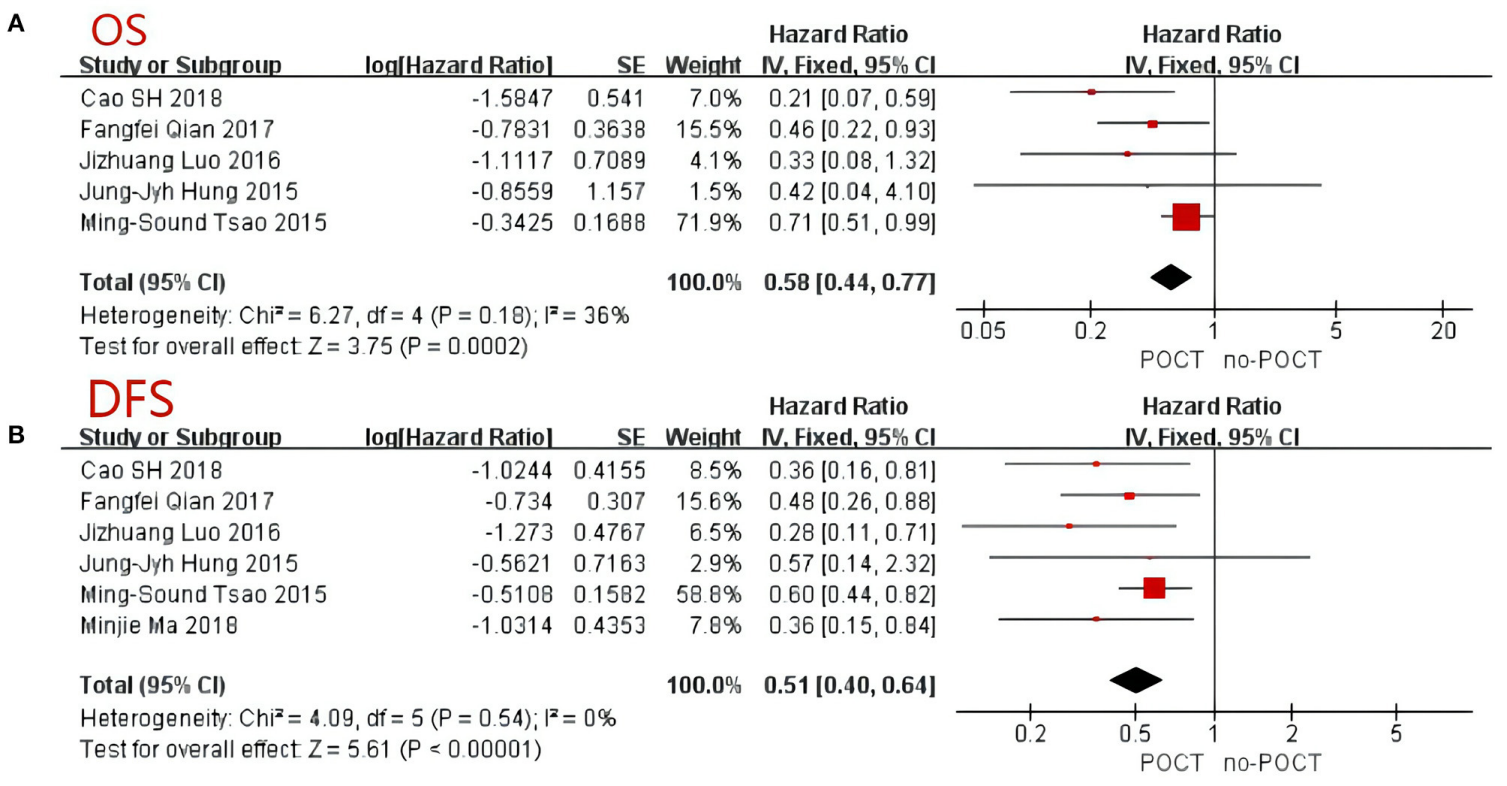
Forest Plot for overall survival **(A)** and disease-free survival **(B)** of patients with micropapillary or solid pattern in stage I lung adenocarcinoma. CI, confidence interval; HR, hazard ratio; SE, standard error. HR < 1 meant a better prognosis in the postoperative chemotherapy group. I^2^ < 40% can choose a fixed-effects model, otherwise choose a random-effects model.

A total of 956 patients were pooled from the six studies (11–13, 15–17), including micropapillary predominant pattern (*n* = 89, 9.3%), solid predominant pattern (*n* = 676, 70.7%), and micropapillary or solid predominant pattern (*n* = 191, 20.0%). According to the data extraction results, one article failed to extract the corresponding overall survival rate. As such, we included five articles to analyze the overall survival rate of patients (11–13, 16, 17). The result of the heterogeneity analysis showed that *I*^2^ = 36%, low heterogeneity, and we used the fixed effects model. The results showed the total hazard ratio was 0.58 (95% CI, 0.44–0.77; Figure 2A). Postoperative chemotherapy significantly improved the OS of patients with micropapillary or solid pattern in stage IB lung adenocarcinoma compared to those without postoperative chemotherapy (*p* = 0.0002).

### Disease-Free Survival Rate

All six trials provided data on 5-year disease-free survival (DFS) (11–13, 15–17). The comprehensive analysis showed that survival benefit was achieved with postoperative chemotherapy (*p* < 0.001; Figure 2B). Because of the low heterogeneity between trials (*I*^2^ = 0%), a fixed-effects model was adopted and a comprehensive HR of 0.51 was achieved (95% CI, 0.40–0.64). Similar to the OS, postoperative chemotherapy significantly improved the DFS of patients with either a micropapillary or solid pattern in stage IB lung adenocarcinoma.

### The Subgroup Analysis of Tumor Size in Micropapillary or Solid Pattern Stage IB Patients

**Figure 3:**
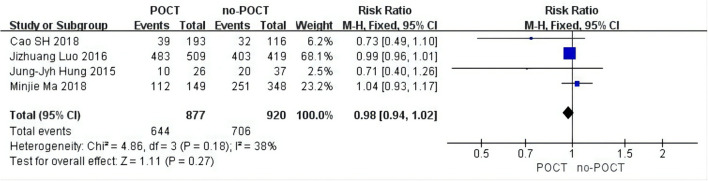
Forest plots of tumor size in micropapillary or solid predominant pattern patients. CI, confidence interval; HR, hazard ratio; SE, standard error. RR < 1 meant a better prognosis in the postoperative chemotherapy group. I^2^ < 40% can choose a fixed-effects model, otherwise choose a random-effects model.

The tumor size subgroup analysis included four data inputs (11, 12, 15, 17). Because there was low heterogeneity (*I*^2^ = 22%), a fixed-effects model was adopted and we found that compared with no-POCT, tumor size was unrelated to the prognosis of patients in stage IB undergoing postoperative chemotherapy (HR = 0.85; 95% CI, 0.64–1.13; *p* = 0.27; Figure 3).

### Sensitivity Analysis and Publication Bias

The number of studies included in the meta-analysis is relatively small, <10. The asymmetry of the funnel diagram is too low to distinguish whether it is real asymmetry or not. We use the RevMan5.3 version to evaluate a systematic review of qualified research quality based on Cochrane Handbook for Systematic Reviews of Interventions and then summarized the bias risk of each study in Table 2.

**Table 2:** Risk of Bias of included studies.

	**Random sequence generation**	**Allocation concealment**	**Blinding of participants and personnel**	**Incomplete outcome data**	**Selective reporting**	**Other bias**
Luo et al. (11)	+	*	+	+	+	–
Qian et al. (12)	+	+	+	+	+	–
Tsao et al. (13)	+	*	+	+	+	*
Ma (2015)	+	*	+	+	–	–
Hung et al. (16)	+	*	+	+	–	–
Cao et al. (17)	+	+	+	+	+	–

+, Low risk of bias; ^*^, Unclear risk of bias; –, High risk of bias.

## Discussion

The new grading system for invasive pulmonary adenocarcinoma and the 2015 World Health Organization classification of pulmonary adenocarcinoma (based on the mainly histological pattern) have been found to be associated with prognosis, and adenocarcinomas are divided into three subtypes: low-grade (mainly lepidic), intermediate-grade (mainly acinar or papillary) and high-grade (mainly solid or micropapillary) (18, 19). And the dominant subtype is determined according to the pattern with the highest proportion in histology (18). In clinical practice, we can evaluate the condition, prognosis and subsequent treatment strategies of patients in stage IB lung adenocarcinoma who underwent surgical resection according to the new grading system model. In general, a histological pattern is a strong prognostic indicate for invasive pulmonary adenocarcinoma. The prognosis of patients with a histological low-grade malignant lung cancer is better than that of a histological high-grade malignant lung cancer. Micropapillary-predominant and solid-predominant subtypes were important prognostic factors for recurrence and death. Necessarily, further research will be conducted on the prognosis and subsequent treatment of patients with either a micropapillary or solid pattern lung adenocarcinoma. As an important adjuvant therapy, the role of POCT in NSCLC is an important issue concerned by oncologists. In recent years, several studies have explored the benefits of postoperative chemotherapy for NSCLC (8, 10, 20–24). However, few studies has been done to explore the survival benefits of POCT in patients with early lung cancer, especially in micropapillary or solid lung adenocarcinoma. Therefore, the purpose of this meta-analysis was to explore the effect of POCT on the prognosis of micropapillary or solid predominant pattern lung adenocarcinoma.

It is reported that micropapillary or solid pattern are the most invasive pattern associated with lymphatic invasion, pleural infiltration and lymph node metastasis (25). Micropapillary or solid pattern were prognostic predictors for decreased disease-free survival of early-stage lung adenocarcinoma patients (26). In addition, micropapillary or solid pattern patients may benefit more from POCT than other histological subtypes, especially for patients with stage II or higher (26). With the popularity of lung cancer screening, more and more early lung cancers are found and treated by surgical resection. The postoperative pathology of a considerable number of people suggests micropapillary or solid predominant pattern lung adenocarcinoma. Therefore, it is extremely important to decide the subsequent treatment strategy based on histology. However, few studies have shown that POCT contributed to survival benefits in the micropapillary or solid subgroup of patients with stage IB lung adenocarcinoma (11, 12, 15). In this meta-analysis, a total of 956 patients with stage IB micropapillary or solid predominant pattern lung adenocarcinoma were collected from 6 studies. We found that POCT was associated with a better OS when compared with no-POCT and the effect was significant (HR 0.58; 95%CI, 0.44–0.77; Figure 2A). POCT also significantly improved the DFS in patients with micropapillary or solid pattern in stage IB lung adenocarcinoma (HR 0.51; 95% CI, 0.40–0.64; Figure 2B). In multivariable analyses, compared with other predominant histological patterns, micropapillary or solid pattern predominant lung adenocarcinoma benefited from POCT in both OS and DFS. Although there are two articles shows POCT could not improve the OS of these patients (11, 16), the pooled analysis outcome showed the benefit of OS was related to POCT. In our analysis, we found that when the nodule is essentially micropapillary or solid predominant pattern, the survival outcome of POCT, including OS and DFS, was significantly better than that of no-POCT. Other meta-analyses yielded similar results. Hamada et al. focused on POCT with UFT in stage IB NSCLC, and reported that the 5-year OS rate in the POCT group was significantly higher than that in the control group (81.5% vs. 77.2%, *P* = 0.011, HR: 0.74) (21). These findings provide evidence for POCT in stage IB micropapillary or solid predominant subtype patients.

Bria et al. showed that patients with stage I–III disease who received cisplatin-based chemotherapy had a longer survival than those who underwent surgery alone, including early NSCLC (12). However, the accuracy of survival determination may be affected by relative risks and incomplete paper trials in this analysis. This result was consistent with the Lung Adjuvant Cisplatin Evaluation Collaborative Group, which conducted a cisplatin-based chemotherapy trial on patients with stage I–III disease (8). Based on the fact there is no uniform standard, we conducted this meta-analysis. After summarizing the data, we found survival advantages to receiving postoperative chemotherapy in early NSCLC patients. The OS (HR 0.58; 95% CI, 0.44–0.77) and DFS (HR 0.51; 95% CI, 0.40–0.64) of patients with either a micropapillary or solid pattern lung adenocarcinoma received postoperative chemotherapy better than those without chemotherapy. Finally, these results still need to be confirmed in large-scale randomized controlled trials in the future.

### Limits

The study has several limits. First, this study was a meta-analysis and we did not have access to the detailed data from individual patients. In addition, it is not clear whether these patients were under the care of the same surgeon and unable to obtain their chemotherapy treatments. Also, it is not clear whether the patients were under the care of the same surgeon and their chemotherapy treatment methods could not be obtained. Third, the inclusion and exclusion criteria of the retrospective studies we included were different. Finally, for some studies, we were unable to extract the survival data and were unable to contact the authors. With these factors, the conclusions of this study require confirmation with multi-center, prospective clinical trials to improve our understanding of the benefits of postoperative chemotherapy for micropapillary or solid lung adenocarcinoma so as to improve the prognosis of patients.

## Conclusion

Postoperative chemotherapy could improve survival rate and disease-free survival of patients with either a micropapillary or solid pattern in stage IB lung adenocarcinoma. In the future, multi-center, prospective, clinical trials are needed to validate these findings.

## Data Availability Statement

The original contributions presented in the study are included in the article/Supplementary Material, further inquiries can be directed to the corresponding author/s.

## Author Contributions

CX, KZ, and DC: conceptualization, methodology, and resources. BC and JS: supervision and funding acquisition. All authors: formal analysis and investigation, writing-original draft preparation, and writing-review and editing.

## Conflict of Interest

The authors declare that the research was conducted in the absence of any commercial or financial relationships that could be construed as a potential conflict of interest.

## Publisher's Note

All claims expressed in this article are solely those of the authors and do not necessarily represent those of their affiliated organizations, or those of the publisher, the editors and the reviewers. Any product that may be evaluated in this article, or claim that may be made by its manufacturer, is not guaranteed or endorsed by the publisher.

## Abbreviations

POCT: postoperative chemotherapy
no-POCT: non-postoperative chemotherapy
NSCLC: non-small cell lung cancer
OS: overall survival
DFS: disease-free survival
HR: hazard ratio
CI: confidence interval.
