# Supplementary material for: Efficacy and Benefit of Postoperative Chemotherapy in Micropapillray or Solid Predominant Pattern in Stage IB Lung Adenocarcinoma: A Systematic Review and Meta-Analysis
Source: Front Surg. 2021 Dec 21;8:795921. doi: 10.3389/fsurg.2021.795921 (PMC8724201; doi:10.3389/fsurg.2021.795921)
Supplement: Supplementary file 1 [file Data_Sheet_1.ZIP › ╬─╧╫/106.pdf]

Published Ahead of Print on April 27, 2015 as 10.1200/JCO.2014.58.8335
The latest version is at http://jco.ascopubs.org/cgi/doi/10.1200/JCO.2014.58.8335

JOURNAL OF CLINICAL ONCOLOGY — ORIGINAL REPORT

Subtype Classification of Lung Adenocarcinoma Predicts Benefit From Adjuvant Chemotherapy in Patients Undergoing Complete Resection

Ming-Sound Tsao, Sophie Marguet, Gwénaël Le Teuff, Sylvie Lantuejoul, Frances A. Shepherd, Lesley Seymour, Robert Kratzke, Stephen L. Graziano, Helmut H. Popper, Rafael Rosell, Jean-Yves Douillard, Thierry Le-Chevalier, Jean-Pierre Pignon, Jean-Charles Soria, and Elisabeth M. Brambilla

Ming-Sound Tsao and Frances A. Shepherd, Princess Margaret Cancer Centre, University Health Network, and University of Toronto, Toronto; Lesley Seymour, NCIC Clinical Trials Group and Queen's University, Kingston, Ontario, Canada; Sophie Marguet, Gwénaël Le Teuff, Thierry Le-Chevalier, Jean-Pierre Pignon, and Jean-Charles Soria, Gustave Roussy, Villejuif; Sylvie Lantuejoul and Elisabeth M. Brambilla, Inserm U823, Centre Hospitalier Universitaire de Grenoble, Institut Albert Bonniot, Université Joseph Fourier, Grenoble; Jean-Yves Douillard, Centre René Gauducheau, Saint-Herblain, Nantes, France; Robert Kratzke, University of Minnesota, Minneapolis, MN; Stephen L. Graziano, SUNY Upstate Medical University, Syracuse, NY; Helmut H. Popper, Institute of Pathology, University Medical School of Graz, Graz, Austria; and Rafael Rosell, Catalan Institute of Oncology, Barcelona, Spain.

Published online ahead of print at www.jco.org on April 27, 2015.

Written on behalf of the LACE-Bio Collaborative Group.

Support information appears at the end of this article.

M.-S.T. and E.M.B. contributed equally to this work.

Authors' disclosures of potential conflicts of interest are found in the article online at www.jco.org. Author contributions are found at the end of this article.

Corresponding author: Ming-Sound Tsao, MD, Department of Pathology, University Health Network, 200 Elizabeth St, Toronto, Ontario M5G 2C4 Canada; e-mail: ming.tsao@uhn.ca.

© 2015 by American Society of Clinical Oncology

0732-183X/15/3399-1/$20.00

DOI: 10.1200/JCO.2014.58.8335

A B S T R A C T

Purpose
The classification for invasive lung adenocarcinoma by the International Association for the Study of Lung Cancer, American Thoracic Society, European Respiratory Society, and WHO is based on the predominant histologic pattern—lepidic (LEP), papillary (PAP), acinar (ACN), micropapillary (MIP), or solid (SOL)—present in the tumor. This classification has not been tested in multi-institutional cohorts or clinical trials or tested for its predictive value regarding survival from adjuvant chemotherapy (ACT).

Patients and Methods
Of 1,766 patients in the IALT, JBR.10, CALGB 9633 (Alliance), and ANITA ACT trials included in the LACE-Bio study, 725 had adenocarcinoma. Histologies were reclassified according to the new classification and then collapsed into three groups (LEP, ACN/PAP, and MIP/SOL). Primary end point was overall survival (OS); secondary end points were disease-free survival (DFS) and specific DFS (SDFS). Hazard ratios (HRs) and 95% CIs were estimated through multivariable Cox models stratified by trial. Prognostic value was estimated in the observation arm and predictive value by a treatment effect interaction with histologic subgroups. Significance level was set at .01 for pooled analysis.

Results
A total of 575 patients were included in this analysis. OS was not prognostically different between histologic subgroups, but univariable DFS and SDFS were worse for MIP/SOL compared with LEP or ACN/PAP subgroup ($P < .01$); this remained marginally significant after adjustment. MIP/SOL patients (but not ACN/PAP) derived DFS and SDFS but not OS benefit from ACT (OS: HR, 0.71; 95% CI, 0.51 to 0.99; interaction $P = .18$; DFS: HR, 0.60; 95% CI, 0.44 to 0.82; interaction $P = < .01$; and SDFS: HR, 0.59; 95% CI, 0.42 to 0.81; interaction $P = .01$).

Conclusion
The new lung adenocarcinoma classification based on predominant histologic pattern was not predictive for ACT benefit for OS, but it seems predictive for disease-specific outcomes.

J Clin Oncol 33. © 2015 by American Society of Clinical Oncology

INTRODUCTION

More than 80% to 90% of lung adenocarcinomas demonstrate heterogeneous histologic patterns and are classified as mixed type according to the WHO classification (third edition).[1] In 2011, an international multidisciplinary expert panel endorsed by the International Association for the Study of Lung Cancer (IASLC), American Thoracic Society (ATS), and European Respiratory Society (ERS) recommended a new classification system that recognized subtyping of invasive adenocarcinoma based on the predominant histologic pattern present in the resected tumor.[2] This classification forms the basis of the fourth edition of the WHO classification to be published in 2015.[3] This classification system has been validated for its reproducibility.[4] More importantly, studies across four continents (North America, Europe, Australia, and Asia) have identified different prognostic groups among these subtypes of resected lung adenocarcinoma.[5-8] In particular, invasive adenocarcinomas with micropapillary (MIP) and predominantly solid patterns (SOL) consistently have been associated with poorer prognosis (Data Supplement). However, this classification system has not been tested in clinical trials. Furthermore, its utility in predicting survival benefit from adjuvant chemotherapy (ACT) has not been reported.

The Lung Adjuvant Cisplatin Evaluation Biomarker (LACE-Bio) collaborative group was

Information downloaded from jco.ascopubs.org and provided by at Gazi Universitesi on May 1, 2015 from 194.27.18.18
Copyright © 2015 American Society of Clinical Oncology. All rights reserved.
Copyright 2015 by American Society of Clinical Oncology

formed to conduct validation studies or pooled analyses of promising biomarkers in a large cohort of patients participating in four ACT trials: the IALT (International Adjuvant Lung Cancer Trial),[9] ANITA (Adjuvant Navelbine International Trialist Association),[10] JBR.10,[11] and CALGB (Cancer and Leukemia Group B; now Alliance for Clinical Trials in Oncology) 9633 studies.[12] Representative hematoxylin and eosin (HE)– stained slides from a large subset of these patients have been collected, and they provide a unique opportunity to assess the prognostic and predictive value of the new classification system. The results may provide strong rationale for its adoption in routine clinical practice and as a potential stratification factor in future clinical trials.

PATIENTS AND METHODS

Patients and Pathology Materials

Of 1,766 patients with non–small-cell lung carcinoma in the LACE-Bio cohort, 725 had an original diagnosis of adenocarcinoma. For 645 patients, one representative HE-stained slide of the surgically resected tumor was available for pathologic review, leading to 629 with nonmissing and 16 with missing subtypes (Data Supplement). The sections were scanned by the Aperio ScanScope XT whole-slide scanning system (Leica Microsystems, Concord, Ontario, Canada) at 20× objective magnification. Using ImageScope software (Leica Microsystems), the images were reviewed independently by coauthors of the IASLC/ATS/ERS lung adenocarcinoma classification system (M.-S.T., E.M.B.).[2,13] Discrepant diagnoses were resolved by consensus. Invasive adenocarcinomas with mixed histologic patterns were classified into one of following subtypes based on the predominant growth pattern present in the tumor: lepidic (LEP), acinar (ACN), papillary (PAP), MIP, and SOL (Fig 1). For patient cases with neuroendocrine histologic features and a block available for further immunohistochemistry study, synaptophysin was performed to confirm the diagnosis of large-cell neuroendocrine carcinoma. The invasive mucinous adenocarcinoma subtype and other variants described in the IASLC/ATS/ERS classification were excluded from the statistical analyses to focus on these five subtypes.

Statistical Methods

Median follow-up was estimated by the reverse Kaplan-Meier method.[14] The main end point of this analysis was overall survival (OS), defined as time

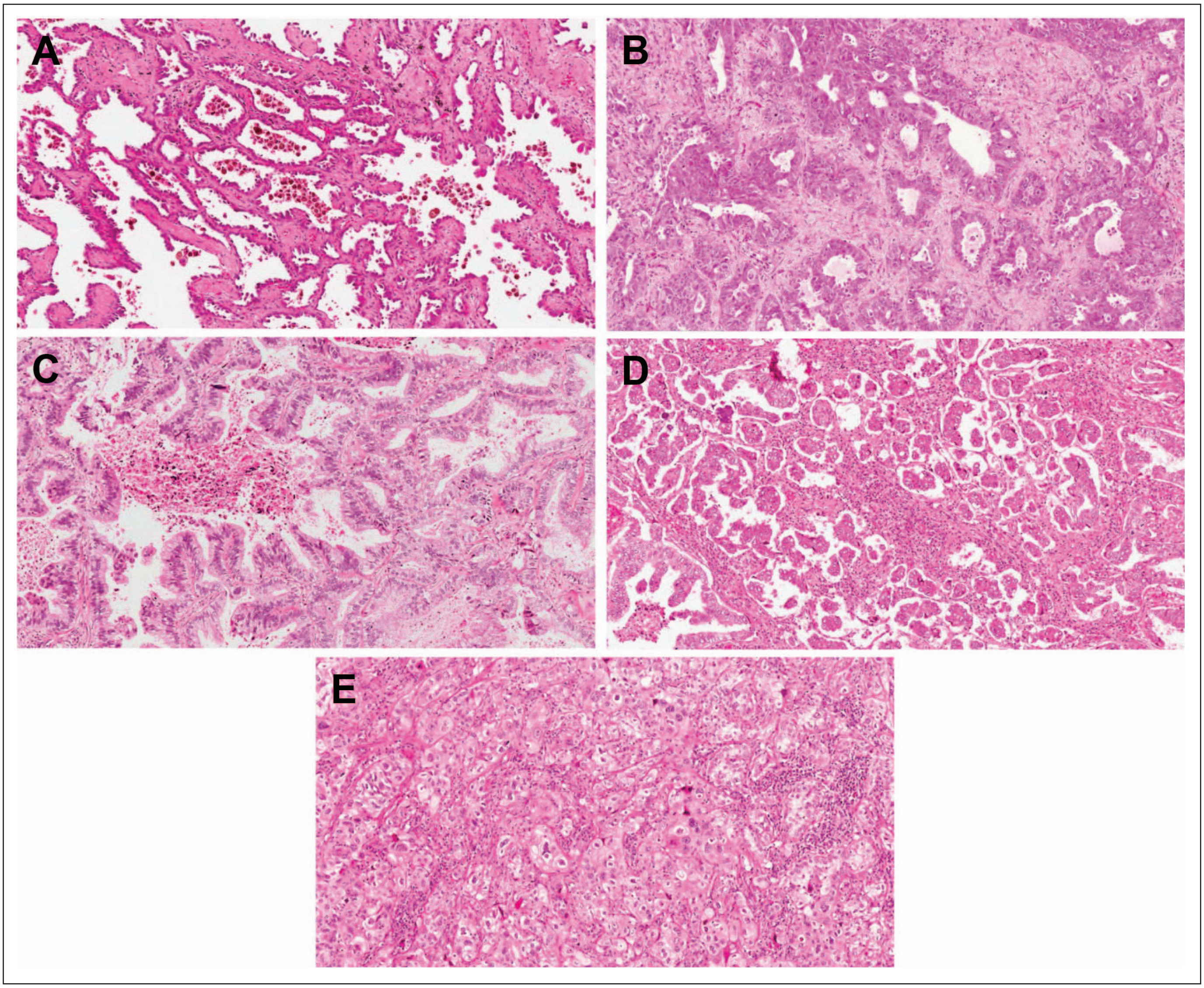

Fig 1. Representative histologic images of five adenocarcinoma patterns: (A) lepidic, (B) acinar, (C) papillary, (D) micropapillary, and (E) solid.

Information downloaded from jco.ascopubs.org and provided by at Gazi Universitesi on May 1, 2015 from 194.27.18.18
Copyright © 2015 American Society of Clinical Oncology. All rights reserved.

from random assignment to date of death resulting from any cause. Secondary end points included disease-free survival (DFS), defined as time from random assignment to date of first event (recurrence or death), and specific DFS (SDFS), defined as time from random assignment to cancer-related event (ie, noncancer deaths were censored at date of death [eg, death resulting from toxicity]). Patients with no event were censored at the date of their last follow-up. The five histologic subtypes (LEP, ACN, PAP, MIP, and SOL) were collapsed into three groups (LEP, ACN/PAP, and MIP/SOL) because the number of patients in some groups was too small for inferential analyses. The correlation between the histologic subtypes and covariates was tested using the Cochran-Mantel-Haenszel test. The log-rank test was used to compare the survival curves estimated by the Kaplan-Meier method between histologic subtypes. Multivariable Cox models stratified by trial and adjusted for sex, age, nodal status (N0, N1, or N2), tumor size (T1, T2, or T3 to T4), type of surgery, and WHO performance status (main analyses) were used to measure the prognostic value of these subtypes in the observation arm. To evaluate whether specific histologic subtypes could predict survival benefit from chemotherapy, an interaction term between subtype and treatment (chemotherapy *v* observation) was included. The prognostic and predictive effects of histology subtypes according to stage were also assessed as exploratory analyses. Hazard ratios (HRs) and their 95% CIs were reported. We checked the validity of the proportional hazards assumption for each variable in the Cox model using the cumulative sums of martingale residuals (Data Supplement). Heterogeneity among trials was also evaluated using the χ^2 test, and results were reported via a forest plot. Survival analyses were performed on the completed patient cases. The level of statistical significance was set to .01 (pooled analysis). Statistical analyses were performed using SAS software (version 9.2; SAS Institute, Cary, NC).

RESULTS

Of 3,533 patients accrued to the IALT, JBR.10, ANITA, and CALGB 9633 (Alliance) trials, 1,766 had tissue samples and molecular data available for the LACE-Bio study (Data Supplement). These included 725 patients whose original diagnosis was adenocarcinoma. For 96 of these patients (defined as missing), histology slides were either unavailable (n = 80) or unsatisfactory for reclassification by one or both review pathologists (n = 16). Histologic slide review successfully reclassified 629 patient tumors into one of the invasive adenocarcinoma subtypes outlined in the IASLC/ATS/ERS classification.[2] In total, 582 patients with mixed-pattern tumor histology had their tumors reclassified into one of the predominant patterns: LEP (n = 24), ACN (n = 152), PAP (n = 99), MIP (n = 40), and SOL (n = 267). Because of missing covariates, seven patients in the observation arm were excluded from further correlative analysis, leaving 575 patients eligible for survival analyses. Patient characteristics of the analysis set were compared with those of 103 patients, including patients with unavailable slides (n = 80), slides unsatisfactory for reclassification (n = 16), or missing covariates (n = 7; Data Supplement). The only substantial difference observed was for T stage. Patients with invasive mucinous (n = 35) or other variants (n = 12) were not included in this comparison.

The clinical features of these 575 patients are listed in Table 1 overall and by randomized arm and are listed across trial in the Data Supplement. Median follow-up was 5.6 years (95% CI, 5.4 to 5.8). The most common subtype based on predominant histologic pattern was SOL (46%), followed by ACN (26%) and PAP (17%). LEP- and MIP-predominant tumors accounted for only 4% and 7% of the tumors, respectively (Data Supplement). However, slight variation in the distribution of tumor subtypes across trials was noted (*P* = .02); the proportions of LEP (8%) and MIP (1%) in CALGB 9633 (Alliance) were significantly higher and smaller, respectively, compared with those in other trials (LEP, < 5%; MIP, > 5%). Because of the small number of patients in some of the five subtypes and as previous reports have suggested (Data Supplement), three prognostic groupings (LEP, ACN/PAP, and MIP/SOL) were considered. The correlation of these three groups (LEP, n = 23 [4%]; ACN/PAP, n = 247 [43%]; and MIP/SOL, n = 305 [53%]) with clinical characteristics is reported in the Data Supplement. The proportion of patients with WHO performance status ≥ 1 was significant lower (17%) in the group with LEP-predominant tumors compared with the two other subtype groups (> 40%; *P* = .05).

Table 1. Demographic and Clinical Characteristics of Patients With Tumors Classified by IASLC/ATS/ERS System

	Total (N = 575)		Observation (surgery alone; n = 293)		ACT (n = 282)		
Characteristic	No.	%	No.	%	No.	%	P*
Sex							.18
Male	365	63	179	61	186	66	
Female	210	37	114	39	96	34	
Age, years							.56
< 55	200	35	101	34	99	35	
55 to 64	216	38	119	41	97	34	
≥ 65	159	28	73	25	86	31	
Stage							.87
I	310	54	152	52	158	56	
II	179	31	95	32	84	30	
III	86	15	46	16	40	14	
N stage							.78
N0	325	57	162	55	163	58	
N1	174	30	89	30	85	30	
N2	76	13	42	14	34	12	
T stage							.52
1	88	15	42	14	46	16	
2	446	78	230	79	216	77	
3 to 4	41	7	21	7	20	7	
Type of surgery							.08
Pneumonectomy	99	17	43	15	56	20	
Other	476	83	250	85	226	80	
WHO PS							.84
0	327	57	162	55	165	59	
1 to 2	248	43	131	45	117	41	
Adenocarcinoma subtype							.21
Lepidic	23	4	13	4	10	4	
Acinar	148	26	74	25	74	26	
Papillary	99	17	42	14	57	20	
Micropapillary	39	7	25	9	14	5	
Solid	266	46	139	47	127	45	

Abbreviations: ACT, adjuvant chemotherapy; ATS, American Thoracic Society; ERS, European Respiratory Society; IASLC, International Association for the Study of Lung Cancer; PS, performance status.
*χ^2 test was calculated from logistic regression model stratified by trial.

Prognostic Value of Subtypes

Among 575 patients, the number of events for OS, DFS, and SDFS was 269 (47%), 320 (56%), and 292 (51%), respectively. The prognostic impact was evaluated in the 293 patients in the observation arm. On univariable analysis, there was marginally significant prognostic difference between the three subtypes for OS (*P* =

Information downloaded from jco.ascopubs.org and provided by at Gazi Universitesi on May 1, 2015 from 194.27.18.18
Copyright © 2015 American Society of Clinical Oncology. All rights reserved.

.05; Fig 2A), but significant differences were seen for DFS ($P < .01$; Fig 2B) and SDFS ($P < .01$; Fig 2C), with the SOL and MIP subtypes showing worse outcomes. Similar results were observed when considering all five predominant histologic patterns (Data Supplement). Multivariable survival analyses (Table 2) showed no significant association for the primary end point (OS), with an HR of 0.70 (95% CI, 0.29 to 1.69) for ACN/PAP versus LEP and an HR of 0.96 (95% CI, 0.40 to 2.30) for MIP/SOL versus LEP (global test $P = .22$). However, there was a marginally significant association for DFS ($P = .05$) and SDFS ($P = .04$), with a poorer prognosis for MIP/SOL compared with LEP for DFS (HR, 1.32; 95% CI, 0.56 to 3.13) and SDFS (HR, 1.29; 95% CI, 0.55 to 3.07). This marginally significant difference observed for DFS and SDFS was mainly a result of the difference between ACN/PAP (as reference) and MIP/SOL, with HRs of 1.52 (95% CI, 1.09 to 2.11) and 1.58 (95% CI, 1.12 to 2.24) for DFS and SDFS, respectively. In addition, there was no heterogeneity of HRs across trials ($P > .70$). The HRs of MIP/SOL versus ACN/PAP for stage II and III disease were higher for all end points compared with those for stage I disease, with marginal effect for stage II. However, no significant interaction was observed ($P > .62$; Data Supplement).

Predictive Value of Subtypes for ACT Benefit

Because there were only 23 patients with the LEP-predominant subtype (observation, n = 6; ACT, n = 17), these patients were excluded from the predictive analysis, which included 552 patients (observation, n = 280; ACT, n = 272), with 263 deaths, 313 events, and 284 specific events. In univariable analysis, no significant benefit was seen from ACT in the ACN/PAP-predominant subtype for OS (unadjusted HR, 1.11; 95% CI, 0.76 to 1.62; log-rank $P = .51$), DFS (HR, 1.19; 95% CI, 0.84 to 1.68; $P = .37$), or SDFS (HR, 1.20; 95% CI, 0.83 to 1.72; $P = .42$). There was a nonsignificant trend toward an OS benefit from ACT in the MIP/SOL group (HR, 0.82; 95% CI, 0.59 to 1.14; $P = .12$), with a significant DFS benefit (HR, 0.65; 95% CI, 0.48 to 0.88; $P < .01$) and SDFS benefit (HR, 0.63; 95% CI, 0.46 to 0.87; $P < .01$; Fig 3). In multivariable analyses, the chemotherapy benefit was marginally significant for OS (HR, 0.71; 95% CI, 0.51 to 0.99; $P = .04$) for MIP/SOL but not ACN/PAP (HR, 1.00; 95% CI, 0.68 to 1.47; $P = .99$); however, the treatment by histology interaction was not significant (interaction $P = .18$). There was a significant benefit from ACT for the MIP/SOL subgroup for DFS (HR, 0.60; 95% CI, 0.44 to 0.82; $P = .001$)—but not for ACN/PAP (HR, 1.11; 95% CI, 0.78 to 1.57; $P = .57$; interaction $P = .009$)—and SDFS (HR, 0.59; 95% CI, 0.42 to 0.81; $P = .001$ *v* HR, 1.12; 95% CI, 0.77 to 1.61; $P = .56$; interaction $P = .01$; Table 3). However, there was significant heterogeneity of the treatment–subtype interaction (predictive effect) among trials (Data Supplement; $P \leq .05$). This is partly because the ANITA trial included only 40 patients. A sensitivity analysis was performed after removing the ANITA trial, and heterogeneity was no longer significant ($P > .10$; data not shown). Additional analyses evaluating the predictive effect of histologic subtypes for ACT benefit according to stage (I, II, or III) showed no significant interaction between histologic subtypes, treatment, and stage ($P > .82$). However, a difference of the treatment effect in MIP/SOL compared with ACN/PAP seems to be more important in stage II and III compared with stage I disease (Data Supplement).

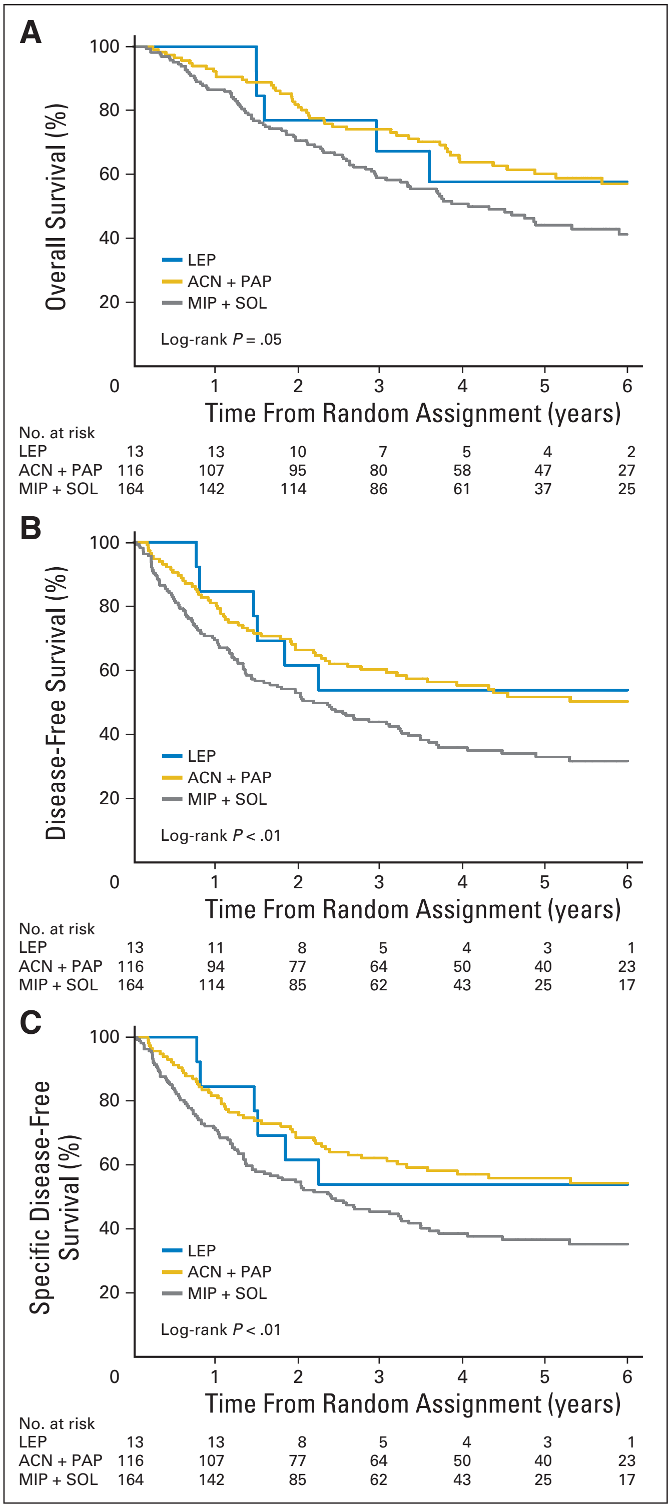

Fig 2. Survival curves in observation arm according to three predominant patterns (lepidic [LEP], acinar [ACN]/papillary [PAP], and micropapillary [MIP]/solid [SOL; n = 293]) for (A) overall (OS), (B) disease-free (DFS), and (C) specific disease-free survival (SDFS). *P* values from log-rank test. Hazard ratios (HRs), estimated through univariable Cox model stratified on trial, were as follows: ACN plus PAP/LEP: HR, 0.76 (95% CI, 0.32 to 1.80) and MIP plus SOL/LEP: HR, 1.12 (95% CI, 0.48 to 2.62) for OS; ACN plus PAP/LEP: HR, 0.99 (95% CI, 0.43 to 2.31) and MIP plus SOL/LEP: HR, 1.60 (95% CI, 0.69 to 3.68) for DFS; and ACN plus PAP/LEP: HR, 0.89 (95% CI, 0.38 to 2.08) and MIP plus SOL/LEP: HR, 1.47 (95% CI, 0.64 to 3.40) for SDFS.

 © 2015 by American Society of Clinical Oncology

Information downloaded from jco.ascopubs.org and provided by at Gazi Universitesi on May 1, 2015 from 194.27.18.18
Copyright © 2015 American Society of Clinical Oncology. All rights reserved.

Table 2. Multivariable Survival Analyses of Subtype Groupings in Patients Treated With Surgery Alone (observation arm; n = 293)

Subtype	OS				DFS				SDFS			
	No. of Deaths	HR	95% CI	*P*	No. of Events	HR	95% CI	*P*	No. of Events	HR	95% CI	*P*
LEP	6 of 13	1.0			6 of 13	1.0			6 of 13	1.0		
ACN/PAP	50 of 116	0.70	0.29 to 1.69		59 of 116	0.87	0.37 to 2.07		53 of 116	0.82	0.34 to 1.95	
MIP/SOL	89 of 164	0.96	0.40 to 2.30		109 of 164	1.32	0.56 to 3.13		101 of 164	1.29	0.55 to 3.07	
Overall test*				.22				.05				.04
Test of heterogeneity across trials†				.70‡				.86‡				.96‡
				.76§				.96§				.97§
				.95‖				.98‖				1.00‖

NOTE. Similar results were observed when using TNM stage instead of T and N of TNM in multivariable Cox regression model (data not shown).
Abbreviations: ACN, acinar; DFS, disease-free survival; HR, hazard ratio; LEP, lepidic; MIP, micropapillary; OS, overall survival; PAP, papillary; SDFS, specific disease-free survival; SOL, solid.
*Test of global association.
†ANITA (Adjuvant Navelbine International Trialist Association) trial was excluded to test heterogeneity across trials, because there was no patient with LEP subtype in this trial.
‡Test evaluating homogeneity of prognostic effect across trials in ACN/PAP group (*v* LEP group).
§Test evaluating homogeneity of prognostic effect across trials in MIP/SOL group (*v* LEP group).
‖Test evaluating joint hypotheses of ‡ and §.

DISCUSSION

This LACE-Bio study involving the largest cohort, to our knowledge, of multi-institutional tumor samples from patients from four pivotal international ACT trials has confirmed the clinical relevance of the IASLC/ATS/ERS lung adenocarcinoma classification, which is the basis for the fourth edition (2015) of the WHO classification system for lung adenocarcinoma. Our results showed that patients with invasive adenocarcinoma with a MIP/SOL-predominant pattern have marginally significantly poorer DFS and SDFS when compared with patients with an ACN/PAP pattern, although no significant differences were seen for OS. When the LEP-predominant pattern was excluded (exploratory analysis), patients with the MIP/SOL-predominant pattern had significantly poorer DFS and SDFS compared with those with the ACN/PAP pattern (DFS: HR, 1.52; 95% CI, 1.09 to 2.11; $P = .01$; SDFS: HR, 1.58; 95% CI, 1.11 to 2.23; $P = .01$). This also seems more obvious for stage II and III disease, even if no significant interaction between histology and stage exists. Predictive analyses showed that the effect of ACT on DFS and SDFS was significantly different in ACN/PAP and MIP/SOL subtypes, with the latter showing benefit. DFS has been validated as a surrogate end point of OS in the adjuvant setting.[15] The nonsignificant tests for the main end point (OS) may be explained by lack of power, smaller number of events for OS compared with DFS and SDFS, and dilution effect by noncancer deaths. We performed a bootstrap analysis (Data Supplement) to evaluate the stability of these last results. The bootstrap estimates and CIs of treatment effect in ACN/PAP and MIP/SOL subgroups were similar to those of the original analyses (Table 3). However, the predictive effect (subtype–treatment interaction) was found to be statistically significant in approximately half and three quarters of patient cases (1,000 bootstrap samples) at levels 1% and 5%, respectively, for DFS and SDFS (data not shown). In addition, the predictive analyses according to stage showed results similar to those based on the overall patient cohort, despite marked limitation by the small number of patient cases in each stage group. Therefore, we have shown the first evidence to our knowledge that MIP/SOL-predominant histology is a promising predictive marker for benefit from ACT in DFS and SDFS in patients with early-stage lung adenocarcinoma; however, more analyses are needed to confirm these findings.

The histology of lung adenocarcinoma reveals marked heterogeneity in growth patterns; however, the clinical relevance of this has never been established. Part of this failure was resulted from the difficulty in defining the extent and components of this heterogeneity, resulting in the use of mixed subtype as an inclusive category for mixed-pattern tumors in the third edition (2004) of the WHO classification. Because most (> 80% to 90%) lung adenocarcinomas belong to this subtype, the significance of the various growth patterns has largely been ignored, except for bronchioloalveolar carcinoma, which is represented by a pure LEP pattern. In contrast, the IASLC/ATS/ERS classification recognizes the importance of predominant pattern. Since its publication, 14 studies worldwide (Data Supplement) have validated the clinical relevance of this new classification system. These studies have confirmed a 100% survival outcome of adenocarcinoma in situ with 100% LEP growth pattern[5,6,8,16-21] and consistently reported that the worst survival outcomes were seen in patients with invasive adenocarcinoma with predominately MIP and SOL patterns (Data Supplement).

The importance of the MIP growth pattern in lung adenocarcinoma has only been recognized in the IASLC/ATS/ERS classification, although its association with poorer prognosis in breast cancer is well known.[22] The prevalence of the MIP-predominant subtype in the LACE-Bio study was 7% (range, 1% to 10% across trials). This is consistent with the reported prevalence across 14 studies (Data Supplement). The importance of this subtype was recently highlighted in small (≤ 2 cm) lung adenocarcinomas.[23] Limited resection of these small lung tumors with ≥ 5% MIP component was associated with significantly greater risk of recurrence.

The SOL pattern is equivalent to poorly differentiated carcinomas without any morphologically recognizable differentiation features. In the LACE-Bio study, the SOL-predominant pattern represented 46% (range, 42% to 58%) of the mixed-pattern invasive adenocarcinomas. Prevalence of the predominantly SOL subtype ranged from 7% to 40% among the 13 studies (Data Supplement), with higher rates noted in studies that included patients with higher-stage disease.

© 2015 by American Society of Clinical Oncology

Information downloaded from jco.ascopubs.org and provided by at Gazi Universitesi on May 1, 2015 from 194.27.18.18
Copyright © 2015 American Society of Clinical Oncology. All rights reserved.

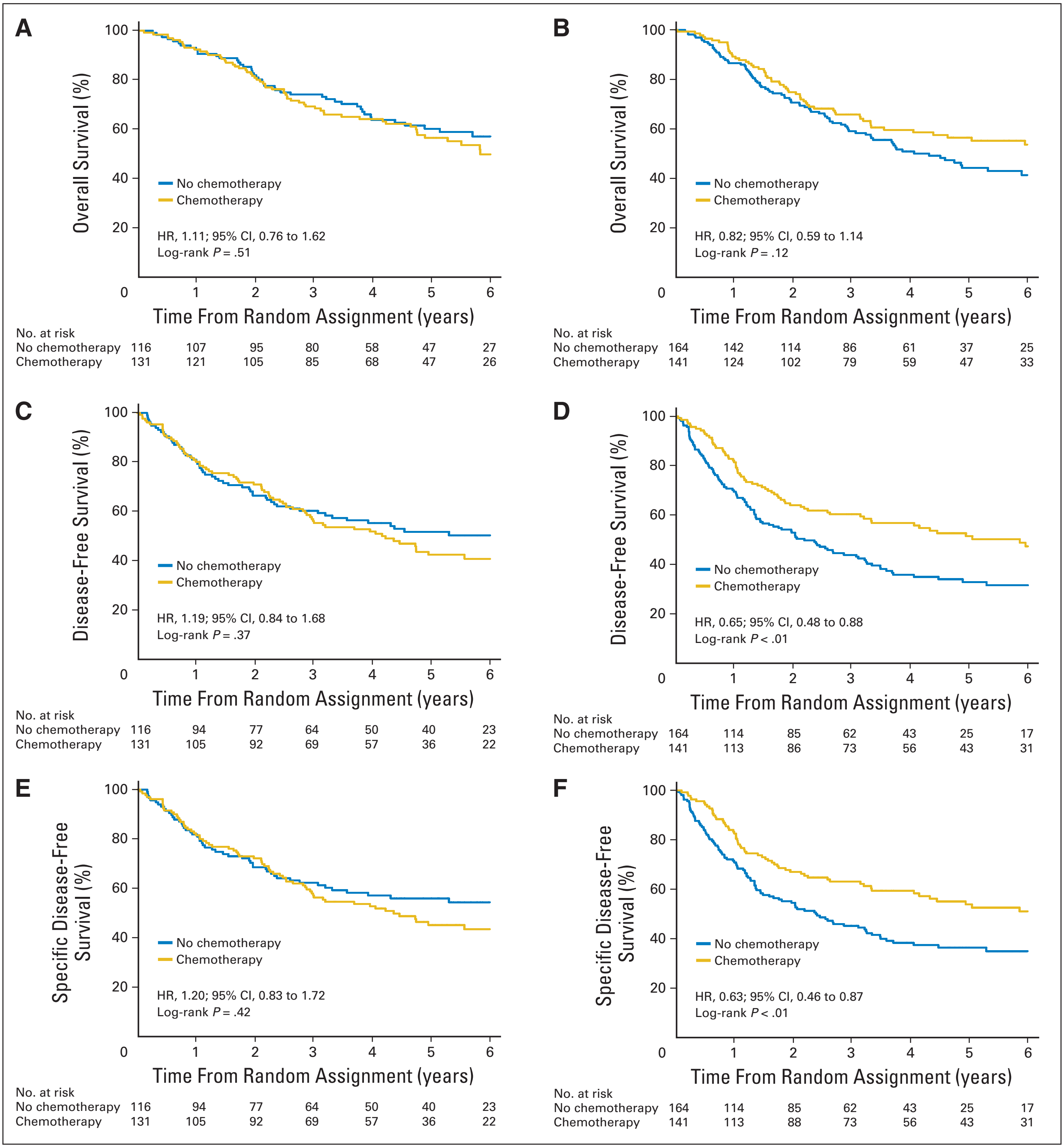

Fig 3. Survival curves according to treatment arm (chemotherapy *v* observation) in (A, C, E) acinar/papillary and (B, D, F) micropapillary/solid subgroups for (A, B) overall, (C, D) disease-free, and (E, F) specific disease-free survival. *P* values from log-rank test and hazard ratio (HR) and 95% CIs of treatment effect, estimated through univariable Cox model stratified on trial, were reported for each subgroup and end point.

In the IASLC/ATS/ERS classification, one of the pathologic recommendations for invasive adenocarcinoma is to assess the histologic pattern semiquantitatively in 5% increments and choose a single predominant pattern to subtype the tumor.[2] This is usually performed on HE sections of multiple blocks sampled from the tumor. In our study, only one unselected representative section of the tumor was available for review. However, in a study by an international panel of 26 pulmonary pathologists, examination of one section per patient case demonstrated a high degree of interobserver consistency in recognizing the five typical patterns adopted in the IASLC/ATS/ERS classification,

Information downloaded from jco.ascopubs.org and provided by at Gazi Universitesi on May 1, 2015 from 194.27.18.18
Copyright © 2015 American Society of Clinical Oncology. All rights reserved.

Table 3. HRs Estimating Treatment Effect (chemotherapy *v* observation) per Subgroup (ACN/PAP *v* MIP/SOL) for OS, DFS, and SDFS (n = 552)

Subgroup	No. of Deaths: Chemotherapy	No. of Deaths: Observation	HR	95% CI	*P*	Interaction: HR	Interaction: 95% CI	Interaction: *P*
OS						0.71	0.43 to 1.17	.18
ACN/PAP	60 of 131	50 of 116	1.00	0.68 to 1.47	.99			
MIP/SOL	64 of 141	89 of 164	0.71	0.51 to 0.99	.04			
Test of heterogeneity across trials					.27*			
					.16†			
					.18‡			
DFS						0.54	0.34 to 0.86	.009
ACN/PAP	75 of 131	59 of 116	1.11	0.78 to 1.57	.57			
MIP/SOL	70 of 141	109 of 164	0.60	0.44 to 0.82	.001			
Test of heterogeneity across trials					.10*			
					.07†			
					.05‡			
SDFS								
ACN/PAP	68 of 131	53 of 116	1.12	0.77 to 1.61	.56	0.53	0.32 to 0.86	.01
MIP/SOL	62 of 141	101 of 164	0.59	0.42 to 0.81	.001			
Test of heterogeneity across trials					.26*			
					.04†			
					.06‡			

NOTE. Similar results were observed when using TNM stage instead of T and N of TNM in multivariable Cox regression model (data not shown).
Abbreviations: ACN, acinar; DFS, disease-free survival; HR, hazard ratio; MIP, micropapillary; OS, overall survival; PAP, papillary; SDFS, specific disease-free survival; SOL, solid.
*Test evaluating homogeneity of treatment effect across trials in ACN/PAP group.
†Test evaluating homogeneity of treatment effect across trials in MIP/SOL group.
‡Test evaluating joint hypotheses of * and †.

with a mean kappa score (± standard deviation) of 0.77 ± 0.77.[4] Importantly, this study also demonstrated that recognition of a predominant pattern could be reached with high concordance, especially for the predominantly SOL pattern. In another study involving five pulmonary pathologists using 100 consecutive patient cases, *P* values for nominating the predominant pattern ranged from .44 to .72, once again with the highest concordance achieved for the SOL pattern.[24] Interobserver variability was significantly reduced after training.[25] Recognizing these limitations of the study and the ability of pathologists to subtype lung adenocarcinoma, we believe routine use and continuing educational efforts will greatly improve accuracy in the future application of this new classification system.

Overall, it is estimated that > 50% of patients with early-stage non–small-cell lung cancer will develop recurrence after their primary surgery,[26,27] and identifying prognostic factors beyond stage is crucial to select patients who need adjuvant therapies. There have been intensive studies on molecular prognostic markers, but so far, no markers have been sufficiently validated for routine clinical use.[28,29] LACE-Bio validation of promising prognostic and predictive markers, including *ERCC1* and *KRAS* mutations, reported from various ACT trials has so far been unsuccessful.[30,31] The results of our study represent the first markers, to our knowledge, from the LACE-Bio project that suggest a significant predictive value for survival benefit from ACT in patients with early-stage lung adenocarcinoma. Because histologic classification is fundamental for patient diagnosis, our results demonstrate the necessity of applying the IASLC/ATS/ERS subtype classification for lung adenocarcinoma in routine practice.

In summary, the LACE-Bio study involving patients from four pivotal randomized ACT trials has confirmed that early-stage lung adenocarcinomas with a MIP- or SOL-predominant pattern have poorer DFS and SDFS. More importantly, our study has shown that these patterns seem to be predictive of a differential survival benefit from ACT. Routine adoption of the subtype classification in clinical diagnosis and prospective validation in future adjuvant clinical trials are warranted.

AUTHORS' DISCLOSURES OF POTENTIAL CONFLICTS OF INTEREST

Disclosures provided by the authors are available with this article at www.jco.org.

AUTHOR CONTRIBUTIONS

Conception and design: Ming-Sound Tsao, Gwénaël Le Teuff, Frances A. Shepherd, Lesley Seymour, Robert Kratzke, Stephen L. Graziano, Rafael Rosell, Thierry Le-Chevalier, Jean-Pierre Pignon, Jean-Charles Soria, Elisabeth M. Brambilla
Financial support: Ming-Sound Tsao, Jean-Charles Soria, Elisabeth M. Brambilla
Administrative support: Ming-Sound Tsao, Lesley Seymour, Stephen L. Graziano, Jean-Pierre Pignon, Jean-Charles Soria, Elisabeth M. Brambilla
Provision of study materials or patients: Ming-Sound Tsao, Sylvie Lantuejoul, Frances A. Shepherd, Lesley Seymour, Robert Kratzke, Stephen L. Graziano, Helmut H. Popper, Rafael Rosell, Jean-Yves Douillard, Thierry Le-Chevalier, Jean-Charles Soria, Elisabeth M. Brambilla
Collection and assembly of data: Ming-Sound Tsao, Gwénaël Le Teuff, Sylvie Lantuejoul, Lesley Seymour, Stephen L. Graziano, Helmut H. Popper, Rafael Rosell, Jean-Pierre Pignon, Jean-Charles Soria, Elisabeth M. Brambilla
Data analysis and interpretation: Ming-Sound Tsao, Sophie Marguet, Gwénaël Le Teuff, Lesley Seymour, Jean-Yves Douillard, Jean-Pierre Pignon, Jean-Charles Soria, Elisabeth M. Brambilla
Manuscript writing: All authors
Final approval of manuscript: All authors

© 2015 by American Society of Clinical Oncology

Information downloaded from jco.ascopubs.org and provided by at Gazi Universitesi on May 1, 2015 from 194.27.18.18
Copyright © 2015 American Society of Clinical Oncology. All rights reserved.

REFERENCES

1. Travis WD, Brambilla E, Muller-Hermelink HK, et al: (eds): WHO Classification of Tumours 2004: Pathology and Genetics of Tumours of the Lung, Pleura, Thymus and Heart. Lyon, France, IARC Press, 2004

2. Travis WD, Brambilla E, Noguchi M, et al: International Association for the Study of Lung Cancer/American Thoracic Society/European Respiratory Society international multidisciplinary classification of lung adenocarcinoma. J Thorac Oncol 6:244-285, 2011

3. Travis WD, Brambilla E, Burke A, et al (eds): WHO Classification of the Tumours of the Lung, Pleura, Thymus and Heart (ed 4). Lyon, France, IARC Press, 2015

4. Thunnissen E, Beasley MB, Borczuk AC, et al: Reproducibility of histopathological subtypes and invasion in pulmonary adenocarcinoma: An international interobserver study. Mod Pathol 25:1574-1583, 2012

5. Yoshizawa A, Motoi N, Riely GJ, et al: Impact of proposed IASLC/ATS/ERS classification of lung adenocarcinoma: Prognostic subgroups and implications for further revision of staging based on analysis of 514 stage I cases. Mod Pathol 24:653-664, 2011

6. Russell PA, Wainer Z, Wright GM, et al: Does lung adenocarcinoma subtype predict patient survival? A clinicopathologic study based on the new International Association for the Study of Lung Cancer/American Thoracic Society/European Respiratory Society international multidisciplinary lung adenocarcinoma classification. J Thorac Oncol 6:1496-1504, 2011

7. Warth A, Muley T, Meister M, et al: The novel histologic International Association for the Study of Lung Cancer/American Thoracic Society/European Respiratory Society classification system of lung adenocarcinoma is a stage-independent predictor of survival. J Clin Oncol 30:1438-1446, 2012

8. Yoshizawa A, Sumiyoshi S, Sonobe M, et al: Validation of the IASLC/ATS/ERS lung adenocarcinoma classification for prognosis and association with EGFR and KRAS gene mutations: Analysis of 440 Japanese patients. J Thorac Oncol 8:52-61, 2013

9. Arriagada R, Bergman B, Dunant A, et al: Cisplatin-based adjuvant chemotherapy in patients with completely resected non-small-cell lung cancer. N Engl J Med 350:351-360, 2004

10. Douillard JY, Rosell R, De Lena M, et al: Adjuvant vinorelbine plus cisplatin versus observation in patients with completely resected stage IB-IIIA non-small-cell lung cancer (Adjuvant Navelbine International Trialist Association [ANITA]): A randomised controlled trial. Lancet Oncol 7:719-727, 2006

11. Winton T, Livingston R, Johnson D, et al: Vinorelbine plus cisplatin vs. observation in resected non-small-cell lung cancer. N Engl J Med 352:2589-2597, 2005

12. Strauss GM, Herndon JE 2nd, Maddaus MA, et al: Adjuvant paclitaxel plus carboplatin compared with observation in stage IB non–small-cell lung cancer: CALGB 9633 with the Cancer and Leukemia Group B, Radiation Therapy Oncology Group, and North Central Cancer Treatment Group study sroups. J Clin Oncol 26:5043-5051, 2008

13. Travis WD, Brambilla E, Riely GJ: New pathologic classification of lung cancer: Relevance for clinical practice and clinical trials. J Clin Oncol 31: 992-1001, 2013

14. Schemper M, Smith TL: A note on quantifying follow-up in studies of failure time. Control Clin Trials 17:343-346, 1996

15. Mauguen A, Pignon JP, Burdett S, et al: Surrogate endpoints for overall survival in chemotherapy and radiotherapy trials in operable and locally advanced lung cancer: A re-analysis of meta-analyses of individual patients' data. Lancet Oncol 14:619-626, 2013

16. Woo T, Okudela K, Mitsui H, et al: Prognostic value of the IASLC/ATS/ERS classification of lung adenocarcinoma in stage I disease of Japanese cases. Pathol Int 62:785-791, 2012

17. Gu J, Lu C, Guo J, et al: Prognostic significance of the IASLC/ATS/ERS classification in Chinese patients: A single institution retrospective study of 292 lung adenocarcinoma. J Surg Oncol 107:474-480, 2013

18. Song Z, Zhu H, Guo Z, et al: Prognostic value of the IASLC/ATS/ERS classification in stage I lung adenocarcinoma patients: Based on a hospital study in China. Eur J Surg Oncol 39:1262-1268, 2013

19. Tsuta K, Kawago M, Inoue E, et al: The utility of the proposed IASLC/ATS/ERS lung adenocarcinoma subtypes for disease prognosis and correlation of driver gene alterations. Lung Cancer 81:371-376, 2013

20. Zhang J, Wu J, Tan Q, et al: Why do pathological stage IA lung adenocarcinomas vary from prognosis? A clinicopathologic study of 176 patients with pathological stage IA lung adenocarcinoma based on the IASLC/ATS/ERS classification. J Thorac Oncol 8:1196-1202, 2013

21. Yanagawa N, Shiono S, Abiko M, et al: New IASLC/ATS/ERS classification and invasive tumor size are predictive of disease recurrence in stage I lung adenocarcinoma. J Thorac Oncol 8:612-618, 2013

22. Pettinato G, Manivel CJ, Panico L, et al: Invasive micropapillary carcinoma of the breast: Clinicopathologic study of 62 cases of a poorly recognized variant with highly aggressive behavior. Am J Clin Pathol 121:857-866, 2004

23. Nitadori J, Bograd AJ, Kadota K, et al: Impact of micropapillary histologic subtype in selecting limited resection vs lobectomy for lung adenocarcinoma of 2cm or smaller. J Natl Cancer Inst 105: 1212-1220, 2013

24. Warth A, Stenzinger A, von Brünneck AC, et al: Interobserver variability in the application of the novel IASLC/ATS/ERS classification for pulmonary adenocarcinomas. Eur Respir J 40:1221-1227, 2012

25. Warth A, Cortis J, Fink L, et al: Training increases concordance in classifying pulmonary adenocarcinomas according to the novel IASLC/ATS/ERS classification. Virchows Arch 461:185-193, 2012

26. Chansky K, Sculier JP, Crowley JJ, et al: The International Association for the Study of Lung Cancer Staging Project: Prognostic factors and pathologic TNM stage in surgically managed non-small cell lung cancer. J Thorac Oncol 4:792-801, 2009

27. Pignon JP, Tribodet H, Scagliotti GV, et al: Lung adjuvant cisplatin evaluation: A pooled analysis by the LACE Collaborative Group. J Clin Oncol 26:3552-3559, 2008

28. Zhu CQ, Shih W, Ling CH, et al: Immunohistochemical markers of prognosis in non-small cell lung cancer: A review and proposal for a multiphase approach to marker evaluation. J Clin Pathol 59:790-800, 2006

29. Tsao MS, Jablons DM: Molecular prognostication of non-small cell lung cancer. Semin Thorac Cardiovasc Surg 25:4-7, 2013

30. Friboulet L, Olaussen KA, Pignon JP, et al: ERCC1 isoform expression and DNA repair in non-small-cell lung cancer. N Engl J Med 368:1101-1110, 2013

31. Shepherd FA, Domerg C, Hainaut P, et al: Pooled analysis of the prognostic and predictive effects of *KRAS* mutation status and *KRAS* mutation subtype in early-stage resected non–small-cell lung cancer in four trials of adjuvant chemotherapy. J Clin Oncol 31:2173-2181, 2013

Support

Supported by research grants from Ligue Nationale Contre le Cancer and le Programme National d'Excellence Specialisé Cancer du Poumon de l'Institut National du Cancer (France), National Cancer Institute (United States), and Canadian Cancer Society; by unrestricted grant from sanofi-aventis; by personal funding from the investigators; by the Gustave Roussy Foundation; by the Princess Margaret Cancer Foundation; and by European CURELUNG Contract No. EU-FP7.

Information downloaded from jco.ascopubs.org and provided by at Gazi Universitesi on May 1, 2015 from 194.27.18.18
Copyright © 2015 American Society of Clinical Oncology. All rights reserved.

AUTHORS' DISCLOSURES OF POTENTIAL CONFLICTS OF INTEREST

Subtype Classification of Lung Adenocarcinoma Predicts Benefit From Adjuvant Chemotherapy in Patients Undergoing Complete Resection

The following represents disclosure information provided by authors of this manuscript. All relationships are considered compensated. Relationships are self-held unless noted. I = Immediate Family Member, Inst = My Institution. Relationships may not relate to the subject matter of this manuscript. For more information about ASCO's conflict of interest policy, please refer to www.asco.org/rwc or jco.ascopubs.org/site/ifc.

Ming-Sound Tsao
No relationship to disclose

Sophie Marguet
No relationship to disclose

Gwénaël Le Teuff
No relationship to disclose

Sylvie Lantuejoul
Consulting or Advisory Role: Pfizer, Novartis, Bristol-Myers Squibb, Merck, Roche
Research Funding: Pfizer

Frances A. Shepherd
Stock or Other Ownership: Eli Lilly, AstraZeneca
Honoraria: Eli Lilly, AstraZeneca, Bristol-Myers Squibb, Merck Serono, Roche/Genentech, Merck/Schering Plough, Boehringer Ingelheim
Consulting or Advisory Role: Eli Lilly, AstraZeneca, GlaxoSmithKline, Boehringer Ingelheim
Research Funding: Boehringer Ingelheim (Inst)
Travel, Accommodations, Expenses: Novartis

Lesley Seymour
Stock or Other Ownership: AstraZeneca
Consulting or Advisory Role: Boehringer Ingelheim
Research Funding: Pfizer (Inst), AstraZeneca (Inst), Novartis (Inst), Astex Therapeutics (Inst), Novartis (Inst), Innate Pharma (Inst)

Robert Kratzke
No relationship to disclose

Stephen L. Graziano
Consulting or Advisory Role: Helsinn

Helmut H. Popper
Consulting or Advisory Role: Novartis, Hoffman-La Roche, Pfizer, Eli Lilly

Rafael Rosell
No relationship to disclose

Jean-Yves Douillard
Honoraria: AstraZeneca, Roche, Boehringer Ingelheim
Consulting or Advisory Role: AstraZeneca, Roche, Boehringer Ingelheim, GlaxoSmithKline, Novartis
Research Funding: Merck Serono (Inst)

Thierry Le-Chevalier
No relationship to disclose

Jean-Pierre Pignon
No relationship to disclose

Jean-Charles Soria
No relationship to disclose

Elisabeth M. Brambilla
No relationship to disclose

© 2015 by American Society of Clinical Oncology
Information downloaded from jco.ascopubs.org and provided by at Gazi Universitesi on May 1, 2015 from 194.27.18.18
Copyright © 2015 American Society of Clinical Oncology. All rights reserved.

Acknowledgment

Presented in part at the 15th World Conference on Lung Cancer, Sydney, New South Wales, Australia, October 26-29, 2014.

© 2015 by American Society of Clinical Oncology

Information downloaded from jco.ascopubs.org and provided by at Gazi Universitesi on May 1, 2015 from 194.27.18.18
Copyright © 2015 American Society of Clinical Oncology. All rights reserved.
