# Supplementary material for: Efficacy and Benefit of Postoperative Chemotherapy in Micropapillray or Solid Predominant Pattern in Stage IB Lung Adenocarcinoma: A Systematic Review and Meta-Analysis
Source: Front Surg. 2021 Dec 21;8:795921. doi: 10.3389/fsurg.2021.795921 (PMC8724201; doi:10.3389/fsurg.2021.795921)
Supplement: Supplementary file 1 [file Data_Sheet_1.ZIP › ╬─╧╫/129.pdf]

Original Article

Micropapillary or solid pattern predicts recurrence free survival benefit from adjuvant chemotherapy in patients with stage IB lung adenocarcinoma

Minjie Ma[1], Yunlang She[2], Yijiu Ren[2], Chenyang Dai[2], Lei Zhang[2], Huikang Xie[3], Chunyan Wu[3], Minglei Yang[4], Dong Xie[2], Chang Chen[2]

[1]Department of Thoracic Surgery, The First Hospital of Lanzhou University, Lanzhou, China; [2]Department of Thoracic Surgery, [3]Department of Pathology, Shanghai Pulmonary Hospital, Tongji University School of Medicine, Shanghai 200433, China; [4]Department of Thoracic Surgery, Ningbo No. 2 Hospital, Ningbo 315000, China

Contributions: (I) Conception and design: M Ma, C Chen; (II) Administrative support: D Xie; (III) Provision of study materials or patients: C Chen; (IV) Collection and assembly of data: Y She, Y Ren, C Dai, L Zhang, H Xie, C Wu, M Yang; (V) Data analysis and interpretation: M Ma, D Xie; (VI) Manuscript writing: All authors; (VII) Final approval of manuscript: All authors.

Correspondence to: Dong Xie, MD, PhD; Chang Chen, MD, PhD. Zhengmin Road 507, Shanghai 200433, China. Email: kongduxd@163.com; chenthoracic@163.com.

Background: Our study aimed to evaluate the prognostic significance and adjuvant chemotherapy (ACT) benefits of a micropapillary/solid (MS) pattern in patients with stage IB lung adenocarcinoma.

Methods: Patients with pathologically-confirmed stage IB adenocarcinoma who underwent surgical resection between January 2009 and December 2011 were included. The tumors were reclassified into three categories: MS patterns absent (MS–); non-predominant MS patterns (MS+); predominant MS (MS++). The correlations of prognosis and ACT with recurrence-free survival (RFS) were evaluated.

Results: Overall, 497 (MS–, n=269; MS+, n=177; MS++, n=51) patients were enrolled in the study. In univariate analysis, the MS+ [hazard ratio (HR), 1.437; 95% confidence interval (CI), 1.030–2.006; P=0.033] and MS++ (HR, 2.818; 95% CI, 1.792–4.432; P<0.001) groups had significantly poor prognosis compared with MS- group. Multivariate analysis revealed that age ≥65 (HR, 1.504; 95% CI, 1.077–2.099; P=0.017), serum level of carcinoembryonic antigen (CEA) ≥10 ng/mL (HR, 1.658; 95% CI, 1.048–2.623; P=0.031) and MS++ (HR, 2.529; 95% CI, 1.550–4.126; P<0.001) were significant prognostic factors. Furthermore, subgroup analysis showed that MS++ patients but not MS– and MS+ derived RFS (recurrence-free survival) benefit from ACT (HR, 0.357; 95% CI, 0.152–0.836; P=0.018).

Conclusions: MS pattern successfully differentiated the prognosis difference among stage IB lung adenocarcinomas and identified patients who benefitted from ACT.

Keywords: Lung adenocarcinoma; micropapillary pattern; solid pattern; adjuvant chemotherapy (ACT); prognosis

Submitted Apr 18, 2018. Accepted for publication Aug 08, 2018.
doi: 10.21037/jtd.2018.08.64
View this article at: http://dx.doi.org/10.21037/jtd.2018.08.64

Introduction

Non-small cell lung cancer (NSCLC) is the leading cause of cancer-related death around the world (1). Tumor recurrence is the major cause of treatment failure after surgical resection for lung cancer (2). Several randomized controlled trials and meta-analysis (3-5) have demonstrated that adjuvant chemotherapy (ACT) considerably improved survival in patients with resected stage II and IIIA NSCLC. However, the benefit of ACT remains controversial for patients with resected stage IB NSCLC (6). Patients with tumor size >4 cm are recommended receiving ACT based on a prospective randomized clinical trial (6).

© Journal of Thoracic Disease. All rights reserved.

However, according to the 8th tumor node metastasis (TNM) classification (7), the tumors with size >4 cm is not including in stage IB anymore. Therefore, re-evaluation of the necessity of ACT in stage IB lung adenocarcinoma is urgent.

Besides, the new classification recommends subtyping of invasive lung adenocarcinoma on the basis of the predominant histologic pattern presented in the resected tumor (8). This classification has been validated for its prognostic effect in stage I NSCLC (9-13), which formed the basis of the 2015 World Health Organization classification of lung tumors (14). Previous studies showed that invasive adenocarcinomas with predominant micropapillary or solid (MS) patterns were associated with poor prognosis and ACT benefits (15,16). In this study, we aimed to evaluate the predictive role of MS pattern in ACT for patients with stage IB lung adenocarcinoma.

Methods

Patients

The Shanghai Pulmonary Hospital institutional ethics committee approved this retrospective study (IRB No. K18-101). Between January 2009 and December 2010, all patients who underwent complete resection for pathological stage IB lung adenocarcinoma at Shanghai Pulmonary Hospital were retrospectively reviewed. Patients who had multiple primary lung cancers or incomplete follow-up data were excluded. The T and N descriptors were derived from pathological reports, and all patients were re-staged based on the 8th TNM classification (7). The platinum-based ACT (cisplatin plus docetaxel or pemetrexed) was performed in patients with high-risk factors including poor differentiated tumor, visceral pleural invasion, vascular invasion and wedge resection. Age, performance status and patients' preference were also considered. No mortality related to chemotherapy was detected among the patients who underwent 4 cycles of ACT.

Patients were followed up every 3 months for the first year after the surgery and at 6-month intervals thereafter. For patients who were followed up in local health facilities, survival status and examination results were collected by telephone or email. Tumor locoregional recurrence or distant metastasis was diagnosed using chest computed tomography (CT), brain magnetic resonance imaging (MRI), and bone scintigraphy as well as ultrasound and/or abdominal CT. Recurrence-free survival (RFS) was defined as time length from surgery to tumor recurrence or the last follow-up.

Histologic evaluation

All specimens were formalin fixed and stained with hematoxylin and eosin (HE). Two pathologists retrospectively re-evaluated all slides together at a multi-headed microscope and discussed until consensus was achieved. According to the new IASLC/ATS/ERS classification (8,14), presence of each histological pattern component was recorded in 5% increments. The adenocarcinomas patterns were categorized as lepidic, papillary, acinar, micropapillary, solid (*Figure 1*). The invasive mucinous adenocarcinoma (n=2) and other variants subtypes (n=7) were excluded from the statistical analyses to focus on MS subtypes. The predominant pattern was defined as pattern with largest percentage. The tumors were classified into three categories: MS patterns absent (MS−); non-predominant MS patterns (MS+); predominant MS (MS++). All slides were assessed for visceral pleural invasion (VPI), and elastic stain was used if the results on HE sections were indeterminate.

Statistical analysis

Categorical variables were compared using the χ^2 test and Fisher's exact test as appropriate, and continuous variables were compared using independent *t*-test. RFS was assessed using the Kaplan-Meier method, with a log-rank test to determine the significance. Univariate and multivariate survival analysis were conducted using the Cox proportional hazards model. Variables with $P \le 0.1$ in univariate analysis were entered into the multivariate analysis with a backward stepwise method, and variables with $P < 0.05$ in the multivariate analysis remained in the model. All statistical analyses were performed using SPSS version 22.0 (IBM, Corporation, Armonk, NY, USA).

Results

Clinicopathologic characteristics

Baseline characteristics and clinical features of these patients are listed in *Table 1*. Four hundred and ninety-seven patients were included and 105 were excluded for the presence of multiple lung cancers (n=37), incomplete follow-up data (n=68). The median age at diagnosis was 61 years (range, 34–90 years), and most patients had no smoking history

© Journal of Thoracic Disease. All rights reserved.

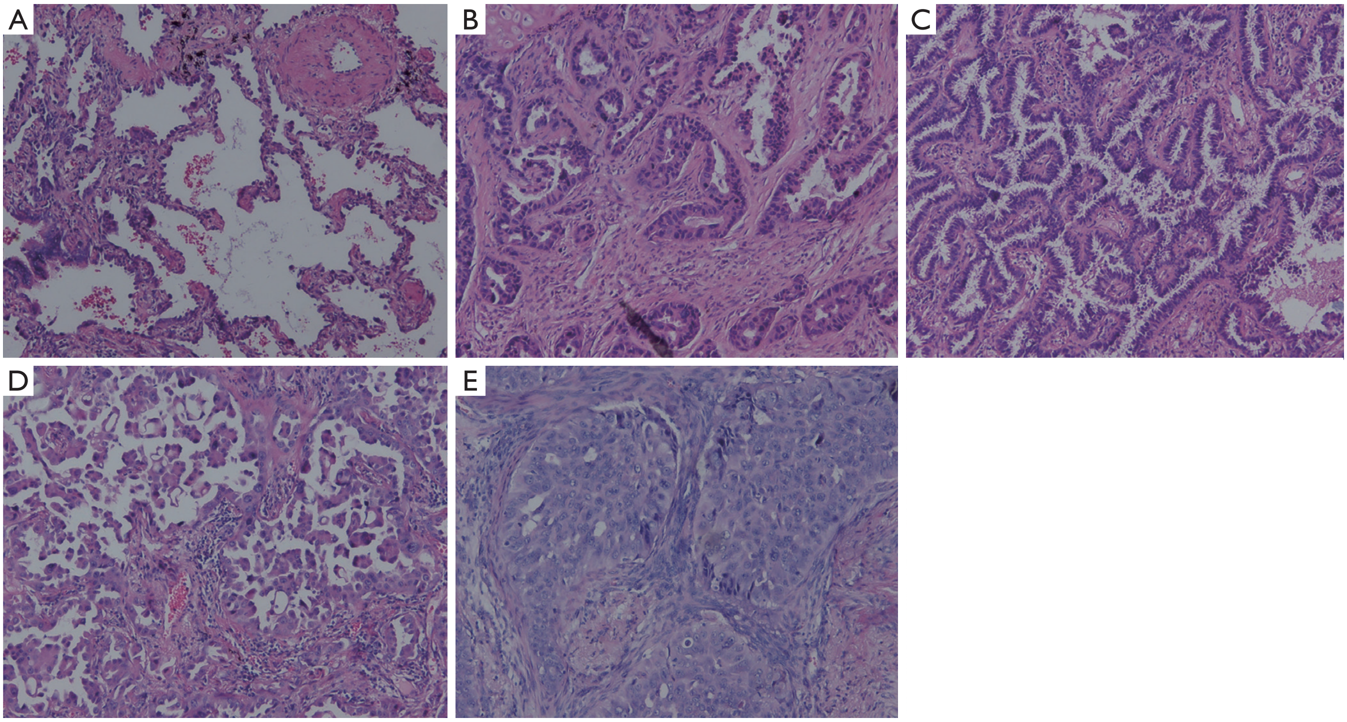

Figure 1 Architectural demonstration of five patterns of adenocarcinoma in the grading system: (A) lepidic; (B) acinar; (C) papillary; (D) micropapillary; and (E) solid. (HE stain, ×100).

(n=398) with normal serum level of carcinoembryonic antigen (CEA) (n=455). Tumor size was greater than 3 cm in 134 (27.0%) patients, and VPI was presented in 435 (87.5%) patients. Twenty-two (4.4%) patients underwent sublobar resection, which was more likely to be performed for older patients due to limited lung function. Among the 363 patients with tumor size less than 3 cm, 357 were staged as IB because of the existence of pleural invasion, and the other 6 were due to tumor invasion beyond lobar bronchus. Histopathological assessment shown that 269 (54.1%) patients were MS–; 177 (35.6%) were MS+; 51 (10.3%) were MS++. And the median percentage of MS component was 10% (5–25%) and 55% (25–75%) in the MS+ and MS++ group, respectively. Among all patients, ACT was performed in 149 (30.0%) patients. The ACT was significantly associated with older age (P=0.007) and MS pattern (P=0.007). The median follow-up time was 38.6 months (range, 1.7–96.6 months).

Prognostic value of MS pattern

During the follow-up, tumor recurrence was observed in 165 (33.2%) patients with 77 (28.6%) in MS– group, 63 (35.6%) in MS+ group, and 25 (49.0%) in MS++ group. The cox regression analysis with tumor recurrence as the endpoint was summarized in *Table 2*. MS pattern, age ≥65 (P=0.043), and CEA serum level ≥10 ng/mL (P=0.003) were independent risk factors for tumor recurrence. Interestingly, chemotherapy was not associated with the tumor recurrence when analysis was based on overall patients.

Among the three subgroups of MS pattern, RFS was significantly different with patients in MS- subgroup showing the best outcome (P<0.001; *Figure 2A*), and similar results were obtained when patients treated with surgery alone (P<0.001; *Figure 2B*). In univariate analysis, the MS+ [hazard ratio (HR), 1.437; 95% confidence interval (CI), 1.030–2.006; P=0.033] and MS++ (HR, 2.818; 95% CI, 1.792–4.432; P<0.001) groups had significantly poor prognosis compared with MS– group. In multivariate survival analysis, HR was 1.266 (95% CI, 0.898–1.785, P=0.178) for MS+ versus MS– and 2.529 (95% CI, 1.550–4.126, P<0.001) for MS++ versus MS–.

Predictive value of subgroup for ACT benefit

When analysis was performed for overall patients, no

© Journal of Thoracic Disease. All rights reserved.

Table 1 Characteristics between patients with surgical intervention alone and with adjuvant chemotherapy

Characteristic	All patients (N=497)	Surgical intervention alone (N=348)	With adjuvant chemotherapy (N=149)	P
Age, y, no. (%)				0.007
<65	355 (71.4)	261 (75.0)	94 (63.1)	
≥65	142 (28.6)	87 (25.0)	55 (36.9)	
Sex, no. (%)				0.356
Female	288 (57.9)	197 (56.6)	58 (38.9)	
Male	209 (42.1)	151 (43.4)	91 (61.1)	
Smoking history, no. (%)				0.680
Yes	99 (19.9)	71 (20.4)	28 (18.8)	
No	398 (80.1)	277 (79.6)	121 (81.2)	
CEA (ng/mL), no. (%)				0.886
<10	455 (91.5)	319 (91.7)	136 (91.3)	
≥10	42 (8.5)	29 (8.3)	13 (8.7)	
Tumor size (cm), no. (%)				0.484
≤3	363 (73.0)	251 (72.1)	112 (75.2)	
>3	134 (27.0)	97 (27.9)	37 (24.8)	
Visceral pleural invasion, no. (%)				0.057
Absent	62 (12.5)	37 (10.6)	25 (16.8)	
Present	435 (87.5)	311 (89.4)	124 (83.2)	
Differentiation grade, no. (%)				<0.001
Well	147 (29.5)	122 (35.1)	25 (16.8)	
Moderately	221 (44.5)	148 (42.5)	73 (49.0)	
Poorly	129 (26.0)	78 (22.4)	51 (34.2)	
Surgical approach, no. (%)				0.791
VATS	278 (55.9)	196 (56.3)	82 (55.0)	
Open thoracotomy	219 (44.1)	152 (43.7)	67 (45.0)	
Type of resection, no. (%)				0.167
Sublobar resection	22 (4.4)	12 (3.4)	10 (6.7)	
Lobectomy	475 (95.6)	336 (96.6)	139 (93.3)	
Micropapillary/solid pattern, no. (%)				0.007
Absent	269 (54.1)	202 (58.0)	67 (45.0)	
Non-predominant	177 (35.6)	118 (33.9)	59 (39.6)	
Predominant	51 (10.3)	28 (8.0)	23 (15.4)	

CEA, carcinoembryonic antigen; VATS, video-assisted thoracic surgery.

© Journal of Thoracic Disease. All rights reserved.

Table 2 Univariate and multivariate analysis of recurrence-free survival in all patients

Variable	Univariate analysis			Multivariate analysis		
	HR	95% CI	P	HR	95% CI	P
Age (≥65)	1.396	1.011–1.927	0.043	1.504	1.077–2.099	0.017
Sex (female)	1.160	0.995–1.351	0.057	1.091	0.932–1.276	0.277
Smoking history (yes)	1.170	0.805–1.699	0.411	–	–	–
CEA (≥10 ng/mL)	1.968	1.255–3.084	0.003	1.658	1.048–2.623	0.031
Tumor size (>3 cm)	1.694	1.232–2.330	0.001	1.332	0.951–1.866	0.095
Visceral pleural invasion (present)	0.705	0.464–1.072	0.102	–	–	–
Surgical approach (open thoracotomy)	1.004	0.737–1.367	0.982	–	–	–
Type of resection (sublobar resection)	1.809	0.953–3.433	0.07	–	–	–
Adjuvant chemotherapy (yes)	0.790	0.558–1.119	0.185	–	–	–
Micropapillary/solid pattern	–	–	<0.001	–	–	0.001
Absent	1.000	–	–	1.000	–	–
Non-predominant	1.437	1.030–2.006	0.033	1.266	0.898–1.785	0.178
Predominant	2.818	1.792–4.432	<0.001	2.529	1.550–4.126	<0.001
Differentiation grade	–	–	–	–	–	–
Well	1.000	–	–	–	–	–
Moderately	1.267	0.889–1.807	0.191	–	–	–
Poorly	0.786	0.504–1.225	0.288	–	–	–

HR, hazard ratio; CI, confidence interval; CEA, carcinoembryonic antigen.

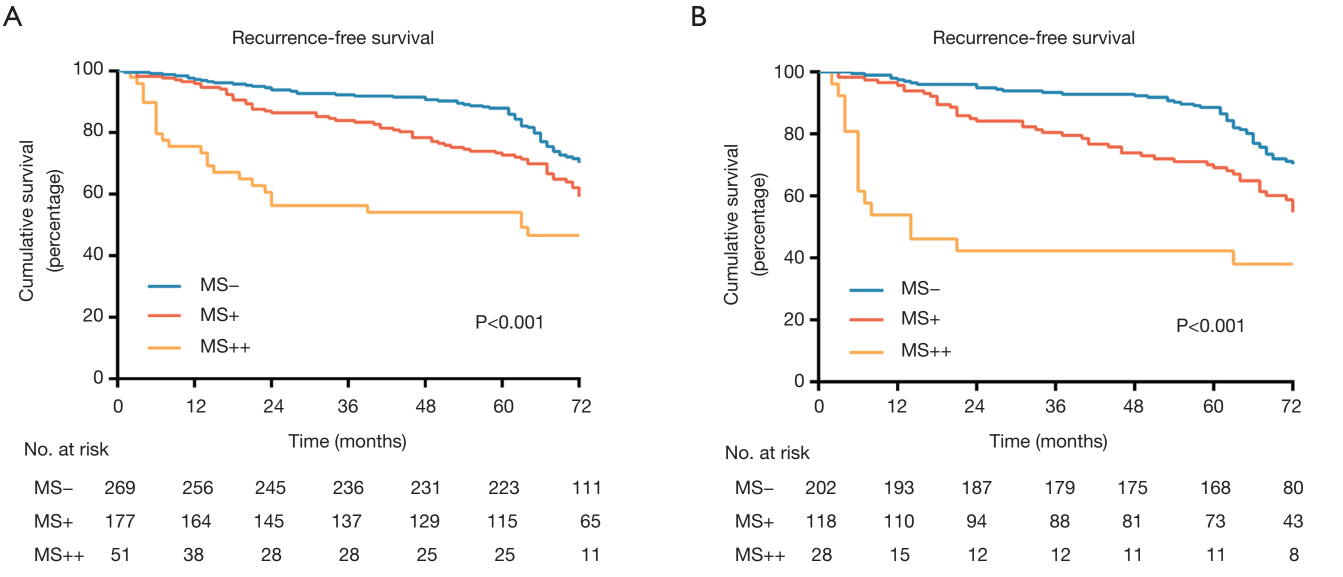

Figure 2 Kaplan-Meier survival curves for recurrence-free survival according to the presence of micropapillary or solid pattern: (A) all patients; (B) patients treated with surgery alone.

© Journal of Thoracic Disease. All rights reserved.

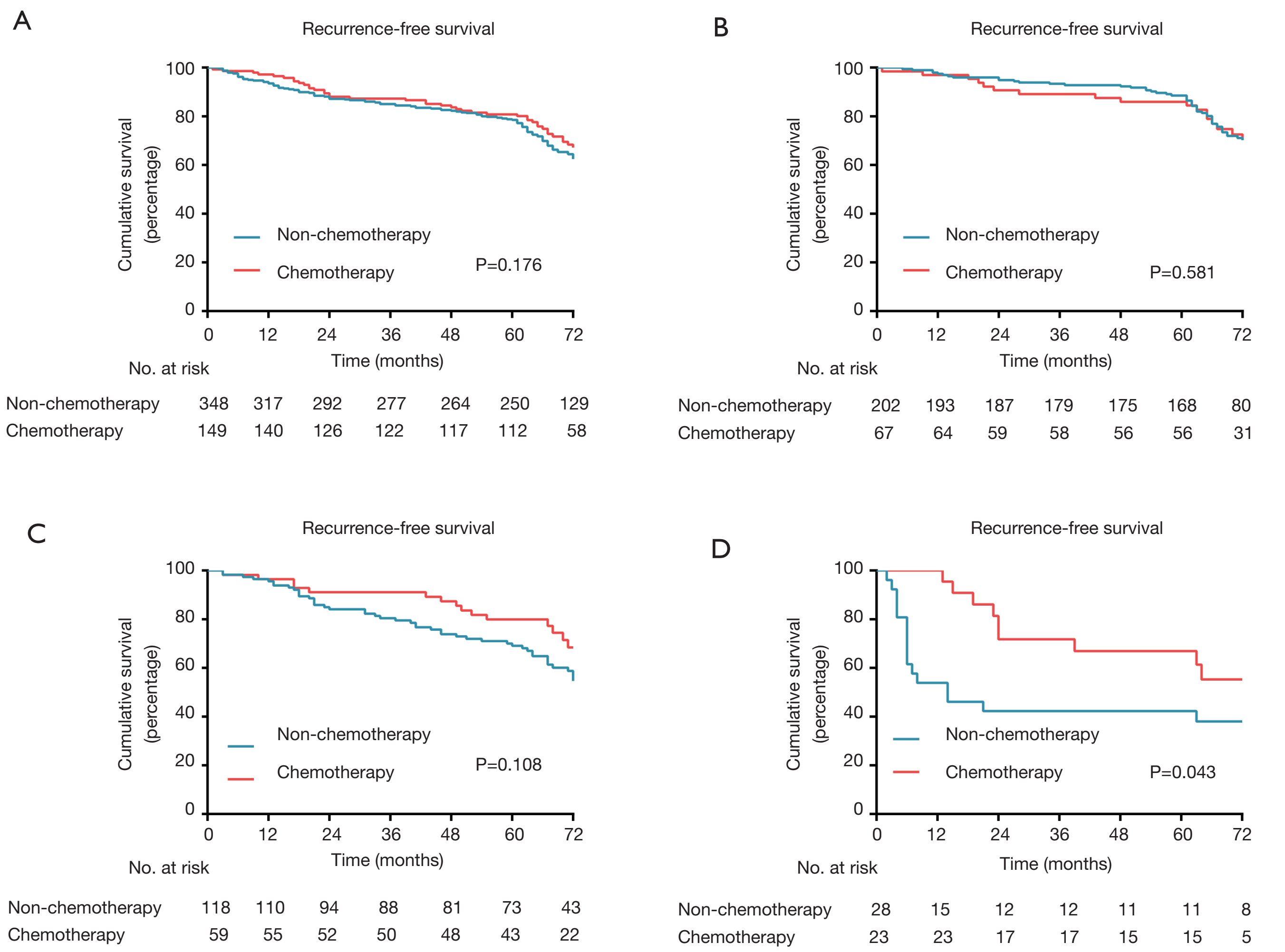

Figure 3 Kaplan-Meier survival curves for recurrence-free survival according to adjuvant chemotherapy: (A) all patients; (B) patients with absent micropapillary or solid pattern adenocarcinoma (MS−); (C) patients with non-predominant micropapillary or solid pattern adenocarcinoma (MS+); (D) patients with predominant micropapillary or solid pattern adenocarcinoma (MS++).

significant benefit in RFS was recognized from ACT (P=0.176; *Figure 3A*). However, in the subgroup analysis based on MS pattern, no RFS benefit from ACT was found in MS− subgroup (P=0.581, *Figure 3B*), a nonsignificant trend toward a RFS benefit from ACT was observed in the MS+ subgroup (P=0.108; *Figure 3C*), and a substantial ACT benefit was identified in the MS++ subgroup (P=0.043; *Figure 3D*). Among the patients with MS++, ACT benefit was significant for RFS (HR, 0.357; 95% CI, 0.152–0.836; P=0.018) and older age (HR, 6.428; 95% CI, 2.495–16.560; P<0.001) was a significant prognostic factor for a worse probability of RFS on univariate and multivariate analysis (*Table 3*). Additionally, we found that a CEA serum level ≥10 ng/mL was associated with ACT benefit (P=0.026; *Figure S1A*) but age ≥65 was not (P=0.281; *Figure S1B*). In subgroup of patients with a CEA serum level ≥10 ng/mL, age and ACT were independent prognosis predictor (*Table S1*).

Discussion

According to National Comprehensive Cancer Network (NCCN) guideline, only patients with high-risk factors including vascular invasion, visceral pleura invasion, tumor size >4 cm, wedge resection, or unknown lymph mode status are recommended receiving ACT. Except for tumors

© Journal of Thoracic Disease. All rights reserved.

Table 3 Univariate and multivariate analysis of recurrence-free survival in 51 patients with predominant micropapillary/solid adenocarcinoma

Variable	Univariate analysis			Multivariate analysis		
	HR	95% CI	P	HR	95% CI	P
Age (≥65)	5.158	2.153–12.358	<0.001	6.428	2.495–16.560	<0.001
Sex (female)	0.977	0.649–1.471	0.912	–	–	–
Smoking history (yes)	0.924	0.386–2.213	0.859	–	–	–
CEA (≥10 ng/mL)	0.921	0.275–3.087	0.894	–	–	–
Tumor size (>3 cm)	0.970	0.442–2.128	0.940	–	–	–
Visceral pleural invasion (present)	0.636	0.264–1.528	0.311	–	–	–
Surgical approach (open thoracotomy)	0.601	0.251–1.443	0.255	–	–	–
Type of resection (sublobar resection)	1.426	0.191–10.620	0.729	–	–	–
Differentiation grade				–	–	–
Well	1.000	–	–	–	–	–
Moderately	0.691	0.286–1.668	0.411	–	–	–
Poorly	1.104	0.383–3.179	0.855	–	–	–
Adjuvant chemotherapy (yes)	0.433	0.191–0.983	0.045	0.357	0.152–0.836	0.018

HR, hazard ratio; CI, confidence interval; CEA, carcinoembryonic antigen.

size >4 cm, there are no prospective randomized clinical trials having validated the correlation between the ACT benefit and these high-risk factors (6). Additionally, with the 8th TNM classification (7), lung carcinoma >4 cm in greatest dimension has changed from stage IB to stage IIA. Therefore, the necessity of ACT in stage IB needs to be re-evaluated. In this study, no survival benefit from ACT was found on the level of the whole population, which confirmed that the ACT should be individualized in patients with IB stage lung adenocarcinoma. Therefore, we need to re-assess the role of these high-risk factors in selecting candidates for ACT in patients with IB lung adenocarcinoma.

Lung adenocarcinoma with MS++ was reported to have a poor prognosis (10,13). Our data validated the prognostic value of MS pattern in stage IB lung adenocarcinoma, and MS++ pattern was correlated with poor prognosis. Among patients with MS++ adenocarcinoma, the RFS was significantly increased for patients receiving ACT, and no benefit from ACT was identified in patients with MS- adenocarcinoma. These results suggested that MS++ pattern was a good indicator for selecting ACT candidates in patients with a stage IB lung adenocarcinoma. Based on a Lung Adjuvant Cisplatin Evaluation study (15), MS++ pattern is a predictive marker for survival benefit from ACT in patients with lung adenocarcinoma. Recently several studies (17-20) also validated the prognostic significance and predictive value of MS++ pattern in lung adenocarcinoma. These results demonstrated the importance of classifying lung adenocarcinomas into each histological subtype in routine practice in order to select high-risk patients with stage IB for postoperative ACT. Therefore, the MS++ pattern should be proposed as a stratification factor in the design of future clinical trials on ACT.

More importantly, our results demonstrated that the tumors with an MS+ pattern showed a significant poorer prognosis compared with those with MS- pattern. Similarly, Yanagawa *et al.* demonstrated that lung adenocarcinomas with MS pattern, regardless of predominance, were associated with a worse prognosis than those with MS– (21,22). Others showed that patients with MS+ adenocarcinoma have a poorer prognosis and higher rate of lymph node metastasis (23-25), which was independently associated with the risk of recurrence in patients treated with limited resection (26). According to these results, a semi-quantitative analysis of the MS pattern is warranted in clinical diagnosis for a more comprehensive description of tumor aggressiveness because of most adenocarcinomas consist of more than one growth pattern. Furthermore, a marginal ACT benefit was also observed

© Journal of Thoracic Disease. All rights reserved.

in MS+ lung adenocarcinoma in our study. Zhao *et al.* previously demonstrated the prognosis of acinar/papillary adenocarcinoma with MS+ (secondary predominant pattern) was similar to those with MS++ (27). That may suggest that patients with MS+ adenocarcinoma need more quantitative and precise evaluation of MS+ pattern for ACT.

We performed a subgroup analysis for evaluating the ACT benefit in patients with other high risk-factors. As for patients with older age, our results showed that patients older than 65 had a significantly worse prognosis but not benefit from ACT. However, because sublobar resection was more likely to be performed for older patients, the prognosis effect of older age might be exaggerated in this study. Considering that older patients with advanced NSCLC are at higher risk for adverse events related to ACT of their disease (28), we hold that diagnosed age ≥65 should not be the indication for ACT in patients with stage IB lung adenocarcinoma. The Serum CEA level was associated with recurrence after complete resection (29) and provided an evidence in monitoring of tumor recurrence of NSCLC (30). In our study, the subgroup analysis showed a significant survival benefit from ACT among patients with higher serum CEA level (≥10 ng/mL) but not in multivariate analysis. Considering CEA as reliable marker of chemotherapy efficacy (31), we believe that higher serum CEA level (≥10 ng/mL) may be an indicator of ACT in patients with stage IB adenocarcinoma, which requires validation in further prospective and randomize control study.

Our study has some limitations. First, this retrospective study was carried out at a single institution with a small sample size, indicating possible selection bias. Additionally, ACT decision and the regimens selection were based on physician preference rather than randomization. Therefore, prospective multi-institutional randomized clinical trials are necessary to validate the results.

In conclusion, we have shown a significant prognosis correlation between RFS and high-risk factors in a cohort of 497 surgically resected lung adenocarcinoma with stage IB. Importantly, we found that the patients with MS++ adenocarcinoma had a better RFS outcomes following ACT, who may be candidates for ACT.

Acknowledgements

Funding: This work supported by technical research and development project of technology benefiting for people of Ningbo, Zhejiang Province, China [grant number 2015C50032], Science and Technology Commission of Shanghai Municipality [grant number 15695840600] and Shanghai Hospital Development Center [grant number 16CR3116B].

Footnote

Conflicts of Interest: The authors have no conflicts of interest to declare.

Ethical Statement: The Shanghai Pulmonary Hospital institutional ethics committee approved this retrospective study (IRB No. K18-101).

References

1. Torre LA, Bray F, Siegel RL, et al. Global cancer statistics, 2012. CA Cancer J Clin 2015;65:87-108.
2. Nakagawa T, Okumura N, Ohata K, et al. Postrecurrence survival in patients with stage I non-small cell lung cancer. Eur J Cardiothorac Surg 2008;34:499-504.
3. Booth CM, Shepherd FA. Adjuvant Chemotherapy for Resected Non-small Cell Lung Cancer. J Thorac Oncol 2006;1:180-7.
4. Booth CM, Shepherd FA, Peng Y, et al. Adoption of adjuvant chemotherapy for non-small-cell lung cancer: a population-based outcomes study. J Clin Oncol 2010;28:3472-8.
5. NSCLC Meta-analyses Collaborative Group, Arriagada R, Auperin A, et al. Adjuvant chemotherapy, with or without postoperative radiotherapy, in operable non-small-cell lung cancer: two meta-analyses of individual patient data. Lancet 2010;375:1267-77.
6. Strauss GM, Herndon JE 2nd, Maddaus MA, et al. Adjuvant paclitaxel plus carboplatin compared with observation in stage IB non-small-cell lung cancer: CALGB 9633 with the Cancer and Leukemia Group B, Radiation Therapy Oncology Group, and North Central Cancer Treatment Group Study Groups. J Clin Oncol 2008;26:5043-51.
7. Goldstraw P, Chansky K, Crowley J, et al. The IASLC Lung Cancer Staging Project: Proposals for Revision of the TNM Stage Groupings in the Forthcoming (Eighth) Edition of the TNM Classification for Lung Cancer. J Thorac Oncol 2016;11:39-51.
8. Travis WD, Brambilla E, Noguchi M, et al. International association for the study of lung cancer/american thoracic society/european respiratory society international

© Journal of Thoracic Disease. All rights reserved.

multidisciplinary classification of lung adenocarcinoma. J Thorac Oncol 2011;6:244-85.

9. Russell PA, Wainer Z, Wright GM, et al. Does lung adenocarcinoma subtype predict patient survival?: A clinicopathologic study based on the new International Association for the Study of Lung Cancer/American Thoracic Society/European Respiratory Society international multidisciplinary lung adenocarcinoma classification. J Thorac Oncol 2011;6:1496-504.
10. Yoshizawa A, Motoi N, Riely GJ, et al. Impact of proposed IASLC/ATS/ERS classification of lung adenocarcinoma: prognostic subgroups and implications for further revision of staging based on analysis of 514 stage I cases. Mod Pathol 2011;24:653-64.
11. Warth A, Muley T, Meister M, et al. The novel histologic International Association for the Study of Lung Cancer/American Thoracic Society/European Respiratory Society classification system of lung adenocarcinoma is a stage-independent predictor of survival. J Clin Oncol 2012;30:1438-46.
12. Song Z, Zhu H, Guo Z, et al. Prognostic value of the IASLC/ATS/ERS classification in stage I lung adenocarcinoma patients--based on a hospital study in China. Eur J Surg Oncol 2013;39:1262-8.
13. Woo T, Okudela K, Mitsui H, et al. Prognostic value of the IASLC/ATS/ERS classification of lung adenocarcinoma in stage I disease of Japanese cases. Pathol Int 2012;62:785-91.
14. Travis WD, Brambilla E, Nicholson AG, et al. The 2015 World Health Organization Classification of Lung Tumors: Impact of Genetic, Clinical and Radiologic Advances Since the 2004 Classification. J Thorac Oncol 2015;10:1243-60.
15. Tsao MS, Marguet S, Le Teuff G, et al. Subtype Classification of Lung Adenocarcinoma Predicts Benefit From Adjuvant Chemotherapy in Patients Undergoing Complete Resection. J Clin Oncol 2015;33:3439-46.
16. Hung JJ, Yeh YC, Jeng WJ, et al. Predictive value of the international association for the study of lung cancer/American Thoracic Society/European Respiratory Society classification of lung adenocarcinoma in tumor recurrence and patient survival. J Clin Oncol 2014;32:2357-64.
17. Hung JJ, Wu YC, Chou TY, et al. Adjuvant Chemotherapy Improves the Probability of Freedom From Recurrence in Patients With Resected Stage IB Lung Adenocarcinoma. Ann Thorac Surg 2016;101:1346-53.
18. Xu CH, Wang W, Wei Y, et al. Prognostic value of the new International Association for the Study of Lung Cancer/American Thoracic Society/European Respiratory Society classification in stage IB lung adenocarcinoma. Eur J Surg Oncol 2015;41:1430-6.
19. Luo J, Huang Q, Wang R, et al. Prognostic and predictive value of the novel classification of lung adenocarcinoma in patients with stage IB. J Cancer Res Clin Oncol 2016;142:2031-40.
20. Qian F, Yang W, Wang R, et al. Prognostic significance and adjuvant chemotherapy survival benefits of a solid or micropapillary pattern in patients with resected stage IB lung adenocarcinoma. J Thorac Cardiovasc Surg 2018;155:1227-1235.e2.
21. Yanagawa N, Shiono S, Abiko M, et al. The Clinical Impact of Solid and Micropapillary Patterns in Resected Lung Adenocarcinoma. J Thorac Oncol 2016;11:1976-83.
22. Suzuki M, Yokose T, Nakayama H. Prognostic contribution of non-predominant solid and micropapillary components in lung adenocarcinomas. J Thorac Dis 2017;9:504-6.
23. Lee G, Lee HY, Jeong JY, et al. Clinical impact of minimal micropapillary pattern in invasive lung adenocarcinoma: prognostic significance and survival outcomes. Am J Surg Pathol 2015;39:660-6.
24. Zhang Y, Wang R, Cai D, et al. A comprehensive investigation of molecular features and prognosis of lung adenocarcinoma with micropapillary component. J Thorac Oncol 2014;9:1772-8.
25. Zhao Y, Wang R, Shen X, et al. Minor Components of Micropapillary and Solid Subtypes in Lung Adenocarcinoma are Predictors of Lymph Node Metastasis and Poor Prognosis. Ann Surg Oncol 2016;23:2099-105.
26. Nitadori J, Bograd AJ, Kadota K, et al. Impact of micropapillary histologic subtype in selecting limited resection vs lobectomy for lung adenocarcinoma of 2cm or smaller. J Natl Cancer Inst 2013;105:1212-20.
27. Zhao ZR, Xi SY, Li W, et al. Prognostic impact of pattern-based grading system by the new IASLC/ATS/ERS classification in Asian patients with stage I lung adenocarcinoma. Lung Cancer 2015;90:604-9.
28. Avery EJ, Kessinger A, Ganti AK. Therapeutic options for elderly patients with advanced non-small cell lung cancer. Cancer Treat Rev 2009;35:340-4.
29. Takahashi Y, Horio H, Hato T, et al. Predictors of post-recurrence survival in patients with non-small-cell lung cancer initially completely resected. Interact Cardiovasc Thorac Surg 2015;21:14-20.
30. Holdenrieder S, Wehnl B, Hettwer K, et al.

© Journal of Thoracic Disease. All rights reserved.

Carcinoembryonic antigen and cytokeratin-19 fragments for assessment of therapy response in non-small cell lung cancer: a systematic review and meta-analysis. Br J Cancer 2017;116:1037-45.
31. Ardizzoni A, Cafferata MA, Tiseo M, et al. Decline in serum carcinoembryonic antigen and cytokeratin 19 fragment during chemotherapy predicts objective response and survival in patients with advanced nonsmall cell lung cancer. Cancer 2006;107:2842-9.

Cite this article as: Ma M, She Y, Ren Y, Dai C, Zhang L, Xie H, Wu C, Yang M, Xie D, Chen C. Micropapillary or solid pattern predicts recurrence free survival benefit from adjuvant chemotherapy in patients with stage IB lung adenocarcinoma. J Thorac Dis 2018;10(9):5384-5393. doi: 10.21037/jtd.2018.08.64

Supplementary

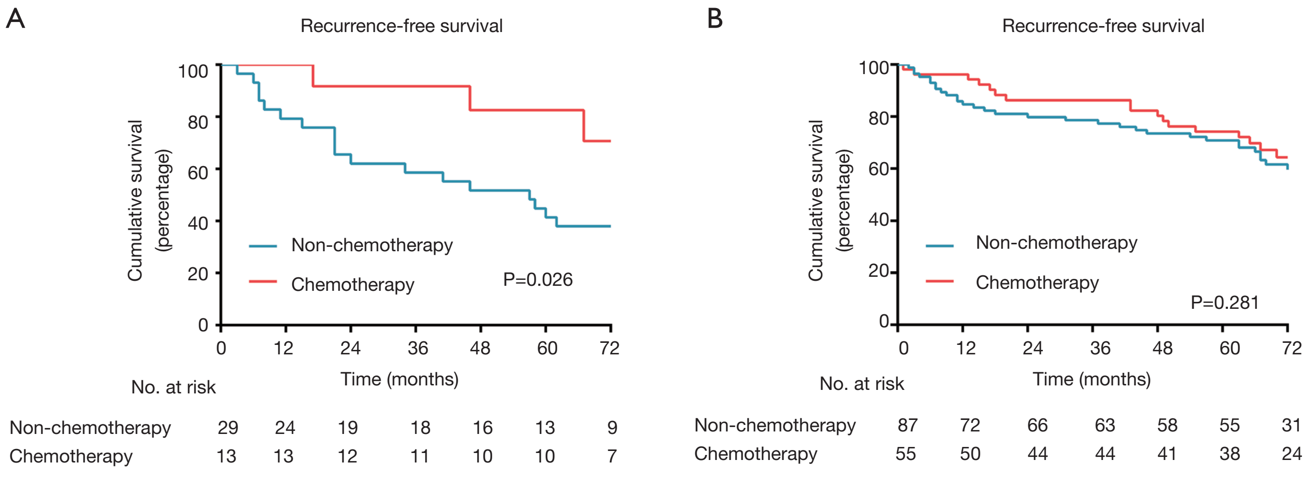

Figure S1 Kaplan-Meier survival curves for recurrence-free survival according to adjuvant chemotherapy. (A) Patients with serum CEV ≥10 ng/mL; (B) patients older than 65 years.

Table S1 Multivariate analysis of recurrence-free survival in 42 patients with CEA ≥10 ng/mL

Variables	HR	95% CI	P
Age (≥65)	5.338	1.611–17.684	0.006
Sex (female)	2.09	0.428–10.236	0.362
Smoking history (yes)	7.634	0.997–58.467	0.054
Tumor size (>3 cm)	1.047	0.313–3.496	0.941
Visceral pleural invasion (present)	1.470	0.30–9.390	0.684
Surgical approach (open thoracotomy)	1.216	0.327–4.512	0.771
Type of resection (sublobar resection)	5.450	0.440–67.511	0.187
Adjuvant chemotherapy	0.167	0.032–0.880	0.035
Micropapillary/solid pattern	–	–	–
Absent	1.000	–	–
Non-predominant	3.240	0.819-12.817	0.094
Predominant	4.152	0.704-24.498	0.116
Differentiation grade	–	–	–
Well	1.000	–	–
Moderately	0.721	0.152–3.414	0.680
Poorly	0.706	0.100–4.982	0.727

HR, hazard ratio; CI, confidence interval; CEA, carcinoembryonic antigen.
