# Supplementary material for: Efficacy and Benefit of Postoperative Chemotherapy in Micropapillray or Solid Predominant Pattern in Stage IB Lung Adenocarcinoma: A Systematic Review and Meta-Analysis
Source: Front Surg. 2021 Dec 21;8:795921. doi: 10.3389/fsurg.2021.795921 (PMC8724201; doi:10.3389/fsurg.2021.795921)
Supplement: Supplementary file 1 [file Data_Sheet_1.ZIP › ╬─╧╫/14.pdf]

J Cancer Res Clin Oncol
DOI 10.1007/s00432-016-2192-6

ORIGINAL ARTICLE – CLINICAL ONCOLOGY

Prognostic and predictive value of the novel classification of lung adenocarcinoma in patients with stage IB

Jizhuang Luo[1] · Qingyuan Huang[1] · Rui Wang[1] · Baohui Han[2] · Jie Zhang[3] · Heng Zhao[1] · Wentao Fang[1] · Qingquan Luo[4] · Jun Yang[1] · Yunhai Yang[1] · Lei Zhu[3] · Tianxiang Chen[1] · Xinghua Cheng[5] · Yiyang Wang[1] · Jiajie Zheng[1] · Han Wu[6] · Weicong Xia[6] · Haiquan Chen[1,5,7,8]

Received: 8 May 2016 / Accepted: 10 June 2016
© Springer-Verlag Berlin Heidelberg 2016

Abstract
Purpose Controversy remains exist for the effect of adjuvant chemotherapy (ACT) among stage IB lung adenocarcinoma patients. This study aimed to investigate the predictive value of the current lung adenocarcinoma classification system on benefit of ACT among patients with stage IB lung adenocarcinoma.
Methods A total of 928 pathological stage IB invasive adenocarcinoma patients with R0 resection were included in this study. Based on the predominant growth pattern present in the tumor, invasive adenocarcinomas with mixed histologic components were classified into five subtypes: lepidic (LEP), acinar (ACN), papillary (PAP), micropapillary (MIP) and solid (SOL). These five histologic subtypes were collapsed into three groups (LEP, ACN/PAP and SOL/MIP). Disease-free survival (DFS) and overall survival (OS) were analyzed to evaluate benefit from ACT in patients with different histologic patterns using the Kaplan–Meier approach and multivariable Cox models.
Results For all stage IB invasive adenocarcinoma patients, SOL/MIP subgroup presented the worst prognosis, and LEP subgroup showed approximately 100 % 5-year survival. ACT was associated with a better DFS (HR, 0.70; 95 % CI 0.51–0.96, $p = .026$) for all stage IB patients. In SOL/MIP subgroup, patients could benefit from ACT for a significant improved DFS (HR, 0.81; 95 % CI 0.49–1.35; $p = .030$), but not for OS (HR, 0.39; 95 % CI 0.12–1.30, $p = .111$). In ACN/PAP subgroup, there was no significant benefit from ACT for both DFS (HR, 0.76; 95 % CI 0.54–1.08, $p = .125$) and OS (HR, 0.81; 95 % CI 0.49–1.35, $p = .421$).
Conclusions SOL/MIP predominant pattern was predictive for ACT benefit for DFS among invasive lung adenocarcinoma patients in stage IB.

Keywords Invasive lung adenocarcinoma · Non-small-cell lung cancer · Stage IB · Adjuvant chemotherapy · Prognosis

Jizhuang Luo, Qingyuan Huang and Rui Wang contributed equally to this work.

✉ Haiquan Chen
hqchen1@yahoo.com

[1] Department of Thoracic Surgery, Shanghai Chest Hospital, Shanghai Jiao Tong University, Shanghai, China

[2] Department of Pulmonary Medicine, Shanghai Chest Hospital, Shanghai Jiao Tong University, Shanghai, China

[3] Department of Pathology, Shanghai Chest Hospital, Shanghai Jiao Tong University, Shanghai, China

[4] Department of Shanghai Lung Tumor Clinic Center, Shanghai Chest Hospital, Shanghai Jiao Tong University, Shanghai, China

[5] Department of Thoracic Surgery, Fudan University Shanghai Cancer Center, 270, Dong-An Road, Shanghai 200032, China

[6] Department of Thoracic Surgery, Shanghai Ruijin Hosptial, School of Medicine, Shanghai Jiao Tong University, Shanghai, China

[7] Institutes of Biomedical Sciences, Fudan University, Shanghai, China

[8] Department of Oncology, Shanghai Medical College, Fudan University, Shanghai, China

Introduction

Lung cancer continues to be a life-threatening disease for the high morbidity and mortality worldwide. Non-small-cell lung cancer (NSCLC) accounts for approximately

Published online: 05 July 2016

80–90 % in all lung cancer histologic types of which up to 40 % are adenocarcinoma (Bueno et al. 2015; Coutinho et al. 2016; Park et al. 2013). The mainstay of treatment for early-stage NSCLC is complete surgical resection with mediastinal lymph node dissection or systematic sampling. For pathologic stage IB NSCLC, however, the 5-year survival is only about 70 % (Goldstraw et al. 2016). The Lung Adjuvant Cisplatin Evaluation (LACE) meta-analysis pooled data from five trials found a 5-year survival benefit of 5.4 % among patients who had received adjuvant cisplatin-based chemotherapy after complete resection (Pignon et al. 2008; Pirker 2014). However, the benefit of adjuvant chemotherapy (ACT) among stage IB patients remains controversial. The National Comprehensive Cancer Network (NCCN) guideline suggested patients in stage IB should consider adjuvant chemotherapy with high-risk factors including tumor size greater than 4 cm, visceral pleura invasion, lymphovascular invasion, poorly differentiated, wedge resection or incomplete lymph node sampling (Nx) (Ettinger et al. 2015). However, this recommendation is category 2B, lacking for high-level evidence and uniform NCCN consensus. Besides, these clinicopathologic risk factors do not predict which patients are likely to benefit from chemotherapy. Investigating circulating molecular predictors may help to identify the appropriate patients for ACT; however, none has yet been sufficiently informative or reproducible for clinical application (Zhu et al. 2009). Excision repair cross-complementation group 1 (ERCC1) protein was once a promising biomarker (Wilcox 2006), but its usefulness was limited for specific antibodies which currently unavailable to detect the uniform functional isoform (Friboulet et al. 2013). Thus, there is an urgent need to stratify patients at risk who might actually benefit from adjuvant chemotherapy.

Histologic examination is a practical and inexpensive method to investigate biological behavior of tumor (Huang et al. 2016). A new classification system for lung adenocarcinoma based on the predominant histologic pattern present in the resected tumor had been proposed by the International Association for the Study of Lung Cancer (IASLC), American Thoracic Society (ATS) and European Respiratory Society (ERS) in 2011 (Travis et al. 2011). According to the predominant growth pattern, invasive adenocarcinoma is divided into 5 major subtypes including lepidic, acinar, papillary, micropapillary and solid as well as the variants subtypes including mucinous adenocarcinoma, colloid, fetal and enteric morphologies. Previous studies have demonstrated the correlation between histologic subtypes with overall survival and disease-free survival in lung adenocarcinoma patients who received ACT. Patients in lepidic subgroup generally have an excellent prognosis, while micropapillary and solid predominant adenocarcinomas have a significantly higher possibility of recurrence than other histologic predominant pattern, even among patients with stage I (Hung et al. 2013; Tsao et al. 2015; Yoshizawa et al. 2011). However, the value of this new classification in predicting survival benefit from ACT among stage IB patients remains unclear. In our study, we collected a large cohort of patients aiming to evaluate the predictive value of the IASLC/ATS/ERS lung adenocarcinoma classification system on the benefit of ACT among stage IB patients.

Methods

Patients and follow-up

This retrospective study was performed with approval from the Shanghai chest hospital institutional review broad. A total of 1064 stage IB invasive adenocarcinoma patients were included in this study. The selection process was displayed in Fig. 1. All patients underwent R0 resection and complete lymph node dissection between January 2009 and March 2015 from Shanghai chest hospital, Shanghai, China. Patients who have multiple primary lung cancer or induction therapies were excluded. Among them, 44 patients were classified as having invasive adenocarcinoma variants, including 39 mucinous, 3 enteric and 2 fetal morphologies. These 44 patients were excluded from analysis for the low incidence.

Head, chest and upper abdomen CT scans, pulmonary function testing were routinely performed pre-operatively in all patients, and they were advised to follow-up regularly in our outpatient department. According to the basic plan of follow-up in our hospital, Chest CT scans and upper abdomen ultrasound examination were performed in every 3 months for the first year after surgery and at 6-month intervals thereafter. Whole-body bone scanning and magnetic resonance imaging (MRI) of the brain were arranged annually or when clinical symptoms occurred during follow-up. For patients who did not follow-up in out hospital, we conducted a telephone follow-up to record the survival status as well as results of their previous examination performed in other hospitals. 92 patients who missed follow-up were excluded. Finally, the remaining 928 patients with complete data were included in this study.

The indication of platinum-based adjuvant chemotherapy for stage IB lung adenocarcinoma patients was patients with high-risk factors including tumor size greater than 4 cm, visceral pleura invasion, lymphovascular invasion, poorly differentiated or wedge resection with patients' agreement. Age, basic diseases and performance status are all be considered. All patients recommended for four cycles chemotherapy unless server side-effect like bone marrow suppression occurred.

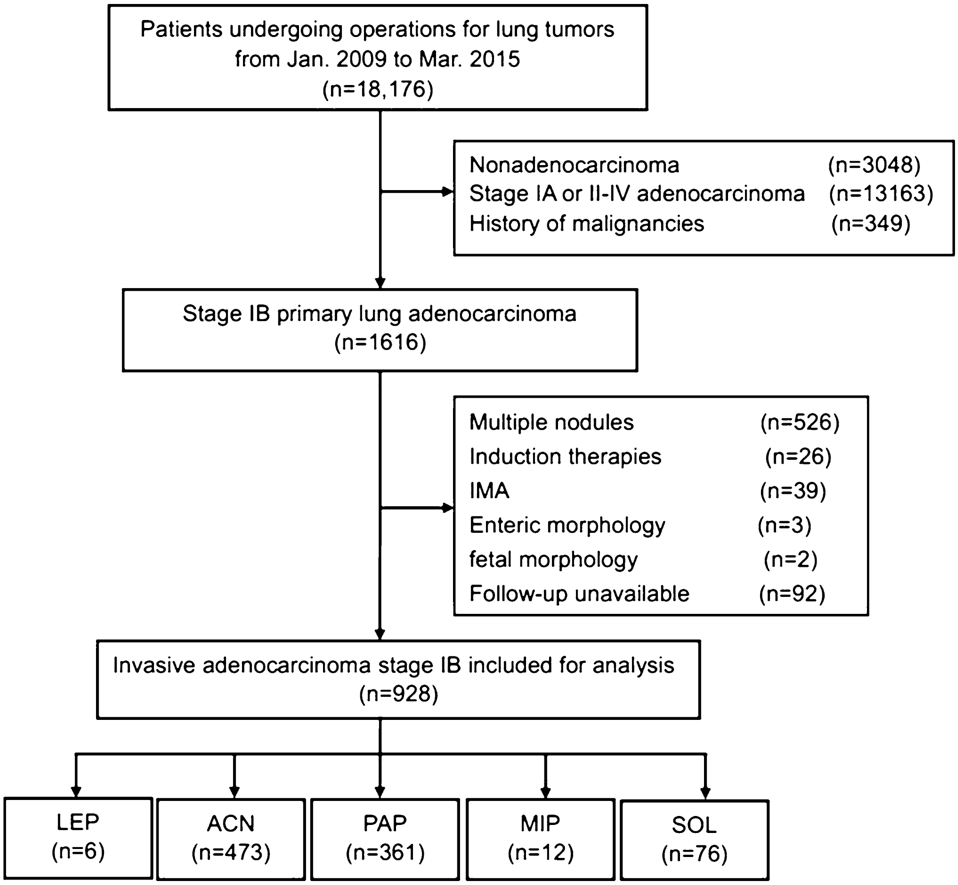

Fig. 1 Selection process of eligible patients

Histopathological reviews

The TNM Staging was based on the 7th edition of the American Joint Committee on Cancer (AJCC) cancer staging manual (Goldstraw et al. 2007). The pathologic features including tumor size, tumor cell type, visceral pleural invasion, lymphovascular invasion and lymph node invasion were obtained from patients' medical records. Based on the predominant growth pattern present in the tumor, invasive adenocarcinomas with mixed histologic patterns were classified into one of the following subtypes: lepidic, acinar, papillary, micropapillary and solid. Two pathologists independently reevaluated the histologic classification of surgical resected specimens which collected before the new IASLC/ATS/ERS classification of lung adenocarcinoma conducted in department of pathology of our hospital.

Statistical methods

The main end point was overall survival (OS), defined as time from surgery to date of death resulting from any cause. The secondary end point was disease-free survival (DFS), defined as time from surgery to date of first event (recurrence or metastasis). Patients with no event were censored at the date of their last follow-up. OS and DFS were obtained from clinical medical records or telephone follow-up. The correlation between the histologic subtypes and covariates was tested using the Cochran–Mantel–Haenszel test. The log-rank test was used to compare the survival curves estimated by the Kaplan–Meier method between histologic subtypes. Multivariable Cox models stratified by trial and adjusted for gender, age, tumor size, histologic predominant pattern, pleural invasion, lymphovascular invasion and type of surgery were used to measure the prognostic value of these subtypes in the observation arm. To evaluate whether specific histologic subtypes could predict survival benefit from chemotherapy, an interaction term between subtype and treatment (chemotherapy vs. observation) was included. The prognostic and predictive effects of histology subtypes according to stage were also assessed as exploratory analyses. Hazard ratios (HRs) and their 95 % CIs were reported. Survival analyses were performed on the completed patient cases. The level of statistical significance was set to .05 (pooled analysis). Statistical analyses were performed using SPSS software (version 19) and GraphPad (Prism 5).

Results

Among the 928 patients, there were 457 (49.2 %) males and 471 (50.8 %) females. Five hundred and nine (54.8 %) patients underwent platinum-based adjuvant chemotherapy, and 419 (45.2 %) patients were not given adjuvant chemotherapy. The number of cases in age <55, 55–70, >70 years old were 230 (24.8 %), 546 (58.8 %) and 152 (16.4 %). Patients with the following subtypes were lepidic 6 (0.6 %), acinar 473 (48.9 %), papillary 361 (38.9 %), micropapillary 12 (1.3 %) and solid 76 (8.2 %), respectively. 87.9 % patients had received lobectomy. Patients without lymphovascular invasion, tumor size less than 4 cm or with pleural invasion accounted for the majority proportion. The general characteristics and comparisons of patients are shown in Table 1.

Prognostic value of subgroups

Among all 928 patients, the media follow-up time was 46.53 months, the number of events for DFS and OS was 156 (16.8 %) and 72 (7.8 %). Recurrence was reported in 28 (31.8 %) patients in the SOL/MIP subgroup and 128 (15.3 %) patients in the ACN/PAP subgroup. Death was reported in 12 (13.6 %) patients in the SOL/MIP subgroup and 60 (7.2 %) patients in the ACN/PAP subgroup. No recurrence or death case occurred in the LEP subgroup. For all patients, the 5-year DFS and OS were 72.6 and 87.1 %, respectively. In univariable analysis, there was significant prognostic difference between three subgroups for DFS ($p < .001$) and OS ($p = .004$) with the SOL/MIP subgroup showing a worse outcome (Fig. 2). LEP predominant pattern was excluded in multivariable analysis for no death

Table 1 Baseline characteristics of patients with stage IB lung adenocarcinoma

Characteristic	Total ($N = 928$)		Observation ($n = 419$)		ACT ($n = 509$)		p*
	No.	(%)	No.	(%)	No.	(%)	
Sex							.54
Male	457	49.2	211	50.4	246	48.3	
Female	471	50.8	208	49.6	263	51.7	
Age, years							<.001
<55	230	24.8	71	16.9	159	24.8	
55–70	546	58.8	235	56.1	311	58.8	
>70	152	16.4	113	27.0	39	16.4	
Tumor size (cm)							.35
≤4	886	95.5	403	96.2	483	94.9	
>4	42	4.5	16	3.8	26	5.1	
Visceral pleural invasion							.07
Yes	831	89.5	366	87.6	465	91.2	
No	97	10.5	52	12.4	45	8.8	
Lymphovascular invasion							.04
Yes	69	7.4	23	5.5	46	9.0	
No	859	92.6	396	94.5	463	91.0	
Type of Surgery							<.001
Lobectomy	816	87.9	349	83.3	467	91.7	
Wedge resection	94	10.1	56	13.4	38	7.5	
Others	18	1.9	14	3.3	4	0.8	
Adenocarcinoma subtype							.45
Lepidic	6	0.6	2	0.5	4	0.8	
Acinar	473	51.0	227	54.2	246	48.3	
Papillary	361	38.9	153	36.5	208	40.9	
Micropapillary	12	1.3	6	1.4	6	1.2	
Solid	76	8.2	31	7.4	45	8.8	

ACT adjuvant chemotherapy

* Chi-square test was calculated from logistic regression model stratified by trail

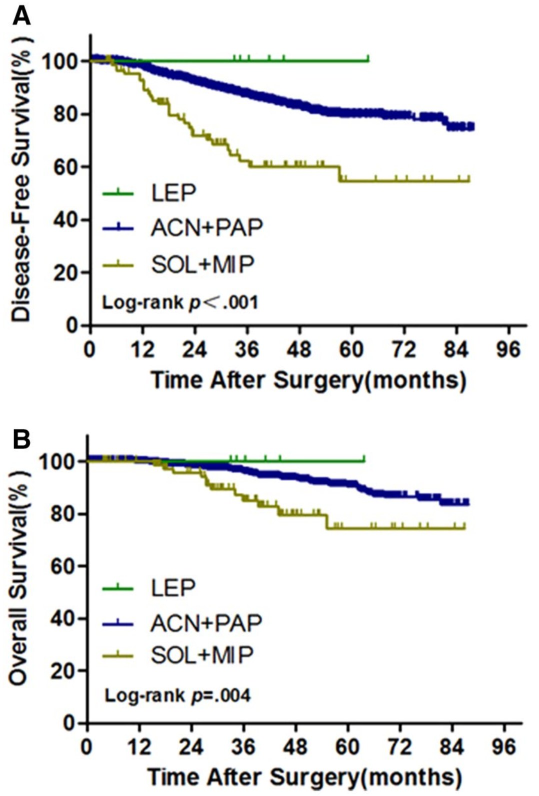

Fig. 2 Survival *curves* for disease-free survival (**a**) and overall survival (**b**) according to three predominant patterns (lepidic [LEP]; acinar [ACN]/papillary [PAP]; micropapillary [MIP]/solid [SOL]). *P* values from log-rank test. Hazard ratios (HRs) estimated through univariable Cox model stratified on trail. *CI* confidence interval

occurred among this group. Multivariable survival analysis showed significant association for the DFS ($p = .000$) and OS ($p = .027$), with a poorer DFS (HR, 2.59; 95 % CI 1.69–3.95) and OS (HR, 2.06; 95 % CI 1.09–3.89; Table 2) for SOL/MIP versus ACN/PAP.

Predictive value of subtypes for ACT benefit

Among all stage IB patients, benefit from adjuvant chemotherapy was significant for DFS (HR, 0.70; 95 % CI 0.51–0.96, $p = .026$) but not for OS (HR, 0.72; 95 % CI 0.45–1.14; $p = .160$; Fig. 3). In order to identify the patients who may actually benefit from ACT, we performed the survival analysis based on the predominant histologic pattern classification. The LEP predominant subgroup was been excluded for the 100 % survival at the end of follow-up. In univariable analysis, there was a significant benefit for ACT for DFS (HR, 0.81; 95 % CI 0.49–1.35; $p = .030$) in the SOL/MIP subgroup, with a non-significant OS (HR 0.39; 95 % CI 0.12–1.30; $p = .111$). No significant benefit was seen from ACT for DFS (HR, 0.76; 95 % CI 0.54–1.08; $p = .125$) and OS (HR, 0.81; 95 % CI 0.49–1.35, $p = .421$; Fig. 3) in ACN/PAP subgroup. Similar results were seen in multivariable analysis. Adjuvant chemotherapy could reduce recurrence for a significant DFS for SOL/MIP (HR, 0.28; 95 % CI 0.11–0.71; $p = .007$; Table 3) but not for ACN/PAP (HR, 0.78; 95 % CI 0.54–1.12; $p = .181$; Table 4) subgroup. No significant difference for OS were seen in both SOL/MIP (HR, 0.33; 95 % CI 0.08–1.32; $p = .117$; Table 3) and ACN/PAP (HR, 0.96; 95 % CI 0.56–1.65; $p = .881$; Table 4) subgroups.

Risk factors for overall survival and disease-free survival

Multivariable Cox models stratified by trial were performed to identify risk factors for disease-free survival and overall survival. We excluded the six patients in LEP subgroup for no event of the main end point occurred among them. For the rest 922 patients, multivariable survival analysis adjusting gender, age, histologic pattern, ACT, tumor size, visceral pleural invasion, lymphovascular invasion and

Table 2 Multivariate analysis of overall survival and disease-free survival

Predictor	DFS			OS		
	HR	95 % CI	*P*	HR	95 % CI	*P*
Sex (female vs. male)	0.73	0.53–1.01	.059	0.43	0.26–0.73	.002
Age (years)			.285			.322
55–70 versus <55	1.38	0.91–2.08	.128	1.48	0.79–2.79	.223
>70 versus <55	1.41	0.84–2.38	.198	1.77	0.83–3.80	.142
Subgroup (SOL/MIP vs. ACN/PAP)	2.59	1.69–3.95	.000	2.06	1.09–3.89	.027
ACT (yes vs. no)	0.67	0.48–0.93	.018	0.80	0.49–1.32	.384
Tumor size (4–5 vs. ≤cm)	3.27	1.88–5.69	.000	3.88	1.79–8.41	.001
Visceral pleural invasion (yes vs. no)	1.15	0.69–1.92	.582	1.64	0.69–3.89	.260
Lymphovascular invasion (yes vs. no)	1.71	1.03–2.84	.040	2.03	0.98–4.21	.057
Wedge resection (yes vs. no)	1.00	0.56–1.81	.991	1.70	0.81–3.57	.163

HR hazard ratio, *CI* confidence interval, *ACT* adjuvant chemotherapy

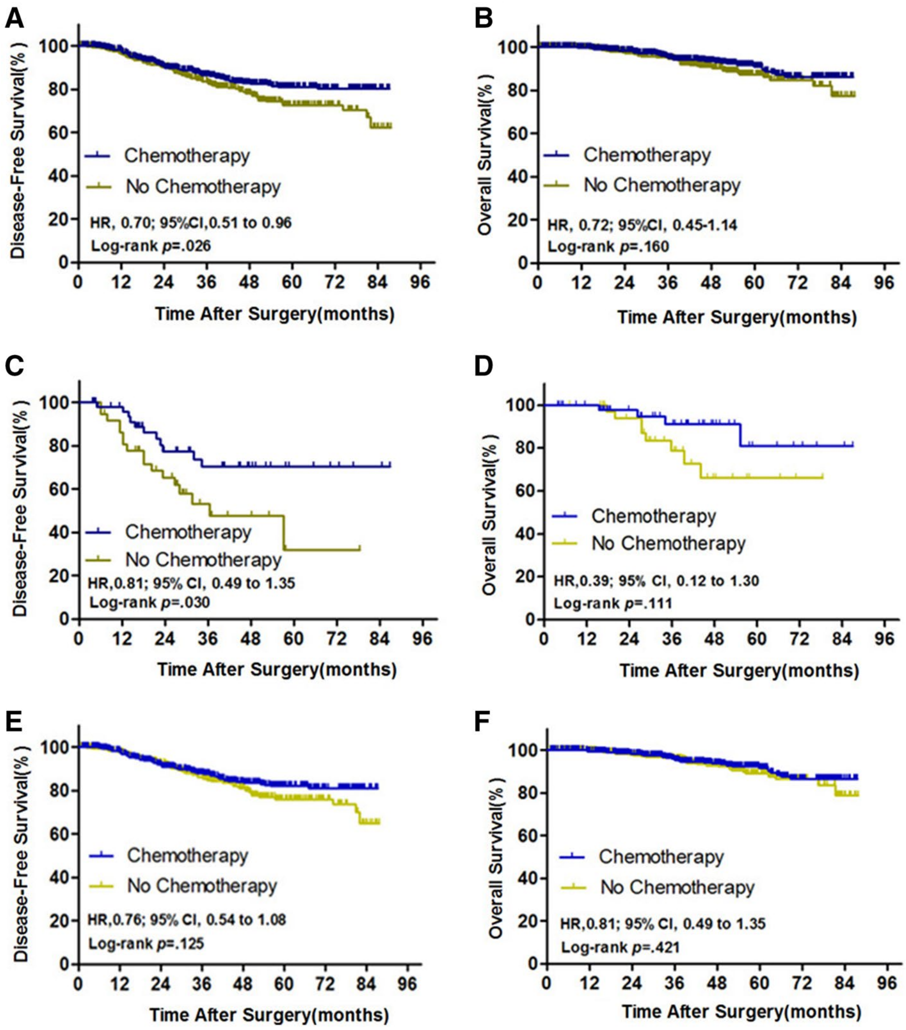

Fig. 3 Survival curves according to treatment arm (ACT vs. observation) in all stage IB patients (**a**, **b**), solid/micropapillary (**c**, **d**) and acinar/papillary (**e**, **f**) subgroups for disease-free survival (**a**, **c**, **e**) and overall survival (**b**, **d**, **f**). *P* values from log-rank test. Hazard ratios (HRs) and 95 % CI estimated through univariable Cox model stratified on trail. *CI* confidence interval

Table 3 Multivariate analysis of disease-free survival and overall survival in MIP/SOL group

Predictor	DFS			OS		
	HR	95 % CI	*P*	HR	95 % CI	*P*
Sex (female vs. male)	0.91	0.38–2.18	.827	0.77	0.19–3.15	.718
Age (years)			.847			.433
55–70 versus <55	1.32	0.48–3.62	.591	3.42	0.39–30.01	.267
>70 versus <55	1.13	0.31–4.11	.857	1.93	0.16–23.20	.603
ACT (yes vs. no)	0.28	0.11–0.71	.007	0.33	0.08–1.32	.117
Tumor size (4–5 vs. ≤4 cm)	7.28	2.24–22.6	.001	7.65	1.72–34.09	.008
Visceral pleural invasion (yes vs. no)	1.45	0.45–4.72	.538	0.41	0.09–1.84	.242
Lymphovascular invasion (yes vs. no)	2.03	0.75–5.53	.166	2.09	0.37–11.83	.403
Wedge resection (yes vs. no)	1.09	0.30–3.93	.902	1.84	0.20–16.77	.587

HR hazard ratio, *CI* confidence interval, *ACT* adjuvant chemotherapy

type of surgery, showing that tumor size greater than 4 cm was an independent risk factor for DFS (HR, 3.27; 95 % CI 1.88–5.69; $p = .000$) and OS (HR, 3.88; 95 % CI 1.79–8.41; $p = .001$). Lymphovascular invasion was another independent risk factor for DFS (HR, 1.71; 95 % CI 1.03–2.84; $p = .040$), but not for OS with a marginally significant p value (HR, 2.03; 95 % CI 0.98–4.21; $p = .057$). Recurrence and death may more likely to occur in male

Table 4 Multivariate analysis of disease-free survival and overall survival in ACN/PAP group

Predictor	DFS			OS		
	HR	95 % CI	*P*	HR	95 % CI	*P*
Sex (female vs. male)	0.69	0.48–0.98	.037	0.40	0.23–0.71	.002
Age (years)			.278			.544
55–70 versus <55	1.44	0.91–2.26	.120	1.37	0.70–2.67	.358
>70 versus <55	1.46	0.81–2.62	.210	1.56	0.68–3.59	.292
ACT (yes vs. no)	0.78	0.54–1.12	.181	0.96	0.56–1.65	.881
Tumor size (4–5 vs. ≤4 cm)	2.55	1.28–5.07	.008	3.37	1.21–8.84	.020
Visceral pleural invasion (yes vs. no)	1.07	0.60–1.89	.828	3.02	0.86–10.62	.085
Lymphovascular invasion (yes vs. no)	1.70	0.93–3.10	.086	2.27	1.02–5.08	.045
Wedge resection (yes vs. no)	1.02	0.52–2.00	.956	2.07	0.92–4.67	.079

HR hazard ratio, *CI* confidence interval, *ACT* adjuvant chemotherapy

Table 5 Multivariate analysis of overall survival and disease-free survival in patients who received ACT

Predictor	DFS			OS		
	HR	95 % CI	*P*	HR	95 % CI	*P*
Sex (female vs. male)	0.91	0.58–1.45	.698	0.57	0.29–1.12	.102
Age (years)			.817			.804
55–70 versus <55	1.17	0.70–1.98	.546	1.28	0.60–2.73	.525
>70 versus <55	1.03	0.41–2.60	.953	1.07	0.28–4.03	.921
Tumor size (4–5 vs. ≤4 cm)	2.77	1.20–6.41	.017	5.83	2.10–16.18	.001
Visceral pleural invasion (yes vs. no)	1.12	0.49–2.56	.794	2.46	0.55–11.09	.241
Lymphovascular invasion (yes vs. no)	2.24	1.19–4.22	.012	2.31	0.87–6.12	.093
Wedge resection (yes vs. no)	1.98	0.78–5.07	.152	1.97	0.45–8.66	.369

HR hazard ratio, *CI* confidence interval, *ACT* adjuvant chemotherapy

for a marginally significant DFS (HR, 0.73; 95 % CI 0.53–1.01; $p = .059$) and a significant OS (HR, 0.43; 95 % CI 0.26–0.73; $p = .002$; Table 2). To evaluate the prognostic value of clinicopathologic characteristics between different histologic patterns, we next performed multivariable survival analysis in SOL/MIP and ACN/PAP subgroups. In SOL/MIP subgroup, only tumor size greater than 4 cm was an independent risk factor for DFS (HR, 7.28; 95 % CI 2.24–22.63; $p = .001$) and OS (HR, 7.65; 95 % CI 1.72–34.09; $p = .008$; Table 3). In ACN/PAP subgroup, tumor size greater than 4 cm was also an independent risk factor for DFS (HR, 2.55; 95 % CI 1.28–5.07; $p = .008$) and OS (HR, 3.37; 95 % CI 1.21–8.84; $p = .020$), and male was another risk factors for DFS (HR, 0.69; 95 % CI 0.48–0.98; $p = .037$) and OS (HR, 0.40; 95 % CI 0.23–0.71; $p = .002$). Additionally, a significant OS (HR, 2.27; 95 % CI 1.02–5.08; $p = .045$) for lymphovascular invasion was seen in this subgroup, with a marginally significant DFS (HR, 1.70; 95 % CI 0.93–3.10; $p = .086$).

We next performed the multivariate analysis for overall survival and disease-free survival in patients who received ACT to study the impact of clinical parameters on outcome with ACT. Multivariate analysis adjusting gender, age, tumor size, visceral pleural invasion, lymphovascular invasion and type of surgery, showing that tumor size greater than 4 cm was an independent risk factor for DFS (HR, 2.77; 95 % CI 1.20–6.41; $p = .017$) and OS (HR, 5.83; 95 % CI 2.10–16.18; $p = .001$). Lymphovascular invasion was another independent risk factor for DFS (HR, 2.24; 95 % CI 1.19–4.22; $p = .012$), but not for OS with a marginally significant p value (HR, 2.31; 95 % CI 0.87–6.12; $p = .093$). These results demonstrated that tumor size greater than 4 cm and lymphovascular invasion still predictive a poor survival among patients who had received ACT (Table 5).

Discussion

To our knowledge, this study was the largest cohort analyzing the prognostic and predictive value of the new lung cancer classification proposed by IASLC/ATS/ERS in 2011 among patients with stage IB lung invasive adenocarcinoma. SOL/MIP predominant pattern was associated with unfavorable prognosis for higher recurrence and death possibility, in accordance with previous studies (Hung et al.

2013; Li et al. 2009; Russell and Wright 2016; Xu et al. 2015; Yanagawa et al. 2013; Yoshizawa et al. 2011; Zhang et al. 2013). Patients with SOL/MIP predominant pattern could benefit from adjuvant chemotherapy for a better DFS after complete resection. In Warth et al's. (2012) report, PAP, MIP and SOL predominant adenocarcinoma composing high-risk grade for the similar survival rate. However, our and other's (Tsao et al. 2015) studies find that no significant difference for survival between PAP and ACN predominant pattern and they were placed in the intermediated survival group. Morphology diversity in papillary predominant group may account for these differences.

Controversy exists among stage IB patients concerning the effect of adjuvant chemotherapy. Currently, the indications which recommended by NCCN guideline for adjuvant chemotherapy including tumor size greater than 4 cm, visceral pleura invasion, lymphovascular invasion, poorly differentiated or wedge resection. However, those indications for stage IB NSCLC patients came from lower-level evidences. A small cohort study (Bueno et al. 2015; Coutinho et al. 2016) found no clear benefit for the NCCN's high-risk patients for OS and DFS. A recent meta-analysis (Shim et al. 2015) included 16 randomized trials indicated that adjuvant chemotherapy was beneficial for OS and DFS in patients with stage IB NSCLC, but the conclusion was not definitive when considering the overall heterogeneity of OS and DFS. In our study, we found that ACT has positive effect on reduce recurrence, with a 8.4 % improvement of 5-year disease-free survival, but there was no advantage in prolonging overall survival.

Our understanding of lung adenocarcinoma has evolved dramatically in the past decade. These are now recognized as a heterogeneous group of tumors with indolent and quite aggressive subtypes, even in the same tumor-node-metastasis group (Donington 2016). The novel IASLC/ATS/ERS classification categorizes adenocarcinoma as different histologic subtypes, which could effectively distinguish their prognosis. The predictive value of histologic subtypes for benefit from ACT in early-stage NSCLC patients has yet to be established. It was demonstrated that advanced high-grade adenocarcinoma had a higher response rate to first-line platinum-based chemotherapy (Campos-Parra et al. 2014). The LACE-Bio stud (Tsao et al. 2015) demonstrated that the benefit of ACT on DFS was significantly different in ACN/PAP and SOL/MIP subgroup, with the latter showing benefit. However, their research included patients with stage I, II and III, and stage II and III patients accounted for 46 %, among whom the effects of adjuvant chemotherapy had been confirmed by multicenter randomized trials. These important findings shed new light on selecting appropriate candidates for ACT among stage IB patients. Our research revealed that ACT could significantly improve DFS among stage IB patients with SOL/MIP predominant pattern. The effects of ACT were not observed in ACN/PAP subgroup. One possible explanation was the SOL/MIP predominant subgroup presented a higher response rate to platinum-based first-line chemotherapy compared with the LEP and ACN/PAP subgroups (Campos-Parra et al. 2014; Takahashi et al. 2016).

In the present study, the improvement in OS of ACT among patients with SOL/MIP predominant pattern was not statistically significant, despite the benefit on DFS. One possible reason is the difference regimens and response in post-recurrence treatments. SOL pattern had significantly lower frequency of EGFR mutations (Yoshizawa et al. 2013), and subsequently less opportunity of tyrosine kinase inhibitors (TKIs) treatments, which were superior to chemotherapy. Moreover, our (Zhang et al. 2014) and others' (Yoshida et al. 2013) previous studies indicated that SOL predominant histology was a negative predictor of response to EGFR TKIs in EGFR-mutated lung adenocarcinoma patients compared to other histologic subtypes.

There are several limitations that should be taken into consideration. First, this retrospective study used a homogeneous cohort, the distribution of genetic mutation and clinicopathologic characteristics might be different in other countries, inherent bias is inevitable. Additionally, the patients' chemotherapy regimens were not the same. The basis chemotherapy regimens were platinum-based combination regimen of two drugs, while the platinum drugs including cisplatin and carboplatin, others drug including gemcitabine, paclitaxel, vinorelbine or pemetrexed. Not all patients in our cohort had completed the 4 cycle chemotherapy for the toxicity of chemotherapy. Thirdly, some clinical parameters like smoking history and *EGFR* mutation were not evaluated in the multivariate and univariate analysis due to incomplete records in our database, and this may led to selection bias. Lastly, our database did not collect information of post-recurrence treatments, which may introduce bias to overall survival, as above mentioned.

In summary, our study showed that the classification based on histologic predominant pattern could effectively distinguish the survival of patients with stage IB lung adenocarcinoma. Patients with SOL/MIP predominant pattern could predict a different disease-free survival benefit from ACT. This findings help to guide the post-operative management of stage IB invasive lung adenocarcinoma patients.

Authors' contribution Dr Haiquan Chen is the guarantor of the manuscript. Dr Jizhuang Luo contributed to conception and study design, acquisition and analysis of data, and writing and revision of the manuscript. Dr Qingyuan Huang contributed to conception and study design, acquisition and analysis of data, and writing and revision of the manuscript. Dr Rui. Wang contributed to conception and study design, acquisition and analysis of data, and writing and revision of the manuscript. Dr Baohui Han contributed to conception and study design, acquisition and analysis of data, and revision of the manuscript. Dr Jie Zhang contributed to acquisition of data. Dr Heng

Zhao contributed to acquisition of data and revision of the manuscript. Dr Wentao Fang contributed to analysis of data and revision of the manuscript. Dr Qingqian Luo contributed to analysis of data and revision of the manuscript. Dr Jun Yang contributed to acquisition of data and revision of the manuscript. Dr Yunhai Yang contributed to acquisition of data and revision of the manuscript. Dr Lei Zhu contributed to analysis of data. Dr Tianxiang Chen contributed to acquisition of data and revision of the manuscript. Dr Xinghua Cheng contributed to acquisition of data and revision of the manuscript. Dr Yiyang Wang contributed to acquisition of data and revision of the manuscript. Dr Jiajie Zheng contributed to analysis of data and revision of the manuscript. Dr Han Wu contributed to acquisition of data and revision of the manuscript. Dr Weicong Xia contributed to analysis of data and revision of the manuscript. Dr Haiquan Chen contributed to conception and study design, analysis of data and review and revision of the manuscript.

Funding This work was funded by National Natural Science Foundation of China (81330056, 81401886, 81401891, 81422029 and 81372525) and Shen-kang Center Project (SKMB1201).

Compliance with ethical standards

Conflict of interest The authors declare that they have no conflicts of interest.

Ethical approval All procedures performed in studies involving human participants were in accordance with the ethical standards of the institutional and/or national research committee and with the 1964 Helsinki Declaration and its later amendments or comparable ethical standards.

Informed consent Written informed consent was obtained from each patient to allow their biological samples to be genetically analyzed.

References

Bueno R et al (2015) Validation of a molecular and pathological model for five-year mortality risk in patients with early stage lung adenocarcinoma. J Thorac Oncol 10:67–73. doi:10.1097/JTO.0000000000000365

Campos-Parra AD, Aviles A, Contreras-Reyes S, Rojas-Marin CE, Sanchez-Reyes R, Borbolla-Escoboza RJ, Arrieta O (2014) Relevance of the novel IASLC/ATS/ERS classification of lung adenocarcinoma in advanced disease. The European Respiratory Journal 43:1439–1447. doi:10.1183/09031936.00138813

Coutinho D, Goncalves A, Antunes A, Campainha S, Miranda J, Barroso A (2016) Adjuvant chemotherapy in stage IB non-small cell lung carcinoma: a survival analysis. Revista portuguesa de pneumologia 22:123–125. doi:10.1016/j.rppnen.2015.09.005

Donington JS (2016) An additional step toward personalization of surgical care for early-stage non-small-cell lung cancer. J Clin Oncol 34:295–296. doi:10.1200/JCO.2015.64.7578

Ettinger DS et al (2015) Non-small cell lung cancer, version 6.2015. J Natl Compr Cancer Netw JNCCN 13:515–524

Friboulet L et al (2013) ERCC1 isoform expression and DNA repair in non-small-cell lung cancer. New Engl J Med 368:1101–1110. doi:10.1056/NEJMoa1214271

Goldstraw P et al (2007) The IASLC lung cancer staging project: proposals for the revision of the TNM stage groupings in the forthcoming (seventh) edition of the TNM classification of malignant tumours. J Thorac Oncol 2:706–714. doi:10.1097/JTO.0b013e31812f3c1a

Goldstraw P et al (2016) The IASLC lung cancer staging project: proposals for revision of the TNM stage groupings in the forthcoming (eighth) edition of the TNM classification for lung cancer. J Thorac Oncol 11:39–51. doi:10.1016/j.jtho.2015.09.009

Huang Q et al (2016) Identification and validation of lymphovascular invasion as a prognostic and staging factor in node-negative esophageal squamous cell carcinoma. J Thorac Oncol 11:583–592. doi:10.1016/j.jtho.2015.12.109

Hung JJ, Jeng WJ, Chou TY, Hsu WH, Wu KJ, Huang BS, Wu YC (2013) Prognostic value of the new International Association for the Study of Lung Cancer/American Thoracic Society/European Respiratory Society lung adenocarcinoma classification on death and recurrence in completely resected stage I lung adenocarcinoma. Ann Surg 258:1079–1086. doi:10.1097/SLA.0b013e31828920c0

Li Z et al (2009) Analysis of the T descriptors and other prognosis factors in pathologic stage I non-small cell lung cancer in China. J Thorac Oncol 4:702–709. doi:10.1097/JTO.0b013e3181a5269d

Park SY et al (2013) Efficacy of platinum-based adjuvant chemotherapy in T2aN0 stage IB non-small cell lung cancer. J Cardiothorac Surg 8:151. doi:10.1186/1749-8090-8-151

Pignon JP et al (2008) Lung adjuvant cisplatin evaluation: a pooled analysis by the LACE Collaborative Group. J Clin Oncol 26:3552–3559. doi:10.1200/JCO.2007.13.9030

Pirker R (2014) Adjuvant chemotherapy in patients with completely resected non-small cell lung cancer. Transl Lung Cancer Res 3:305–310. doi:10.3978/j.issn.2218-6751.2014.09.13

Russell PA, Wright GM (2016) Predominant histologic subtype in lung adenocarcinoma predicts benefit from adjuvant chemotherapy in completely resected patients: discovery of a holy grail? Ann Transl Med 4:16. doi:10.3978/j.issn.2305-5839.2015.10.21

Shim HS et al (2015) Unique Genetic and Survival Characteristics of Invasive Mucinous Adenocarcinoma of the Lung. J Thorac Oncol 10:1156–1162. doi:10.1097/JTO.0000000000000579

Takahashi Y, Eguchi T, Bains S, Adusumilli PS (2016) Significance of IASLC/ATS/ERS classification for early-stage lung adenocarcinoma patients in predicting benefit from adjuvant chemotherapy. Ann Transl Med 4:66. doi:10.3978/j.issn.2305-5839.2015.10.40

Travis WD et al (2011) International association for the study of lung cancer/american thoracic society/european respiratory society international multidisciplinary classification of lung adenocarcinoma. J Thorac Oncol 6:244–285. doi:10.1097/JTO.0b013e318206a221

Tsao MS et al (2015) Subtype classification of lung adenocarcinoma predicts benefit from adjuvant chemotherapy in patients undergoing complete resection. J Clin Oncol 33:3439–3446. doi:10.1200/JCO.2014.58.8335

Warth A et al (2012) The novel histologic International Association for the Study of Lung Cancer/American Thoracic Society/European Respiratory Society classification system of lung adenocarcinoma is a stage-independent predictor of survival. J Clin Oncol 30:1438–1446. doi:10.1200/JCO.2011.37.2185

Wilcox JE (2006) DNA repair by ERCC1 in non-small-cell lung cancer. New Engl J Med 355:2590 **(author reply 2591)**

Xu CH et al (2015) Prognostic value of the new International Association for the Study of Lung Cancer/American Thoracic Society/European Respiratory Society classification in stage IB lung adenocarcinoma. Eur J Surg Oncol 41:1430–1436. doi:10.1016/j.ejso.2015.06.004

Yanagawa N, Shiono S, Abiko M, Ogata SY, Sato T, Tamura G (2013) New IASLC/ATS/ERS classification and invasive tumor size are predictive of disease recurrence in stage I lung adenocarcinoma. J Thorac Oncol 8:612–618. doi:10.1097/JTO.0b013e318287c3eb

Yoshida T et al (2013) Solid predominant histology predicts EGFR tyrosine kinase inhibitor response in patients with EGFR

mutation-positive lung adenocarcinoma. J Cancer Res Clin Oncol 139:1691–1700. doi:10.1007/s00432-013-1495-0

Yoshizawa A et al (2011) Impact of proposed IASLC/ATS/ERS classification of lung adenocarcinoma: prognostic subgroups and implications for further revision of staging based on analysis of 514 stage I cases. Mod Pathol 24:653–664. doi:10.1038/modpathol.2010.232

Yoshizawa A et al (2013) Validation of the IASLC/ATS/ERS lung adenocarcinoma classification for prognosis and association with EGFR and KRAS gene mutations: analysis of 440 Japanese patients. J Thorac Oncol 8:52–61. doi:10.1097/JTO.0b013e3182769aa8

Zhang J, Wu J, Tan Q, Zhu L, Gao W (2013) Why do pathological stage IA lung adenocarcinomas vary from prognosis?: a clinicopathologic study of 176 patients with pathological stage IA lung adenocarcinoma based on the IASLC/ATS/ERS classification. J Thorac Oncol 8:1196–1202. doi:10.1097/JTO.0b013e31829f09a7

Zhang Y et al (2014) The prognostic and predictive value of solid subtype in invasive lung adenocarcinoma. Sci Rep 4:7163. doi:10.1038/srep07163

Zhu ZH et al (2009) Three immunomarker support vector machines-based prognostic classifiers for stage IB non-small-cell lung cancer. J Clin Oncol 27:1091–1099. doi:10.1200/JCO.2008.16.6991
