# Supplementary material for: Efficacy and Benefit of Postoperative Chemotherapy in Micropapillray or Solid Predominant Pattern in Stage IB Lung Adenocarcinoma: A Systematic Review and Meta-Analysis
Source: Front Surg. 2021 Dec 21;8:795921. doi: 10.3389/fsurg.2021.795921 (PMC8724201; doi:10.3389/fsurg.2021.795921)
Supplement: Supplementary file 1 [file Data_Sheet_1.ZIP › ╬─╧╫/250.pdf]

# Adjuvant Chemotherapy Improves the Probability of Freedom From Recurrence in Patients With Resected Stage IB Lung Adenocarcinoma

Jung-Jyh Hung, MD, PhD,* Yu-Chung Wu, MD, Teh-Ying Chou, MD, PhD, Wen-Juei Jeng, MD, Yi-Chen Yeh, MD, and Wen-Hu Hsu, MD*

Division of Thoracic Surgery, Department of Surgery, Taipei Veterans General Hospital and School of Medicine, National Yang-Ming University, Taipei; Department of Pathology and Laboratory Medicine, Taipei Veterans General Hospital, Taipei; Institute of Clinical Medicine, National Yang-Ming University, Taipei; and Department of Internal Medicine, Chang Gung Memorial Hospital and School of Medicine, Chang Gung University, Taipei, Taiwan

***Background.*** **The benefit of adjuvant chemotherapy remains controversial for patients with stage IB non–small-cell lung cancer (NSCLC). This study investigated the effect of adjuvant chemotherapy and the predictors of benefit from adjuvant chemotherapy in patients with stage IB lung adenocarcinoma.**

***Methods.*** **A total of 243 patients with completely resected pathologic stage IB lung adenocarcinoma were included in the study. Predictors of the benefits of improved overall survival (OS) or probability of freedom from recurrence (FFR) from platinum-based adjuvant chemotherapy in patients with resected stage IB lung adenocarcinoma were investigated.**

***Results.*** **Among the 243 patients, 70 (28.8%) had received platinum-based doublet adjuvant chemotherapy. A micropapillary/solid–predominant pattern (versus an acinar/papillary–predominant pattern) was a significantly worse prognostic factor for probability of FFR ($p = 0.033$). Although adjuvant chemotherapy (versus surgical intervention alone) was not a significant prognostic factor for OS ($p = 0.303$), it was a significant prognostic factor for a better probability of FFR ($p = 0.029$) on multivariate analysis. In propensity-score–matched pairs, there was no significant difference in OS between patients who received adjuvant chemotherapy and those who did not ($p = 0.386$). Patients who received adjuvant chemotherapy had a significantly better probability of FFR than those who did not ($p = 0.043$). For patients with a predominantly micropapillary/solid pattern, adjuvant chemotherapy ($p = 0.033$) was a significant prognostic factor for a better probability of FFR on multivariate analysis.**

***Conclusions.*** **Adjuvant chemotherapy is a favorable prognostic factor for the probability of FFR in patients with stage IB lung adenocarcinoma, particularly in those with a micropapillary/solid–predominant pattern.**

**(Ann Thorac Surg 2016;■:■–■)**
**© 2016 by The Society of Thoracic Surgeons**

Lung cancer is the leading cause of cancer death worldwide [1]. Surgical resection is the treatment of choice for early-stage non–small cell lung cancer (NSCLC) [2, 3]. Tumor recurrence is the major cause of treatment failure after resection [4, 5]. Several meta-analyses and randomized controlled trials have reported that adjuvant chemotherapy substantially improved survival in patients with resected NSCLC [6–8]. However, the benefit of adjuvant chemotherapy remains controversial for patients with resected stage IB NSCLC [9].

In 2011, the International Association for the Study of Lung Cancer (IASLC), The American Thoracic Society (ATS), and the European Respiratory Society (ERS) proposed a new classification system for lung adenocarcinoma [10]. Several studies have reported the impact of the new classification on death and recurrence [11–17]. In our previous reports [18, 19], we have shown that micropapillary- and solid-predominant subtypes were significant prognostic factors for death and recurrence [18, 19]. However, the predictive value of the new classification for benefit from adjuvant chemotherapy remains unknown. The present study aimed to determine whether the IASLC/ATS/ERS classification of lung adenocarcinoma is predictive for a benefit of adjuvant chemotherapy in patients with completely resected stage IB lung adenocarcinoma.

## Patients and Methods

### *Patients*

This study was approved by the Institutional Review Board of Taipei Veterans General Hospital. From January

Accepted for publication Oct 26, 2015.

*Drs Hung and Hsu contributed equally to this work.

Address correspondence to Dr Hsu, Division of Thoracic Surgery, Department of Surgery, Taipei Veterans General Hospital, No. 201, Section 2, Shih-Pai Rd, Taipei 112, Taiwan; email: whhsu@vghtpe.gov.tw.

The Appendix can be viewed in the online version of this article [http://dx.doi.org/10.1016/j.athoracsur.2015.10.075] on http://www.annalsthoracicsurgery.org.

© 2016 by The Society of Thoracic Surgeons
Published by Elsevier

0003-4975/$36.00
http://dx.doi.org/10.1016/j.athoracsur.2015.10.075

ARTICLE IN PRESS



2004 to December 2012, all patients who underwent complete resection for stage IB (T2aN0M0) lung adenocarcinoma at Taipei Veterans General Hospital were retrospectively reviewed. Patients undergoing neoadjuvant chemotherapy were excluded. A total of 359 patients with pathologic stage IB lung adenocarcinoma were identified. Among them, 21 patients were classified as having a predominantly lepidic pattern. These 21 patients were excluded from analysis because of their small number and the fact that none of them had received adjuvant chemotherapy. One hundred thirty-eight (40.8%) of the remaining 338 patients received adjuvant therapy. To demonstrate the factors predicting benefits from adjuvant platinum-based doublet chemotherapy, patients received oral tegafur-uracil (n = 58) and epidermal growth factor receptor [*EGFR*] tyrosine kinase inhibitor (n = 6) for adjuvant therapy were excluded from analysis. The remaining 274 patients underwent operation alone or received platinum-based adjuvant chemotherapy. Thirty-one of the 274 patients underwent sublobar resection and were also excluded. The remaining 243 patients were eligible and were included in the study. The preoperative staging workup—including chest and upper abdomen computed tomographic (CT) scans, bronchoscopic examination, CT scanning or magnetic resonance imaging of the brain, nuclear medicine survey of bone—was done as previously described [18, 19] (see Supplemental Patients for details). All patients underwent complete resection of lung cancer with mediastinal lymph node dissection/sampling as previously described [18, 19].

The indication for platinum-based adjuvant chemotherapy in our institution is pathologic stage II-IV disease after surgical resection. According to our previous report [18] regarding death and recurrence in stage I lung adenocarcinoma, visceral pleural invasion and a micropapillary/solid–predominant pattern were significant predictors for recurrence. Although the use of adjuvant chemotherapy and the regimens used for adjuvant chemotherapy in patients with stage IB disease were not randomized but took place according to physician preference in the current study, patients with a predominantly micropapillary/solid pattern were more likely to be offered adjuvant chemotherapy (Table 1). Four cycles were typically administered in the adjuvant setting.

### *Clinicopathologic Characteristics and Patient Follow-Up*

All resected specimens were formalin fixed and stained with hematoxylin and eosin and were reevaluated microscopically as previously described [19]. The criteria

*Table 1. Relationship Between Adjuvant Chemotherapy (Surgical Intervention Alone or With Adjuvant Chemotherapy) and Clinicopathologic Variables in 243 Patients With Stage IB Lung Adenocarcinoma*

| Variable | All Patients | Surgical Intervention Alone (n = 173) | With Adjuvant Therapy (n = 70) | *p* Value |
|---|---|---|---|---|
| Age, y (mean ± SD) | 63.8 ± 10.3 | 64.9 ± 10.3 | 61.1 ± 10.0 | 0.010 |
| Sex, no. (%) | | | | |
| Male | 126 (51.9) | 99 (57.2) | 27 (38.6) | 0.008 |
| Female | 117 (48.1) | 74 (42.8) | 43 (61.4) | |
| Invasive tumor size, no. (%) | | | | |
| ≤3 cm | 168 (69.1) | 120 (69.4) | 48 (68.6) | 0.904 |
| >3 cm | 75 (30.9) | 53 (30.6) | 22 (31.4) | |
| Visceral pleural invasion, n (%)[a] | | | | |
| Absent | 22 (9.1) | 17 (9.9) | 5 (7.1) | 0.501 |
| Present | 220 (90.5) | 155 (90.1) | 65 (92.9) | |
| Unknown | 1 (0.4) | . . . | . . . | |
| Predominant pattern, n (%) | | | | |
| Acinar | 104 (42.8) | 77 (44.5) | 27 (38.6) | 0.016 |
| Papillary | 76 (31.3) | 59 (34.1) | 17 (24.3) | |
| Micropapillary | 38 (15.6) | 19 (11.0) | 19 (27.1) | |
| Solid | 25 (10.3) | 18 (10.4) | 7 (10.0) | |
| Predominant pattern group, n (%) | | | | |
| Acinar/papillary | 180 (74.1) | 136 (78.6) | 44 (62.9) | 0.011 |
| Micropapillary/solid | 63 (25.9) | 37 (21.4) | 26 (37.1) | |
| $FEV_1$ (% predicted) (mean) | 96.4 | 95.7 | 98.0 | 0.299 |
| DLCO (% predicted) (mean) | 73.2 | 72.9 | 74.0 | 0.627 |
| Comorbidity, n (%) | | | | |
| No | 131 (53.9) | 87 (50.3) | 44 (62.9) | 0.075 |
| Yes | 112 (46.1) | 86 (49.7) | 26 (37.1) | |

[a] Patients with unknown status were excluded from the analysis.

DLCO = diffusing capacity of the lungs for carbon monoxide; $FEV_1$ = forced expiratory volume in 1 second; SD = standard deviation.

of the new IASLC/ATS/ERS classification of lung adenocarcinoma were used for histologic classification [10]. The predominant pattern is defined according to the most dominant pattern. We further examined the prognostic value of the new classification according to the risk model proposed by Yoshizawa and coworkers [12]. They divided the predominant pattern group into low grade (predominantly lepidic, acinar, or papillary pattern) and high grade (predominantly micropapillary or solid pattern).

All patients were followed at our outpatient department every 3 months for the first 2 years after resection and at 6-month intervals thereafter. CT scans of the chest and upper abdomen were performed routinely at every scheduled outpatient department visit for follow-up. Whole-body bone scanning was arranged annually during follow-up. A CT scan or magnetic resonance imaging of the brain was performed when neurologic symptoms occurred or when clinical suspicions were raised. Secondary primary lung cancer was differentiated from recurrent NSCLC according to the criteria proposed by Girard and colleagues [20]. The length of overall survival (OS) is defined as the interval between the date of surgical resection and the date of either death or the last follow-up. Time to recurrence (the period of freedom from recurrence [FFR]) is defined as the interval between the date of surgical resection and the date of the first recurrence or the last follow-up. An observation was censored at the last follow-up session when the patient was alive with recurrence-free status or when the patient had died without recurrence.

### *Statistical Analysis*

To compare between groups with respect to categorical and continuous variables, the $\chi^2$ test and the paired independent sample *t* test were used as appropriate. The OS and probability of FFR were calculated by the Kaplan-Meier method [21]. The log-rank test was used to make group comparisons. Univariate and multivariate analyses were performed by means of the Cox proportional hazards model using IBM SPSS Statistics, version 20 (SPSS, Inc, Chicago, IL). All variables with *p* less than 0.1 in univariate analysis were entered into the multivariate analysis. A backward stepwise regression procedure was used. Statistical significance was defined as *p* less than 0.05.

A propensity-score–matched analysis was performed. Surgical intervention alone versus adjuvant chemotherapy was the indicator (dependent variable), and the covariates were age, sex, invasive tumor size, visceral pleural invasion, predominant histologic pattern, forced expiratory volume in 1 second ($FEV_1$), diffusing capacity of the lungs for carbon monoxide (DLCO), and comorbidity. Each variable was multiplied by a coefficient that was calculated using logistic regression analysis, and the sum of these values was taken as the propensity score for

*Table 2. Univariate and Multivariate Analyses of Overall Survival and Probability of Freedom From Recurrence in All 243 Patients With Stage IB Lung Adenocarcinoma*

| | Univariate Analysis | | | Multivariate Analysis | | |
|---|---|---|---|---|---|---|
| Variable | HR | 95% CI | *p* Value | HR | 95% CI | *p* Value |
| Overall survival | | | | | | |
| Age[a] | 1.058 | 1.018–1.100 | 0.004 | 1.053 | 1.014–1.094 | 0.008 |
| Female sex | 1.126 | 0.566–2.241 | 0.735 | . . . | . . . | |
| Invasive tumor size >3 cm | 2.934 | 1.448–5.948 | 0.003 | 2.717 | 1.339–5.511 | 0.006 |
| Visceral pleural invasion | 0.588 | 0.240–1.441 | 0.246 | . . . | . . . | |
| Predominantly micropapillary/solid pattern | 1.469 | 0.711–3.035 | 0.299 | . . . | . . . | |
| With adjuvant therapy | 0.625 | 0.256–1.528 | 0.303 | . . . | . . . | |
| $FEV_1$ | 0.838 | 0.403–1.743 | 0.637 | . . . | . . . | |
| DLCO | 0.666 | 0.306–1.451 | 0.306 | . . . | . . . | |
| Comorbidity | 1.295 | 0.650–2.579 | 0.463 | . . . | . . . | |
| Probability of freedom from recurrence | | | | | | |
| Age[a] | 1.022 | 0.993–1.052 | 0.141 | . . . | . . . | |
| Female sex | 0.658 | 0.364–1.190 | 0.166 | . . . | . . . | |
| Invasive tumor size >3 cm | 1.807 | 1.010–3.231 | 0.046 | 1.681 | 0.934–3.025 | 0.083 |
| Visceral pleural invasion | 0.819 | 0.323–2.073 | 0.673 | . . . | . . . | |
| Predominantly micropapillary/solid pattern | 1.816 | 1.005–3.280 | 0.048 | 1.925 | 1.056–3.512 | 0.033 |
| With adjuvant therapy | 0.473 | 0.220–1.017 | 0.055 | 0.421 | 0.194–0.914 | 0.029 |
| $FEV_1$ | 0.731 | 0.396–1.350 | 0.317 | . . . | . . . | |
| DLCO | 1.238 | 0.644–2.378 | 0.523 | . . . | . . . | |
| Comorbidity | 1.448 | 0.814–2.577 | 0.208 | . . . | . . . | |

[a] The HR associated with age is that the increase in hazard is associated with a 1-year increase in age.

CI = confidence interval; DLCO = diffusing capacity of the lungs for carbon monoxide; $FEV_1$ = forced expiratory volume in 1 second; HR = hazard ratio.

ARTICLE IN PRESS



individual patients. For matching, surgical intervention alone and adjuvant chemotherapy pairs with an equivalent propensity score were selected in a 2:1 match. Matching was performed manually after calculation of propensity scores by computer. IBM SPSS Statistics, version 20, was used to perform the analyses.

## Results

The median follow-up time for all 243 patients was 45.1 months (range, 4.8–110.0 months). The characteristics of these patients are listed in Table 1. Among these 243 patients, 184 (75.7%) were free of tumor recurrence, 47 (19.4%) experienced recurrence, and 12 (4.9%) had an unknown recurrent status during follow-up. For all patients, the 5-year OS and probability of FFR were 80.6% and 75.2%, respectively. *EGFR* mutation status was known in 50 (20.6%) of the 243 patients. Among the 50 patients with known *EGFR* mutation status, 38 (76.0%) patients had an activated *EFGR* mutation, whereas the other 12 (24.0%) patients did not. Among the 47 patients who experienced recurrence after operation, *EGFR* mutation status was known in 17 (36.2%) patients. Thirteen (76.5%) of the 17 patients had an activated *EGFR* mutation. Eight (61.5%) of the 13 patients received an *EGFR* tyrosine kinase inhibitor after recurrence.

### *Adjuvant Chemotherapy*

Among the 243 patients, 70 (28.8%) had received platinum-based doublet adjuvant chemotherapy (Table 1). The regimens of platinum-based doublet chemotherapy in addition to cisplatin/carboplatin included docetaxel in 58 patients, vinorelbine in 9 patients, and gemcitabine in 3 patients. No patient had received adjuvant radiotherapy. The relationship between the use of adjuvant chemotherapy and clinicopathologic variables is shown in Table 1. The adjuvant chemotherapy group had significantly more patients of younger age ($p = 0.010$), female sex ($p = 0.008$), and micropapillary/solid–predominant pattern ($p = 0.011$).

### *Survival Analysis for All Patients*

First, we performed survival analyses in all 243 patients. A predominantly micropapillary/solid pattern was not a significant prognostic factor for OS ($p = 0.299$) (Table 2). For probability of FFR, a predominantly micropapillary/solid pattern was a significantly worse prognostic factor ($p = 0.033$) on multivariate analysis. Adjuvant chemotherapy (versus surgical intervention alone) was not a significant prognostic factor for OS ($p = 0.303$). Adjuvant chemotherapy (versus surgical intervention alone) was a significant prognostic factor for a better probability of FFR ($p = 0.029$) on multivariate analysis.

### *Propensity-Score–Matched Analysis*

When propensity-score matching was performed and covariates such as age, sex, invasive tumor size, visceral pleural invasion, predominant histologic pattern, $FEV_1$, DLCO, and comorbidity were included, pairs who had surgical intervention alone and pairs who had adjuvant

*Table 3. Propensity-Score–Matched Comparison of Clinicopathologic Variables Between Patients Who Had Adjuvant Therapy and Those Who Had Surgical Intervention Alone*

| Variable | Surgical Intervention Alone (n = 90) | With Adjuvant Therapy (n = 45) | *p* Value |
|---|---|---|---|
| Age, y (mean ± SD) | 62.0 ± 9.9 | 61.1 ± 9.8 | 0.624 |
| Sex, n (%) | | | |
| Male | 40 (44.4) | 21 (46.7) | 0.807 |
| Female | 50 (55.6) | 24 (53.3) | |
| Invasive tumor size, cm (mean ± SD) | 2.5 ± 1.0 | 2.5 ± 0.8 | 0.839 |
| Visceral pleural invasion, n (%) | | | |
| Absent | 6 (6.7) | 4 (8.9) | 0.642 |
| Present | 84 (93.3) | 41 (91.1) | |
| Predominant pattern, n (%) | | | |
| Acinar | 38 (42.1) | 18 (40.0) | 0.953 |
| Papillary | 23 (25.6) | 12 (26.7) | |
| Micropapillary | 15 (16.7) | 9 (20.0) | |
| Solid | 14 (15.6) | 6 (13.3) | |
| Predominant pattern group, n (%) | | | |
| Acinar/papillary | 61 (67.8) | 30 (66.7) | 0.897 |
| Micropapillary/solid | 29 (32.2) | 15 (33.3) | |
| $FEV_1$ (% predicted) (mean) | 96.0 | 97.5 | 0.522 |
| DLCO (% predicted) (mean) | 73.7 | 73.7 | 0.993 |
| Comorbidity, n (%) | | | |
| No | 52 (57.8) | 26 (57.8) | 1.000 |
| Yes | 38 (42.2) | 19 (42.2) | |

DLCO = diffusing capacity of the lungs for carbon monoxide; $FEV_1$ = forced expiratory volume in 1 second; SD = standard deviation.

ARTICLE IN PRESS



chemotherapy were well matched (90 patients had surgical procedures alone and 45 patients had adjuvant chemotherapy) (Table 3). Among propensity-score–matched pairs, there was no significant difference in OS between patients who received adjuvant chemotherapy and those who did not ($p = 0.386$) (Fig 1A). Patients who received adjuvant chemotherapy showed a significantly better probability of FFR than those who did not ($p = 0.043$) (Fig 1B).

### *Survival Analysis for All Patients Stratified by Histologic Predominant Pattern*

For patients with a micropapillary/solid–predominant pattern (n = 63), 37 were treated with surgical intervention

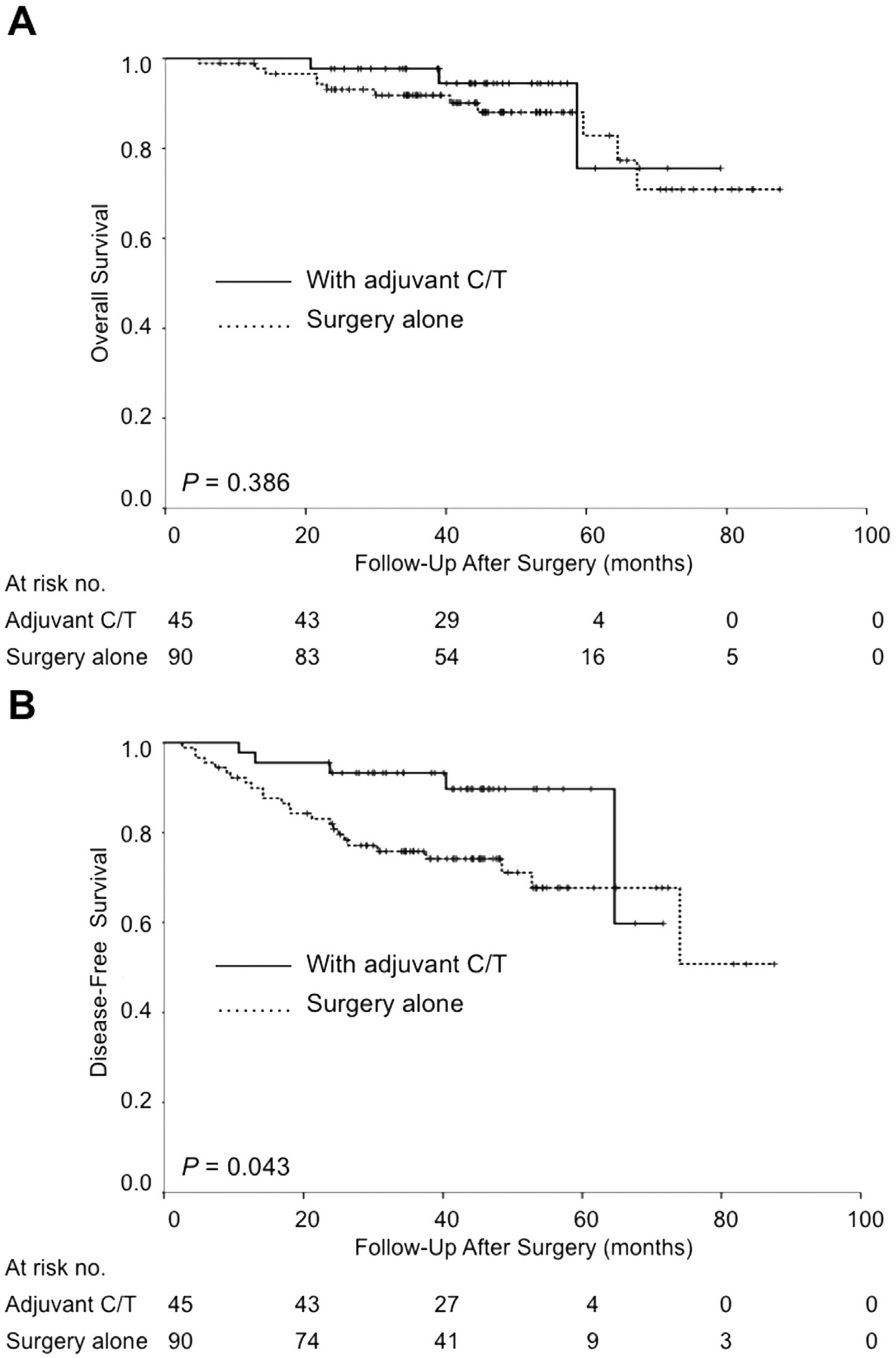


*Fig 1. Kaplan-Meier survival curves for (A) overall survival and (B) probability of freedom from recurrence (FFR) in a propensity-score–matched comparison of patients treated with surgical intervention alone and those who received adjuvant chemotherapy (log-rank test).*

ARTICLE IN PRESS



alone and 26 were treated with adjuvant chemotherapy. The relationship between the use of adjuvant chemotherapy and clinicopathologic variables in these patients is shown in Table 4. There was no significant difference between the use of adjuvant chemotherapy and clinicopathologic variables. Treatment with adjuvant chemotherapy ($p = 0.128$) was not a significant prognostic factor for OS (Appendix Table 1). Treatment with adjuvant chemotherapy ($p = 0.033$) was a significant prognostic factor for a better probability of FFR in multivariate analysis (Figs 2A, 2B).

For patients with an acinar/papillary–predominant pattern, adjuvant therapy was not a significant prognostic factor for OS ($p = 0.908$) and probability of FFR ($p = 0.322$) in univariate analysis (Appendix Table 2; Figs 2C, 2D).

## Comment

This study investigated the benefits of adjuvant chemotherapy in patients with completely resected stage IB lung adenocarcinoma. For patients with a predominantly micropapillary/solid pattern, adjuvant chemotherapy was a significant prognostic factor for better probability of FFR in multivariate analysis.

Although several studies have reported that adjuvant chemotherapy is effective in patients with stage I NSCLC [9, 22, 23], it is still controversial whether adjuvant chemotherapy improves survival in patients with stage IB lung cancer. In the present study, adjuvant chemotherapy was not a significant prognostic factor for OS. However, adjuvant chemotherapy was a significant prognostic factor for better probability of FFR in multivariate analysis. To further accurately assess the impact of adjuvant chemotherapy on outcomes, we performed propensity-score–matched analysis with covariates including age, sex, invasive tumor size, visceral pleural invasion, predominant histologic pattern, $FEV_1$, DLCO, and comorbidity. Although patients with adjuvant chemotherapy showed no significant difference in OS compared with that of patients who had surgical intervention alone among propensity-score–matched pairs, the prognostic value of adjuvant chemotherapy for probability of FFR was significant. Patients who received adjuvant chemotherapy had a significantly better probability of FFR than patients who underwent operation alone.

*Table 4. Relationship Between Adjuvant Chemotherapy (Surgical Intervention Alone or With Adjuvant Chemotherapy) and Clinicopathologic Variables in 63 Patients With a Micropapillary/Solid–Predominant Pattern*

| Variable | Surgical Intervention Alone (n = 37) | With Adjuvant Therapy (n = 26) | *p* Value |
|---|---|---|---|
| Age, y (mean ± SD) | 66.5 ± 11.0 | 61.7 ± 10.1 | 0.086 |
| Sex, n (%) | | | |
| Male | 24 (64.9) | 15 (57.7) | 0.564 |
| Female | 13 (35.1) | 11 (42.3) | |
| Invasive tumor size, n (%) | | | |
| ≤3 cm | 20 (54.1) | 16 (61.5) | 0.555 |
| >3 cm | 17 (45.9) | 10 (38.5) | |
| Visceral pleural invasion, n (%)[a] | | | |
| Absent | 4 (11.1) | 4 (15.4) | 0.620 |
| Present | 32 (88.9) | 22 (84.6) | |
| $FEV_1$ (% predicted) (mean) | 91.7 | 96.3 | 0.257 |
| DLCO (% predicted) (mean) | 70.4 | 72.4 | 0.588 |
| Comorbidity, n (%) | | | |
| No | 19 (51.4) | 16 (61.5) | 0.423 |
| Yes | 18 (48.6) | 10 (38.5) | |

[a] Patients with unknown status were excluded from the analysis.

DLCO = diffusing capacity of the lungs for carbon monoxide; $FEV_1$ = forced expiratory volume in 1 second; SD = standard deviation.

Identification of prognostic factors in stage IB lung adenocarcinoma is helpful to stratify patients with a high risk of death or recurrence for the future design of adjuvant chemotherapy trials. A predominant pattern of lepidic adenocarcinoma has been reported to be less chemosensitive to platinum-based chemotherapy [24]. Warth and colleagues [14] reported that the prognostic impact of the new classification of lung adenocarcinoma was significant when stratified by adjuvant radiotherapy. Solid predominant tumors had an improved prognosis with adjuvant radiotherapy in their report [14]. By analyzing small biopsy specimens of advanced lung adenocarcinoma, Campos-Parra and coworkers [25] have shown that response rate and progression-free survival in platinum-based chemotherapy were better for high-grade (papillary-, micropapillary-, and solid-predominant) adenocarcinoma. However, the sampling bias from a small biopsy specimen may lead to an incomplete classification of the IASLC/ATS/ERS subtype [26]. In a Lung Adjuvant Cisplatin Evaluation (LACE-BIO) study–related report, patients with micropapillary/solid–predominant adenocarcinomas (but not acinar/papillary–predominant adenocarcinomas) attained disease-free survival and specific disease-free survival but not OS benefit from adjuvant chemotherapy [27, 28]. In our previous study [19], the prognostic value of the new classification was significant in chemotherapy-naive patients. We further showed that predominantly solid tumors were also predictive of poor OS in patients receiving adjuvant chemotherapy [19]. The current study showed that patients having stage IB lung adenocarcinoma with a predominantly micropapillary/solid pattern had significantly improved probability of FFR when they received platinum-based adjuvant chemotherapy. No survival or FFR benefit was identified in patients with acinar/papillary–predominant adenocarcinoma after adjuvant chemotherapy. Therefore the micropapillary/solid–predominant pattern may be proposed as a stratification factor in the design of future clinical trials on adjuvant chemotherapy. Our report is the first in the literature to show the predictive value of a micropapillary/solid–predominant pattern for benefits from adjuvant chemotherapy in stage IB lung adenocarcinoma.

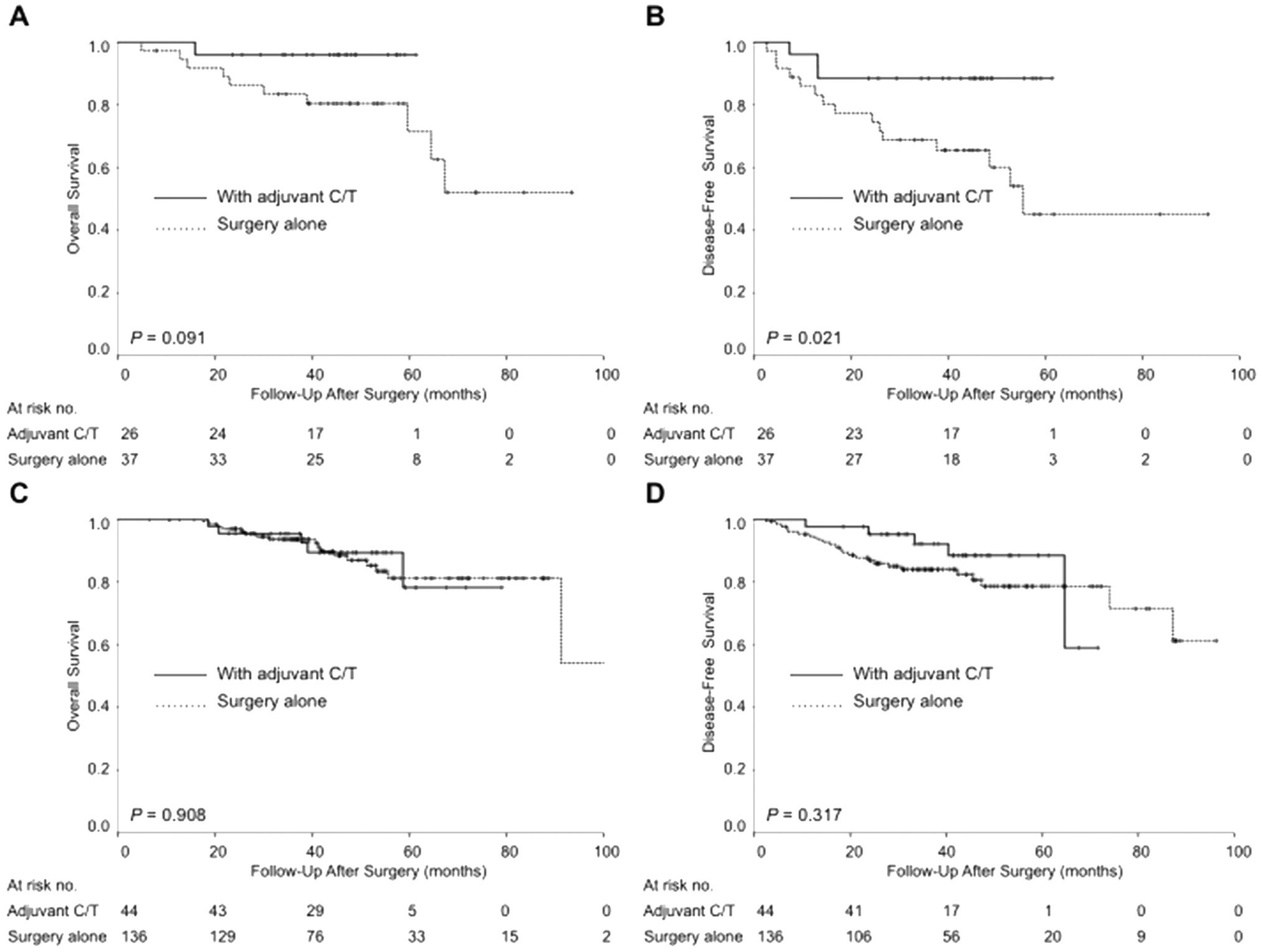


*Fig 2. Kaplan-Meier survival curves for (A and C) overall survival, and (B and D) probability of freedom from recurrence (FFR) according to the use of adjuvant therapy (surgical intervention alone versus with adjuvant chemotherapy) in patients with (A and B) predominantly micropapillary or solid adenocarcinoma and (C and D) predominantly acinar or papillary adenocarcinoma (log-rank test).*

Some limitations of this study should be mentioned. As a retrospective study, patient selection bias and time trend bias are inevitable. Another limitation is that the use of adjuvant chemotherapy and selection of the regimens used for adjuvant therapy were not randomized but were according to physician preference. To evaluate the benefit of adjuvant chemotherapy for early-stage lung cancer, the number of patients was too small. The follow-up of patients was insufficient to evaluate long-term results of adjuvant chemotherapy. Prospective multi-institutional studies and randomized clinical trials are mandatory to validate further the value of the new IASLC/ATS/ERS classification for predicting benefits from adjuvant chemotherapy.

In conclusion, adjuvant chemotherapy is a favorable prognostic factor for probability of FFR in patients with stage IB lung adenocarcinoma, particularly in those with a predominantly micropapillary/solid pattern. This information is important for the further design of clinical randomized trials investigating aggressive adjuvant therapy.

This work was supported in part by the Ministry of Science and Technology (MOST 104-2314-B-075-062 [J-J.H.]), Taipei Veterans General Hospital (V104C-173 [J-J.H.]) Taipei Veterans General Hospital (V104E8-002 [J-J.H. and Y-C.W.]), Taipei Veterans General Hospital-National Yang-Ming University-Excellent Physician Scientists Cultivation Program (104-V-B-005 [J-J.H.]), Yen Tjing-Ling Medical Foundation (CI-104-8 [J-J.H.] and Y-C.W]), Li-Yang Sheen Medical Education Memorial Foundation (J-J.H. and W-H.H.), and MOHW103-TD-B-111-02 (Y-C.W.).

The authors are grateful to Drs Han-Shui Hsu, Chih-Cheng Hsieh, Chien-Sheng Huang, Yu-Chin Lee, Chun-Ming Tsai, and Yuh-Min Chen of Taipei Veterans General Hospital, Taipei, Taiwan for their contributions to this article.

## References

1. Torre LA, Bray F, Siegel RL, et al. Global cancer statistics, 2012. CA Cancer J Clin 2015;65:87–108.
2. Ettinger DS, Wood DE, Akerley W, et al. Non-small cell lung cancer, version 6.2015. J Natl Compr Canc Netw 2015;13:515–24.
3. Goldstraw P, Crowley J, Chansky K, et al. The IASLC Lung Cancer Staging Project: proposals for the revision of the

ARTICLE IN PRESS



TNM stage groupings in the forthcoming (seventh) edition of the TNM Classification of malignant tumours. J Thorac Oncol 2007;2:706–14.
4. Hung JJ, Jeng WJ, Hsu WH, et al. Time trends of overall survival and survival after recurrence in completely resected stage I non-small cell lung cancer. J Thorac Oncol 2012;7:397–405.
5. Nakagawa T, Okumura N, Ohata K, et al. Postrecurrence survival in patients with stage I non-small cell lung cancer. Eur J Cardiothorac Surg 2008;34:499–504.
6. Booth CM, Shepherd FA. Adjuvant chemotherapy for resected nonsmall cell lung cancer. J Thorac Oncol 2006;1:180–7.
7. Booth CM, Shepherd FA, Peng Y, et al. Adoption of adjuvant chemotherapy for non-small-cell lung cancer: a population-based outcomes study. J Clin Oncol 2010;28:3472–8.
8. Arriagada R, Auperin A, Burdett S, et al. Adjuvant chemotherapy, with or without postoperative radiotherapy, in operable non–small–cell lung cancer: Two meta-analyses of individual patient data. Lancet 2010;375:1267–77.
9. Strauss GM, Herndon JE 2nd, Maddaus MA, et al. Adjuvant paclitaxel plus carboplatin compared with observation in stage IB non-small-cell lung cancer: CALGB 9633 with the Cancer and Leukemia Group B, Radiation Therapy Oncology Group, and North Central Cancer Treatment Study Groups. J Clin Oncol 2008;26:5043–51.
10. Travis WD, Brambilla E, Noguchi M, et al. International Association for the Study of Lung Cancer/American Thoracic Society/European Respiratory Society international multidisciplinary classification of lung adenocarcinoma. J Thorac Oncol 2011;6:244–85.
11. Sica G, Yoshizawa A, Sima CS, et al. A grading system of lung adenocarcinomas based on histologic pattern is predictive of disease recurrence in stage I tumors. Am J Surg Pathol 2010;34:1155–62.
12. Yoshizawa A, Motoi N, Riely GJ, et al. Impact of proposed IASLC/ATS/ERS classification of lung adenocarcinoma: prognostic subgroups and implications for further revision of staging based on analysis of 514 stage I cases. Mod Pathol 2011;24:653–64.
13. Russell PA, Wainer Z, Wright GM, et al. Does lung adenocarcinoma subtype predict patient survival? A clinicopathologic study based on the new International Association for the Study of Lung Cancer/American Thoracic Society/European Respiratory Society international multidisciplinary lung adenocarcinoma classification. J Thorac Oncol 2011;6:1496–504.
14. Warth A, Muley T, Meister M, et al. The novel histologic International Association for the Study of Lung Cancer/American Thoracic Society/European Respiratory Society Classification system of lung adenocarcinoma is a stage-independent predictor of survival. J Clin Oncol 2012;30:1438–46.
15. Sterlacci W, Savic S, Schmid T, et al. Tissue-sparing application of the newly proposed IASLC/ATS/ERS classification of adenocarcinoma of the lung shows practical diagnostic and prognostic impact. Am J Clin Pathol 2012;137:946–56.
16. Yoshizawa A, Sumiyoshi S, Sonobe M, et al. Validation of the IASLC/ATS/ERS lung adenocarcinoma classification for prognosis and association with EGFR and KRAS gene mutations: analysis of 440 Japanese patients. J Thorac Oncol 2013;8:52–61.
17. Gu J, Lu C, Guo J, et al. Prognostic significance of the IASLC/ATS/ERS classification in Chinese patients—a single institution retrospective study of 292 lung adenocarcinoma. J Surg Oncol 2013;107:474–80.
18. Hung JJ, Jeng WJ, Chou TY, et al. Prognostic value of the new International Association for the Study of Lung Cancer/American Thoracic Society/European Respiratory Society Lung Adenocarcinoma classification on death and recurrence in completely resected stage I lung adenocarcinoma. Ann Surg 2013;258:1079–86.
19. Hung JJ, Yeh YC, Jeng WJ, et al. Predictive value of the International Association for the Study of Lung Cancer/American Thoracic Society/European Respiratory Society classification of lung adenocarcinoma in tumor recurrence and patient survival. J Clin Oncol 2014;32:2357–64.
20. Girard N, Deshpande C, Lau C, et al. Comprehensive histologic assessment helps to differentiate multiple lung primary nonsmall cell carcinomas from metastases. Am J Surg Pathol 2009;33:1752–64.
21. Kaplan EL, Meier P. Nonparametric estimation for incomplete observations. J Am Stat Assoc 1958;53:457–81.
22. Tsutani Y, Miyata Y, Kushitani K, et al. Propensity score-matched analysis of adjuvant chemotherapy for stage I non-small cell lung cancer. J Thorac Cardiovasc Surg 2014;148:1179–85.
23. Malhotra J, Mhango G, Gomez JE, et al. Adjuvant chemotherapy for elderly patients with stage I non-small-cell lung cancer $\geq$4 cm in size: an SEER-Medicare analysis. Ann Oncol 2015;26:768–73.
24. Miller VA, Hirsch FR, Johnson DH. Systemic therapy of advanced bronchioloalveolar cell carcinoma: challenges and opportunities. J Clin Oncol 2005;23:3288–93.
25. Campos-Parra AD, Avilés A, Contreras-Reyes S, et al. Relevance of the novel IASLC/ATS/ERS classification of lung adenocarcinoma in advanced disease. Eur Respir J 2014;43:1439–47.
26. Riely GJ, Travis WD. Can IASLC/ATS/ERS subtype help predict response to chemotherapy in small biopsies of advanced lung adenocarcinoma? Eur Respir J 2014;43:1240–2.
27. Brambilla EM, Marguet S, Le Teuff G, et al. Prognostic and predictive value of a new IASLC/ATS/ERS lung adenocarcinoma classification in a pooled analysis of four adjuvant chemotherapy trials: a LACE-BIO study. J Thorac Oncol 2013;8(Suppl 2):S201.
28. Tsao MS, Marguet S, Teuff G, et al. Subtype classification of lung adenocarcinoma predicts benefits from adjuvant chemotherapy in patients undergoing complete resection. J Clin Oncol 2015;33:3439–46.
