# Supplementary material for: Efficacy and Benefit of Postoperative Chemotherapy in Micropapillray or Solid Predominant Pattern in Stage IB Lung Adenocarcinoma: A Systematic Review and Meta-Analysis
Source: Front Surg. 2021 Dec 21;8:795921. doi: 10.3389/fsurg.2021.795921 (PMC8724201; doi:10.3389/fsurg.2021.795921)
Supplement: Supplementary file 1 [file Data_Sheet_1.ZIP › ╬─╧╫/88.pdf]

Prognostic significance and adjuvant chemotherapy survival benefits of a solid or micropapillary pattern in patients with resected stage IB lung adenocarcinoma

Fangfei Qian, MD,[a] Wenjia Yang, MD,[a] Rui Wang, PhD,[b] Jianlin Xu, PhD,[a] Shuyuan Wang, MD,[a] Yanwei Zhang, PhD,[a] Bo Jin, PhD,[a] Keke Yu, MD,[c] and Baohui Han, MD, PhD[a]

ABSTRACT

Objective: To evaluate the prognostic significance and beneficiaries of adjuvant chemotherapy (ACT) in various histological patterns of stage IB lung adenocarcinoma according to the 8th tumor-node-metastasis (TNM) classification.

Methods: A total of 1131 patients with pathological stage IB lung adenocarcinoma according to the 8th TNM classification who underwent lobectomy or segmentectomy were enrolled in this study. Based on the proportion of solid/micropapillary components, the patients were classified into 3 groups: solid/micropapillary-negative (SMPN) (n = 719; median survival, 49.7 months; interquartile range [IQR]. 35.1-67.0 months), solid/micropapillary-minor (SMPM; >5% but not predominant) (n = 272; median survival, 38.8 months; IQR, 26.6-51.5 months) and solid/micropapillary-predominant (SMPP; >5% and the most dominant) (n = 140; median survival, 39.6 months; IQR, 26.8-52.5 months). The predictors of disease-specific survival and recurrence-free survival were investigated. To reduce selection bias, propensity score-matching analysis was implemented before survival data were compared.

Results: Our data show significant differences in survival rates based on the proportion of solid/micropapillary patterns. The SMPM group had significantly higher cumulative incidences of lung cancer–specific death (P = .000) and recurrence (P = .000) compared with the SMPN group, so did the SMPP group when compared with SMPM patients (P = .000 for both). Multivariate analysis showed that the SMPM and SMPP patterns were poor prognostic factors for disease-specific survival (hazard ratio [HR], 1.86; 95% confidence interval [CI], 1.12-3.09 and HR, 4.56; 95% CI, 2.69-7.71, respectively) and recurrence-free survival (HR, 1.64; 95% CI, 1.20-2.24 and HR, 2.43; 95% CI, 1.64-3.60, respectively), as were older age, male sex, smoking history, larger tumor size, necrosis, and abnormal pulmonary function. Survival analysis stratified by histological pattern showed that patients with the SMPP pattern who received ACT had obviously lower cumulative incidences of lung cancer–specific death (HR, 0.46; 95% CI, 0.22-0.93; P = .031) and recurrence (HR, 0.48; 95% CI, 0.26-0.88; P = .017).

Conclusions: Solid/micropapillary patterns were associated with poor prognosis, even if they were not predominant. ACT contributed to survival benefits in the SMPP subgroup of patients with stage IB lung adenocarcinoma. (J Thorac Cardiovasc Surg 2017;■:1-9)

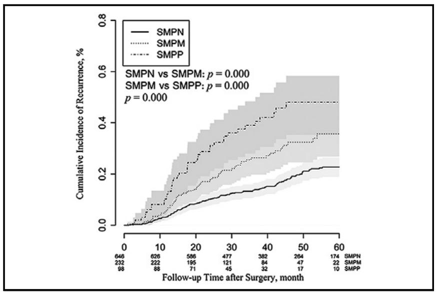

Recurrence-free survival according to the proportion of solid/micropapillary component.

Central Message

In patients with stage IB lung adenocarcinoma, a solid or micropapillary pattern was associated with worse survival. Adjuvant chemotherapy contributed to survival benefits in the predominantly solid or micropapillary pattern subgroup.

Perspective

The study aimed to demonstrate the influence of histological subtype in patients with stage IB lung adenocarcinoma based on the current 8th TNM classification, and to evaluate whether adjuvant chemotherapy had a survival benefit in different patterns. With the increasing number of early-stage lung adenocarcinomas, we hope that our results will have favorable implications for the management of stage IB patients.

See Editorial Commentary page XXX.

THOR

From the Departments of [a]Pulmonary Medicine, [b]Thoracic Surgery, and [c]Pathology, Shanghai Chest Hospital, Shanghai Jiao Tong University, Shanghai, China.

Drs Qian and Yang contributed equally to this work.

This work was supported by the Program for Outstanding Leaders of Shanghai. The funders played no role in the study design, data collection and analysis, decision to publish, or manuscript preparation.

Received for publication March 9, 2017; revisions received Sept 15, 2017; accepted for publication Sept 28, 2017.

Address for reprints: Baohui Han, MD, PhD, West Huaihai Rd No 241, Shanghai 200030, China (E-mail: xkyyhan@gmail.com).

0022-5223/$36.00

Copyright © 2017 by The American Association for Thoracic Surgery

https://doi.org/10.1016/j.jtcvs.2017.09.143

Abbreviations and Acronyms

- ACT = adjuvant chemotherapy
- ATS = American Thoracic Society
- CT = computed tomography
- DSS = disease-specific survival
- ERS = European Respiratory Society
- IASLC = International Association for the Study of Lung Cancer
- NSCLC = non–small cell lung cancer
- OS = overall survival
- PSM = propensity score matching
- RFS = recurrence-free survival
- SMPM = solid/micropapillary-minor
- SMPN = solid/micropapillary-negative
- SMPP = solid/micropapillary-predominant
- TNM = tumor-node-metastasis

Scanning this QR code will take you to a supplemental video and figures for the article.

Non–small cell lung cancer (NSCLC) remains the most common malignant tumor and the major cause of cancer-related death worldwide, with adenocarcinoma the most common histological subtype.[1] Currently, surgical resection is the primary treatment for early-stage NSCLC.[2] Several randomized controlled trials and meta-analyses have shown that adjuvant chemotherapy (ACT) substantially improved survival in patients with resected stage II and IIIA NSCLC[3-8]; however, whether ACT improves both overall survival (OS) and recurrence-free survival (RFS) in patients with stage IB NSCLC remains inconclusive. Stratified analysis of the Adjuvant Navelbine International Trialist Association trial reported no significant improvement in survival in stage IB patients.[5] In the CALGB 9633 trial, ACT was recommended for stage IB patients with tumors >4 cm in diameter.[9] The JBR-10 study showed that ACT offered no survival benefit to stage IB patients, although patients with tumors >4 cm appeared to benefit from chemotherapy.[10] The National Comprehensive Cancer Network guideline suggests that ACT should be considered in stage IB patients with high-risk factors, including tumor size >4 cm, poorly differentiated lymphovascular invasion, visceral pleura invasion, incomplete lymph node sampling, or wedge resection[2]; however, whether these stage IB patients obtain any survival benefits from ACT is unclear, owing to a lack of solid evidence. In addition, previous studies were based on the 6th or 7th tumor-node-metastasis (TNM) classification system, which include different definitions of stage IB than provided in the current 8th TNM staging system,[11-13] making comparison of results from different reports even more difficult.

According to the International Association for the Study of Lung Cancer, American Thoracic Society, and European Respiratory Society (IASLC/ATS/ERS) classification of 2011, invasive adenocarcinoma is categorized into several subtypes based on the predominant pattern, including lepidic, acinar, papillary, solid, and micropapillary, as well as invasive mucinous adenocarcinoma.[14] Different pathological subtypes of adenocarcinomas appear to predict biological behavior. Patients with a predominantly solid/micropapillary pattern have a relatively poor prognosis compared to those with other patterns[15,16]; however, whether the minor component of the solid/micropapillary pattern is related to the unfavorable prognosis remains controversial. Moreover, the prognostic value of the new pathological classification for a benefit from ACT in early stages has not been proven. This study aimed to determine whether in patients with stage IB lung adenocarcinoma according to the 8th TNM classification system, the sold/micropapillary pattern is associated with the poor survival, and whether these patients could benefit from ACT. Given the increasing frequency of early-stage lung adenocarcinomas, we hope that our results will have favorable implications for the management of stage IB patients.

METHODS

Patients

This retrospective study was approved by our hospital's Institutional Review Board. Medical data and tissue specimens were anonymized before the analysis. Between October 2008 and June 2015, patients at our center who underwent lobectomy or segmentectomy for stage IB pulmonary adenocarcinoma based on the 8th TNM classification system were enrolled in this study (Video 1). To exclude distant metastasis, all patients underwent a preoperative routine examination, including enhanced chest computed tomography (CT) scan, enhanced abdominal CT scan or ultrasonography, fiberoptic bronchoscopy, cranial magnetic resonance imaging, and bone scan. A positron emission tomography–CT scan was suggested if possible. Patients who underwent palliative resection with advanced stage, wedge resection, and neo-ACT or who had multiple primary lung cancers were excluded. Finally, a total of 1131 patients with complete histological and follow-up data were included in this study (Figure 1). The patients with high-risk factors, including a predominantly solid or micropapillary pattern, visceral pleura invasion, and lymphovascular invasion, were indicated for platinum-based ACT. Four cycles of chemotherapy were recommended for all patients, unless severe side effects occurred.

Pathological Evaluation

Specimens were handled according to routine clinical practice and the pathology data retrieved from the pathology report. Histopathological analysis of each specimen was performed according to the 2011 IASLC/ATS/ERS classification by 2 experienced lung pathologists. The histological patterns were identified in 5% increments. The predominant histological subtype was determined according to the pattern of

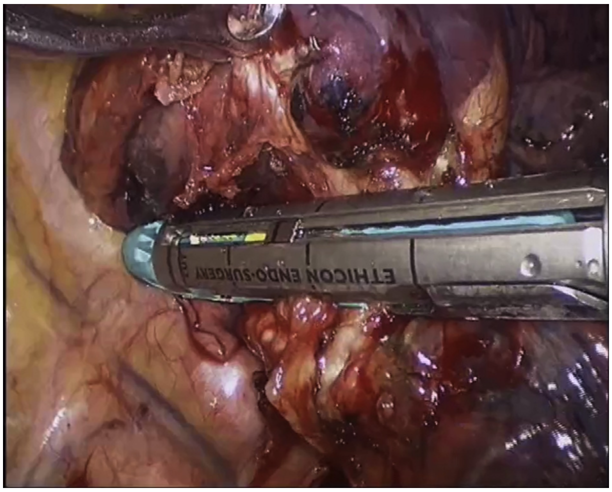

VIDEO 1. Lobectomy and lymph node dissection of right lower lobe of lung. Video available at: http://www.jtcvsonline.org.

highest percentage. All pathologists who were involved in the analysis were blinded to follow-up data. According to the proportion of solid/micropapillary components, patients were classified into 3 groups. Patients without a solid/micropapillary pattern were classified as solid/micropapillary-negative (SMPN), those with a solid/micropapillary component >5% but not a predominant pattern were classified as solid/micropapillary-minor (SMPM), and those with a solid/micropapillary pattern > 5% and of predominant subtype were classified as solid/micropapillary-predominant (SMPP).

Patient Follow-up

All patients were followed at our hospital or other institutions every 3 months for the first 2 years after surgery and every 6 months thereafter. A thoracic CT scan and an abdominal CT scan or ultrasonography were routinely performed at each scheduled visit. Bone scans were performed annually during follow-up. A cranial CT scan or magnetic resonance imaging was performed in the presence of neurologic symptoms. A positron emission tomography–CT scan was suggested if possible.

Statistical Analysis

Categorical and continuous variables were compared between groups using the χ^2 test, Fisher exact test, Mann–Whitney U test, and Kruskal–Wallis test as appropriate. Disease-specific survival (DSS) was defined as the interval from the date of surgery to death due to lung cancer or the last follow-up visit. RFS was defined as the interval from the date of surgery to the date of the first recurrence or death owing to any cause or the last follow-up visit. A competing-risks proportional hazards model was used to analyze DSS, with any death not caused by lung cancer considered a competing event, and RFS, with any death without recurrence considered a competing event. The Fine–Gray proportional hazards model was used to examine predictors. Multivariable analyses were conducted in a forward stepwise manner for factors with $P < .10$ identified in univariate analyses. The proportionality hazards assumptions for each variable was tested and held in the Cox model using a robust variance estimator.

To better correct for selection bias, a propensity score was calculated for all patients, due to the imbalanced data between observation and ACT groups. Patients were matched on baseline, operative, and perioperative variables, including age, sex, smoking history, tumor size, visceral pleural invasion, lymphovascular invasion, bronchial invasion, necrosis, histological patterns, comorbid conditions, pulmonary function, procedure type, tumor location, postprocedure complications, and postprocedure length of stay. We considered a nonparametric generalized boosted modeling technique that uses regression trees for estimating the propensity scores, with a caliper approach as 0.2 of the standard deviation. Nearest-neighbor matching was selected, and done without replacement. Using the propensity scores, patients in the ACT group were randomly matched with those in the observation group with 1:1 matching methods. After propensity score matching (PSM), the prevalence of dichotomous variables was compared between the ACT and observation

THOR

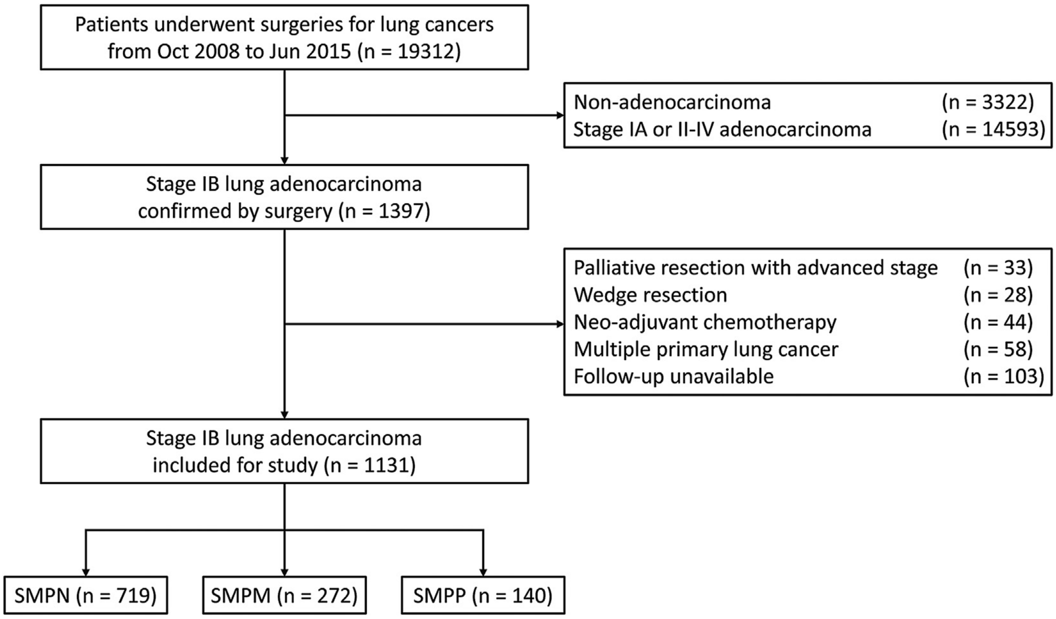

FIGURE 1. CONSORT diagram showing the initial study population and the numbers included and excluded for each of the inclusion/exclusion criteria. *SMPN*, Solid/micropapillary-negative; *SMPM*, solid/micropapillary-minor; *SMPP*, solid/micropapillary-predominant.

TABLE 1. Clinicopathological characteristics of all patients stratified by histological pattern

Variable	SMPN (n = 719)	SMPM (n = 272)	SMPP (n = 140)	P value
Age, y	61 (55-66)	61 (54-66)	61 (56-66)	.793*
Sex				
Male	306 (42.6)	138 (50.7)	100 (71.4)	.000†
Female	413 (57.4)	134 (49.3)	326 (28.6)	
Smoking history				
Yes	209 (29.1)	92 (33.8)	72 (51.4)	.000†
No	510 (70.9)	180 (66.2)	68 (48.6)	
Size	3.0 (2.1-3.5)	2.9 (2.1-3.5)	3.0 (2.5-3.5)	.118*
Visceral pleural invasion				.020†
Yes	473 (65.8)	200 (73.5)	104 (74.3)	
No	246 (34.2)	72 (26.5)	36 (25.7)	
Lymphovascular invasion				
Yes	45 (6.3)	18 (6.6)	14 (10.0)	.272†
No	674 (93.7)	254 (93.4)	126 (90.0)	
Bronchial invasion				
Yes	9 (1.3)	4 (1.5)	5 (3.6)	.146‡
No	710 (98.7)	268 (98.5)	135 (96.4)	
Necrosis				
Yes	10 (1.4)	14 (5.1)	16 (11.4)	.000†
No	709 (98.6)	258 (94.9)	124 (88.6)	
Location 1				
Upper lobe	433 (60.2)	158 (58.1)	79 (56.4)	.639†
Middle/lower lobe	286 (39.8)	114 (41.9)	61 (43.6)	
Location 2				
Peripheral	691 (96.1)	256 (94.1)	136 (97.1)	.262†
Central	28 (3.9)	16 (5.9)	4 (2.9)	
Comorbid conditions				
Yes	248 (34.5)	95 (34.9)	52 (37.1)	.834†
No	471 (65.5)	177 (65.1)	88 (62.9)	
Pulmonary function				
Abnormal	27 (3.8)	15 (5.5)	10 (7.1)	.153†
Normal	692 (96.2)	257 (94.5)	130 (92.9)	
Procedure 1				
Lobectomy	646 (89.8)	250 (91.9)	122 (87.1)	.302†
Segmentectomy	73 (10.2)	22 (8.1)	18 (12.9)	
Procedure 2				
VATS	695 (96.7)	263 (96.7)	136 (97.1)	.957†
Open	24 (3.3)	9 (3.3)	4 (2.9)	
ACT				
Yes	382 (53.1)	150 (55.1)	89 (63.6)	.075†
No	337 (46.9)	122 (44.9)	51 (36.4)	
Postprocedure complications				
Yes	32 (4.5)	9 (3.3)	8 (5.7)	.507†
No	687 (95.5)	263 (96.7)	132 (94.3)	
Postprocedure length of stay, d				
≤7	676 (94.0)	257 (94.5)	132 (94.3)	.960†
>7	43 (6.0)	15 (5.5)	8 (5.7)	
Recurrence				
Recurrence	136 (18.9)	70 (25.7)	53 (37.9)	.000‡
Other-cause mortality	9 (1.3)	1 (0.4)	4 (2.9)	
No recurrence	574 (79.8)	201 (73.9)	83 (59.3)	
Mortality				
Lung cancer mortality	52 (7.2)	25 (9.2)	34 (24.3)	.000‡
Other-cause mortality	11 (1.5)	1 (0.4)	4 (2.9)	
No mortality	656 (91.2)	246 (90.4)	102 (72.9)	

SMPN, Solid/micropapillary-negative; *SMPM*, solid/micropapillary-minor; *SMPP*, solid/micropapillary-predominant; *VATS*, video-assisted thoracic surgery; *ACT*, adjuvant chemotherapy. *Kruskal–Wallis test. †χ^2 test. ‡Fisher exact test.

groups using McNemar's test, whereas a standard 2-sample *t* test was used to compare continuous covariates. Statistical analyses were performed using SPSS version 22.0 (IBM, Armonk, NY) and R version 3.4.1 (R Development Core Team, Vienna, Austria), with the significance level set at $P < .05$.

RESULTS

Clinicopathological Characteristics

The median follow-up time for all 1131 patients was 46.8 months, ranging from 0.3 to 85.8 months. The clinical characteristics of all patients stratified by histological pattern are summarized in Table 1. The 1131 patients included 719 (63.6%) in the SMPN group, 272 (24.0%) in the SMPM group, and 140 (12.4%) in the SMPP group. The 3 groups showed significant differences in terms of sex ($P = .000$), smoking history ($P = .000$), visceral pleural invasion ($P = .020$), necrosis ($P = .000$), recurrence ($P = .000$), and lung cancer–specific death ($P = .000$). In brief, the SMPN subgroup included more females and nonsmokers and had less visceral pleural invasion and necrosis, whereas the SMPP subgroup included more males and smokers, and had more visceral pleural invasion and necrosis. Patients in the SMPP subgroup required ACT more often (63.6%) compared with the other subgroups (~50%), although the difference across the 3 groups was not statistically significant ($P = .075$). The survival outcomes of each subgroup are summarized in Table 1. There were significant differences in the rates of recurrence and lung cancer mortality among the subgroups ($P = .000$ for both).

PSM Results

Among patients with stage IB lung adenocarcinoma, compared with the patients treated with observation, those treated with ACT were older ($P = .000$), had larger tumors ($P = .000$), and had higher rates of necrosis ($P = .003$[illegible] SMPP pattern ($P = .075$), and open surgery ($P = .000$[illegible] There were no significant between-group differences i[illegible]

ARTICLE IN PRESS

TABLE 2. Clinicopathological characteristics of patients before and after PSM

Characteristic	Before PSM Observation (n = 510)	Before PSM ACT (n = 621)	Before PSM SD	Before PSM *P* value	After PSM Observation (n = 488)	After PSM ACT (n = 488)	After PSM SD	After PSM *P* value
Age, y	62 (56-68)	60 (54-65)	0.27	.000*	60.76 ± 8.61	60.06 ± 7.72	0.08	.175‡
Sex								
Male	240 (47.1)	304 (49.0)	0.04	.526†	229 (46.9)	231 (47.3)	0.01	.949§
Female	270 (52.9)	317 (51.0)			259 (53.1)	257 (52.7)		
Smoke								
Yes	158 (31.0)	215 (34.6)	0.08	.195†	151 (30.9)	154 (31.6)	0.00	.889§
No	352 (69.0)	406 (65.4)			337 (69.1)	334 (68.4)		
Size	3.0 (2.0-3.5)	3.0 (2.2-3.5)	−0.01	.000*	2.80 ± 0.79	2.79 ± 0.79	0.02	.734‡
Visceral pleural invasion								
Yes	341 (66.9)	436 (70.2)	0.07	.227†	331 (67.8)	352 (72.1)	0.09	.170§
No	169 (33.1)	185 (29.8)			157 (32.2)	136 (27.9)		
Lymphovascular invasion								
Yes	28 (5.5)	49 (7.9)	0.10	.111†	27 (5.5)	30 (6.1)	0.03	.791§
No	482 (94.5)	572 (92.1)			461 (94.5)	458 (93.9)		
Bronchial invasion								
Yes	5 (1.0)	13 (2.1)	0.09	.137†	5 (1.0)	5 (1.0)	0.00	1.000§
No	505 (99.0)	608 (97.9)			483 (99.0)	483 (99.0)		
Necrosis								
Yes	9 (1.8)	31 (5.0)	0.18	.003†	9 (1.8)	5 (1.0)	0.07	.424§
No	501 (98.2)	590 (95.0)			479 (98.2)	483 (99.0)		
Pattern								
SMPN	337 (66.1)	382 (61.5)	0.14	.075†	320 (65.6)	326 (66.8)	0.03	.755§
SMPM	122 (23.9)	150 (24.2)			118 (24.2)	114 (23.4)		
SMPP	51 (10.0)	89 (14.3)			50 (10.2)	48 (9.8)		
Location 1								
Upper lobe	297 (58.2)	373 (60.1)	0.04	.533†	287 (58.8)	296 (60.7)	0.04	.587§
Middle/lower lobe	213 (41.8)	248 (39.9)			201 (41.2)	192 (39.3)		
Location 2								
Peripheral	491 (96.3)	592 (95.3)	0.05	.433†	470 (96.3)	472 (96.7)	0.02	.864§
Central	19 (3.7)	29 (4.7)			18 (3.7)	16 (3.3)		
Comorbid conditions								
Yes	185 (36.3)	210 (33.8)	0.05	.388†	173 (35.5)	164 (33.6)	0.04	.597§
No	325 (63.7)	411 (66.2)			315 (64.5)	324 (66.4)		
Pulmonary function								
Abnormal	29 (5.7)	23 (3.7)	0.09	.113†	25 (5.1)	20 (4.1)	0.05	.542§
Normal	481 (94.3)	598 (96.3)			463 (94.9)	468 (95.9)		
Procedure 1								
Lobectomy	452 (88.6)	566 (91.1)	0.08	.160†	432 (88.5)	440 (90.2)	0.05	.434§
Segmentectomy	58 (11.4)	55 (8.9)			56 (11.5)	48 (9.8)		
Procedure 2								
VATS	500 (98.0)	594 (95.7)	0.13	.025†	479 (98.2)	480 (98.4)	0.02	1.000§
Open	10 (2.0)	27 (4.3)			9 (1.8)	8 (1.6)		
Postprocedure complications								
Yes	21 (4.1)	28 (4.5)	0.02	.748†	19 (3.9)	15 (3.1)	0.04	.597§
No	489 (95.9)	593 (95.5)			469 (96.1)	473 (96.9)		
Length of stay, d								
≤7	484 (94.9)	593 (93.6)	0.06	.338†	465 (95.3)	466 (95.5)	0.01	1.000§
>7	26 (5.1)	40 (6.4)			23 (4.7)	22 (4.5)		

Continuous variables are reported as median (interquartile range) or mean ± standard deviation. Dichotomous variables are reported as number (%). *PSM*, Propensity score matching; *ACT*, adjuvant chemotherapy; *SD*, standard deviation; *SMPN*, solid/micropapillary-negative; *SMPM*, solid/micropapillary-minor; *SMPP*, solid/micropapillary-predominant; *VATS*, video-assisted thoracic surgery. *Mann-Whitney *U* test. †χ^2 test. ‡Paired-samples *t* test. §McNemar test.

THOR

TABLE 3. Univariable and multivariable analyses of disease-specific survival and recurrence-free survival in patients after PSM

Variable	Univariate analysis			Multivariate analysis		
	HR	95% CI	*P* value	HR	95% CI	*P* value
Disease-specific survival						
Age	1.04	1.02-1.07	.001	1.03	1.00-1.06	.000
Male sex	2.60	1.71-3.96	.000	1.42	0.76-2.66	.000
Positive smoking history	2.59	1.75-3.83	.000	1.51	0.82-2.81	.000
Visceral pleural invasion	1.18	0.75-1.85	.467			
Lymphovascular invasion	1.22	0.60-2.48	.576			
Bronchial invasion	1.10	0.17-7.09	.918			
SMPM pattern	1.99	1.20-3.29	.000	1.86	1.12-3.09	.000
SMPP pattern	6.51	4.11-10.32	.000	4.56	2.69-7.71	.000
Size	1.44	1.11-1.88	.007	1.23	0.94-1.62	.000
Necrosis	7.39	3.55-15.40	.000	1.92	0.76-4.87	.000
Left lobe	1.06	0.71-1.57	.784			
Central location	1.18	0.44-3.15	.745			
Comorbid conditions	1.33	0.89-1.98	.160			
Abnormal pulmonary function	2.58	1.30-5.14	.007	1.07	0.50-2.32	.000
Segmentectomy	1.49	0.83-2.68	.187			
Open surgery	1.30	0.31-5.44	.723			
Adjuvant chemotherapy	0.79	0.54-1.17	.238			
Postprocedure complications	1.54	0.63-3.75	.343			
Postprocedure length of stay	1.42	0.62-3.27	.407			
Recurrence-free survival						
Age	1.02	1.00-1.03	.046	1.01	0.99-1.02	.000
Male sex	1.60	1.23-2.09	.000	1.24	0.84-1.84	.000
Positive smoking history	1.55	1.19-2.02	.001	1.07	0.72-1.61	.000
Visceral pleural invasion	1.34	0.99-1.81	.057	1.62	1.14-2.30	.000
Lymphovascular invasion	1.12	0.68-1.86	.649			
Bronchial invasion	1.41	0.49-4.04	.523			
SMPM pattern	1.80	1.32-2.44	.000	1.64	1.20-2.24	.000
SMPP pattern	3.09	2.16-4.42	.000	2.43	1.64-3.60	.000
Size	1.18	1.00-1.40	.049	1.30	1.06-1.59	.000
Necrosis	2.78	1.33-5.81	.007	1.49	0.69-3.19	.000
Left lobe	1.13	0.87-1.47	.379			
Central location	1.17	0.61-2.24	.638			
Comorbid conditions	1.14	0.87-1.49	.347			
Abnormal pulmonary function	2.25	1.39-3.65	.001	1.51	0.89-2.58	.000
Segmentectomy	1.03	0.66-1.61	.905			
Open surgery	1.38	0.58-3.28	.465			
Adjuvant chemotherapy	1.00	0.77-1.30	.997			
Postprocedure complications	1.17	0.61-2.25	.642			
Postprocedure length of stay	1.20	0.67-2.15	.537			

HR, Hazard ratio; *CI*, confidence interval; *SMPM*, solid/micropapillary-minor; *SMPP*, solid/micropapillary-predominant.

the distribution of other baseline characteristics. To evaluate the quality of matching for each confounding variable, we calculated the standardized differences between the observation and ACT groups before and after matching. PSM effectively reduced the heterogeneity among pretreatment variables between the 2 groups, as shown by the standardized difference plots in Figure E1. Details of each confounding variable before and after matching are summarized in Table 2. PSM between the patients who received ACT (n = 621) and those who underwent observation (n = 510) resulted in 488 matched pairs. Mirror histograms for the propensity scores showing the region of common support between the groups are provided in Figure E2.

Survival Analysis

Results of univariate and multivariate competing-risk analysis for all propensity score–matched patients are summarized in Table 3. The prognostic variables identified in the univariate analysis of DSS, including age, sex, smoking history, histological pattern, tumor size, necrosis, and pulmonary function, were incorporated into the

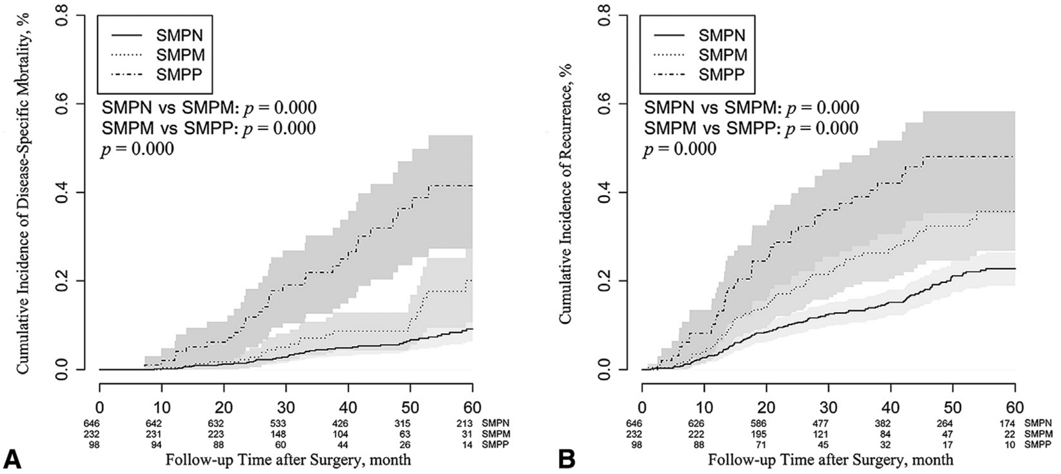

FIGURE 2. Cumulative incidence of disease-specific death (A) and recurrence (B) for different histological patterns. *SMPN*, Solid/micropapillary-negative; *SMPM*, solid/micropapillary-minor; *SMPP*, solid/micropapillary-predominant.

multivariate analysis, and all remained significant. Among the histological patterns, SMPM (subdistribution hazard ratio [SHR], 1.86; 95% confidence interval [CI], 1.12-3.09; $P = .00$) was significantly correlated with shorter DSS, and SMPP (SHR, 4.56; 95% CI, 2.69-7.71; $P = .00$) was associated with even shorter DSS than the other 2 patterns (Figure 2, *A*). For RFS, the same factors were identified for the multivariate analysis, and all were significant. The SMPM group (SHR, 1.64; 95% CI, 1.20-2.24; $P = .00$) had a shorter RFS than the SMPN group, and the SMPP group (SHR, 2.43; 95% CI, 1.64-3.60; $P = .00$) had the worst RFS of the 3 groups (Figure 2, *B*). Figure 3, shows the cumulative incidences of lung cancer–specific death and recurrence in patients with different histological patterns. ACT did not contribute to a survival benefit for all of the propensity score–matched patients; however, survival analysis stratified by histological pattern showed that patients in the SMPP group who received ACT had obviously lower cumulative incidences of lung cancer–specific death (HR, 0.46; 95% CI, 0.22-0.93; $P = .031$) (Figure 3, *C*) and recurrence (HR, 0.48; 95% CI, 0.26-0.88; $P = .017$) (Figure 4, *C*).

DISCUSSION

Although several previous studies have reported that ACT is beneficial for resected stage IB NSCLC, Roselli and colleagues[17] suggested that ACT may have an influence on stage IB patients by reducing the risk of recurrence and metastasis. The CALGB trial confirmed that ACT significantly reduced mortality in patients with stage IB NSCLC.[9] In an analysis of the Surveillance, Epidemiology and End Results–Medicare linked database, Malhotra and colleagues[18] found that ACT improved OS in patients age >65 years with resected T2N0 NSCLC ≥4 cm. Whether ACT improves the survival of patients with stage IB pulmonary adenocarcinoma remains controversial. These previous studies were based mainly on the 6th or 7th TNM classification system, in which the definition of stage

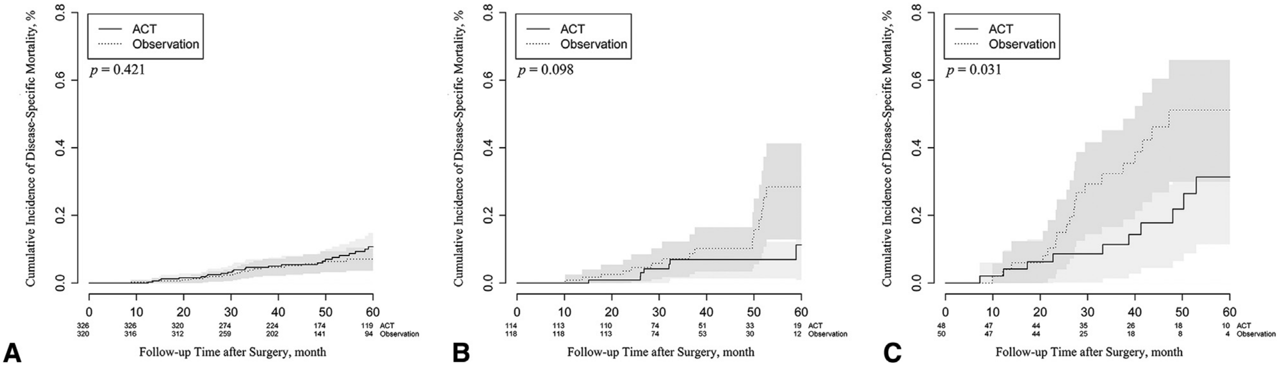

FIGURE 3. Cumulative incidence of disease-specific death of propensity score-matched solid/micropapillary-negative (SMPN) (A), solid/micropapillary-minor (SMPM) (B), and solid/micropapillary-predominant (SMPP) (C) subgroups compared with patients with observation and adjuvant chemotherapy after surgery. *ACT*, Adjuvant chemotherapy.

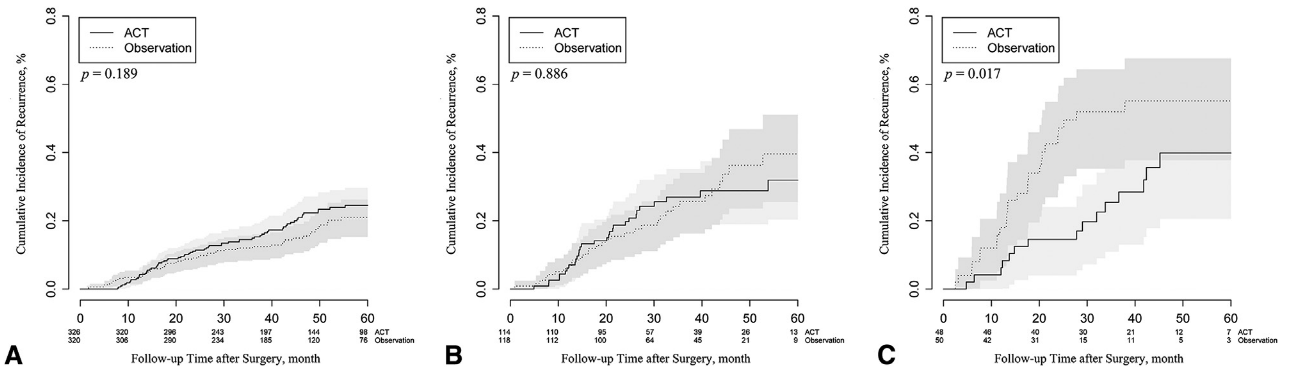

FIGURE 4. Cumulative incidence of recurrence of propensity score-matched solid/micropapillary-negative (SMPN) (A), solid/micropapillary-minor (SMPM) (B), and solid/micropapillary-predominant (SMPP) (C) subgroups compared with patients with observation and adjuvant chemotherapy after surgery. *ACT*, Adjuvant chemotherapy.

IB differs from the current definition. According to the 8th TNM classification, stage IB is defined as a tumor >3 cm but ≤4 cm in greatest dimension without lymph node involvement or distant metastasis.[13] To our knowledge, this study is the first large cohort study to evaluate the influence of ACT on stage IB patients based on the newest staging system.

The 2011 IASLC/ATS/ERS classification divided invasive lung adenocarcinoma into distinct histological subtypes, which significantly contribute to predicting prognosis. It has been shown that patients with an SMPP pattern have an unfavorable survival rate.[19-21] Moreover, Solis and colleagues[22] identified the presence of a solid component as a marker of poor prognosis. It also has been reported that the presence of micropapillary histological pattern of ≥5% or more increases the risk of recurrence.[23] Yanagawa and colleagues[24] found a poorer prognosis in patients with a solid or micropapillary pattern, even if the pattern is not predominant. Our findings indicate the high malignant potential of solid and micropapillary patterns even in early-stage adenocarcinoma. Moreover, the prognosis is strongly associated with the proportion of solid and micropapillary patterns.

Nevertheless, the prognostic value of the newest histological classification for selecting patients who will benefit from ACT remains questionable. Campos-Parra and colleagues[25] reported better OS in patients with advanced adenocarcinoma with an SMPP pattern due to a better response to chemotherapy. Tsao and colleagues[26] demonstrated that in patients with early-stage lung adenocarcinoma, the SMPP pattern was a predictive marker of survival benefit from ACT. They also identified ACT as a significant factor in the prognosis of RFS in stage IB patients with an SMPP pattern. However, other studies have shown that patients who received ACT still had poor survival when tumor size was >4 cm, which may have resulted in an insignificant OS benefit in patients with an SMPP pattern.[27,28] Kadota and colleagues[29] reported that tumor spread through air spaces was more frequently identified in patients with a solid/micropapillary pattern. This may explain why the 2 patterns were associated with a high risk of recurrence and why the patients with an SMPP pattern could benefit from ACT. Our results are in accordance with these studies, showing that ACT notably improved OS, DSS, and RFS in stage IB patients with an SMPP pattern. The patients with an SMPM pattern showed a non-significant tendency toward a survival benefit from ACT, and thus further long-term follow-up and randomized clinical trials are warranted.

This retrospective study has several limitations that should be taken into consideration. First, patient selection bias and the choice of ACT were not randomized, but instead reflected physician preference. Thus, we performed PSM to eliminate the selection bias as much as possible, although there are unrecognized or unmeasured confounders in the matching, such as the patient's economic status and disposition from the hospital. We included as many confounders as possible that might influence both the decision for ACT and the survival outcomes. Second, the size of the subgroup with an SMPP pattern was relatively small. Third, the underlying mechanism responsible for the benefit of ACT remains unknown. Although all patients who received ACT in this study had completed 4 cycles of treatment, the regimens, dosages, and toxicity were not been evaluated. Last but not least, the postrecurrence treatments were not analyzed, which might have influenced OS. Multi-institutional studies and randomized trials are needed to validate our results.

CONCLUSIONS

The proportion of solid or micropapillary patterns is a significant marker of poor prognosis in patients with stage IB lung adenocarcinoma as defined by the 8th TNM classification. In these patients, ACT is considered a favorable prognostic factor for survival benefits in the subgroup with a predominantly solid or micropapillary

pattern. This may aid the selection of patients who will benefit from ACT and contribute to the management of patients with stage IB lung adenocarcinoma.

Conflict of Interest Statement

Authors have nothing to disclose with regard to commercial support.

References

1. Torre LA, Bray F, Siegel RL, Ferlay J, Lortet-Tieulent J, Jemal A. Global cancer statistics, 2012. *CA Cancer J Clin*. 2015;65:87-108.
2. Ettinger DS, Wood DE, Akerley W, Bazhenova LA, Borghaei H, Camidge DR, et al. Non-small cell lung cancer, version 6.2015. *J Natl Compr Canc Netw*. 2015;13:515-24.
3. Arriagada R, Bergman B, Dunant A, Le Chevalier T, Pignon JP, Vansteenkiste J. Cisplatin-based adjuvant chemotherapy in patients with completely resected non-small-cell lung cancer. *N Engl J Med*. 2004;350:351-60.
4. Winton T, Livingston R, Johnson D, Rigas J, Johnston M, Butts C, et al. Vinorelbine plus cisplatin vs observation in resected non-small-cell lung cancer. *N Engl J Med*. 2005;352:2589-97.
5. Douillard JY, Rosell R, De Lena M, Carpagnano F, Ramlau R, Gonzáles-Larriba JL, et al. Adjuvant vinorelbine plus cisplatin versus observation in patients with completely resected stage IB–IIIA non-small-cell lung cancer (Adjuvant Navelbine International Trialist Association [ANITA]): a randomised controlled trial. *Lancet Oncol*. 2006;7:719-27.
6. Pignon JP, Tribodet H, Scagliotti GV, Douillard JY, Shepherd FA, Stephens RJ, et al. Lung adjuvant cisplatin evaluation: a pooled analysis by the LACE Collaborative Group. *J Clin Oncol*. 2008;26:3552-9.
7. Non-Small Cell Lung Cancer Collaborative Group. Chemotherapy in non-small cell lung cancer: a meta-analysis using updated data on individual patients from 52 randomised clinical trials. *BMJ*. 1995;311:899-909.
8. NSCLC Meta-Analyses Collaborative Group, Arriagada R, Auperin A, Burdett S, Higgins JP, Johnson DH, Le Chevalier T, et al. Adjuvant chemotherapy, with or without postoperative radiotherapy, in operable non-small-cell lung cancer: two meta-analyses of individual patient data. *Lancet*. 2010;375:1267-77.
9. Strauss GM, Herndon JE II, Maddaus MA, Johnstone DW, Johnson EA, Harpole DH, et al. Adjuvant paclitaxel plus carboplatin compared with observation in stage IB non-small-cell lung cancer: CALGB 9633 with the Cancer and Leukemia Group B, Radiation Therapy Oncology Group, and North Central Cancer Treatment Group Study Groups. *J Clin Oncol*. 2008;26:5043-51.
10. Butts CA, Ding K, Seymour L, Twumasi-Ankrah P, Graham B, Gandara D, et al. Randomized phase III trial of vinorelbine plus cisplatin compared with observation in completely resected stage IB and II non-small-cell lung cancer: updated survival analysis of JBR-10. *J Clin Oncol*. 2010;28:29-34.
11. Mountain CF. Revisions in the International System for Staging Lung Cancer. *Chest*. 1997;111:1710-7.
12. Travis WD, Giroux DJ, Chansky K, Crowley J, Asamura H, Brambilla E, et al. The IASLC Lung Cancer Staging Project: proposals for the inclusion of broncho-pulmonary carcinoid tumors in the forthcoming (seventh) edition of the TNM Classification for Lung Cancer. *J Thorac Oncol*. 2008;3:1213-23.
13. Goldstraw P, Chansky K, Crowley J, Rami-Porta R, Asamura H, Eberhardt WE, et al. The IASLC Lung Cancer Staging Project: proposals for revision of the TNM stage groupings in the forthcoming (eighth) edition of the TNM Classification for Lung Cancer. *J Thorac Oncol*. 2016;11:39-51.
14. Travis WD, Brambilla E, Noguchi M, Nicholson AG, Geisinger KR, Yatabe Y, et al. International Association for the Study of Lung Cancer/American Thoracic Society/European Respiratory Society International Multidisciplinary Classification of Lung Adenocarcinoma. *J Thorac Oncol*. 2011;6:244-85.
15. Yoshizawa A, Motoi N, Riely GJ, Sima CS, Gerald WL, Kris MG, et al. Impact of proposed IASLC/ATS/ERS classification of lung adenocarcinoma: prognostic subgroups and implications for further revision of staging based on analysis of 514 stage I cases. *Mod Pathol*. 2011;24:653-64.
16. Warth A, Muley T, Meister M, Stenzinger A, Thomas M, Schirmacher P, et al. The novel histologic International Association for the Study of Lung Cancer/American Thoracic Society/European Respiratory Society classification system of lung adenocarcinoma is a stage-independent predictor of survival. *J Clin Oncol*. 2012;30:1438-46.
17. Roselli M, Mariotti S, Ferroni P, Laudisi A, Mineo D, Pompeo E, et al. Postsurgical chemotherapy in stage IB nonsmall cell lung cancer: long-term survival in a randomized study. *Int J Cancer*. 2006;119:955-60.
18. Malhotra J, Mhango G, Gomez JE, Smith C, Galsky MD, Strauss GM, et al. Adjuvant chemotherapy for elderly patients with stage I non-small-cell lung cancer ≥4 cm in size: a SEER-Medicare analysis. *Ann Oncol*. 2015;26:768-73.
19. Russell PA, Wainer Z, Wright GM, Daniels M, Conron M, Williams RA. Does lung adenocarcinoma subtype predict patient survival? a clinicopathologic study based on the New International Association for the Study of Lung Cancer/American Thoracic Society/European Respiratory Society international multidisciplinary lung adenocarcinoma classification. *J Thorac Oncol*. 2011;6:1496-504.
20. Hung JJ, Yeh YC, Jeng WJ, Wu KJ, Huang BS, Wu YC, et al. Predictive value of the International Association for the Study of Lung Cancer/American Thoracic Society/European Respiratory Society classification of lung adenocarcinoma in tumor recurrence and patient survival. *J Clin Oncol*. 2014;32:2357-64.
21. Ujiie H, Kadota K, Chaft JE, Buitrago D, Sima CS, Lee MC, et al. Solid predominant histologic subtype in resected stage I lung adenocarcinoma is an independent predictor of early, extrathoracic, multisite recurrence and of poor postrecurrence survival. *J Clin Oncol*. 2015;33:2877-84.
22. Solis LM, Behrens C, Raso MG, Lin HY, Kadara H, Yuan P, et al. Histologic patterns and molecular characteristics of lung adenocarcinoma associated with clinical outcome. *Cancer*. 2012;118:2889-99.
23. Nitadori J, Bograd AJ, Kadota K, Sima CS, Rizk NP, Morales EA, et al. Impact of micropapillary histologic subtype in selecting limited resection vs lobectomy for lung adenocarcinoma of 2cm or smaller. *J Natl Cancer Inst*. 2013;105:1212-20.
24. Yanagawa N, Shiono S, Abiko M, Katahira M, Osakabe M, Ogata SY. The clinical impact of solid and micropapillary patterns in resected lung adenocarcinoma. *J Thorac Oncol*. 2016;11:1976-83.
25. Campos-Parra AD, Avilés A, Contreras-Reyes S, Rojas-Marín CE, Sánchez-Reyes R, Borbolla-Escoboza RJ, et al. Relevance of the novel IASLC/ATS/ERS classification of lung adenocarcinoma in advanced disease. *Eur Respir J*. 2014;43:1439-47.
26. Tsao MS, Marguet S, Le Teuff G, Lantuejoul S, Shepherd FA, Seymour L, et al. Subtype classification of lung adenocarcinoma predicts benefit from adjuvant chemotherapy in patients undergoing complete resection. *J Clin Oncol*. 2015;33:3439-46.
27. Luo J, Huang Q, Wang R, Han B, Zhang J, Zhao H, et al. Prognostic and predictive value of the novel classification of lung adenocarcinoma in patients with stage IB. *J Cancer Res Clin Oncol*. 2016;142:2031-40.
28. Hung JJ, Wu YC, Chou TY, Jeng WJ, Yeh YC, Hsu WH. Adjuvant chemotherapy improves the probability of freedom from recurrence in patients with resected stage IB lung adenocarcinoma. *Ann Thorac Surg*. 2016;101:1346-53.
29. Kadota K, Nitadori J, Sima CS, Ujiie H, Rizk NP, Jones DR, et al. Tumor spread through air spaces is an important pattern of invasion and impacts the frequency and location of recurrences after limited resection for small stage I lung adenocarcinomas. *J Thorac Oncol*. 2015;10:806-14.

Key Words: lung adenocarcinoma, early stage, adjuvant chemotherapy, histological subtype

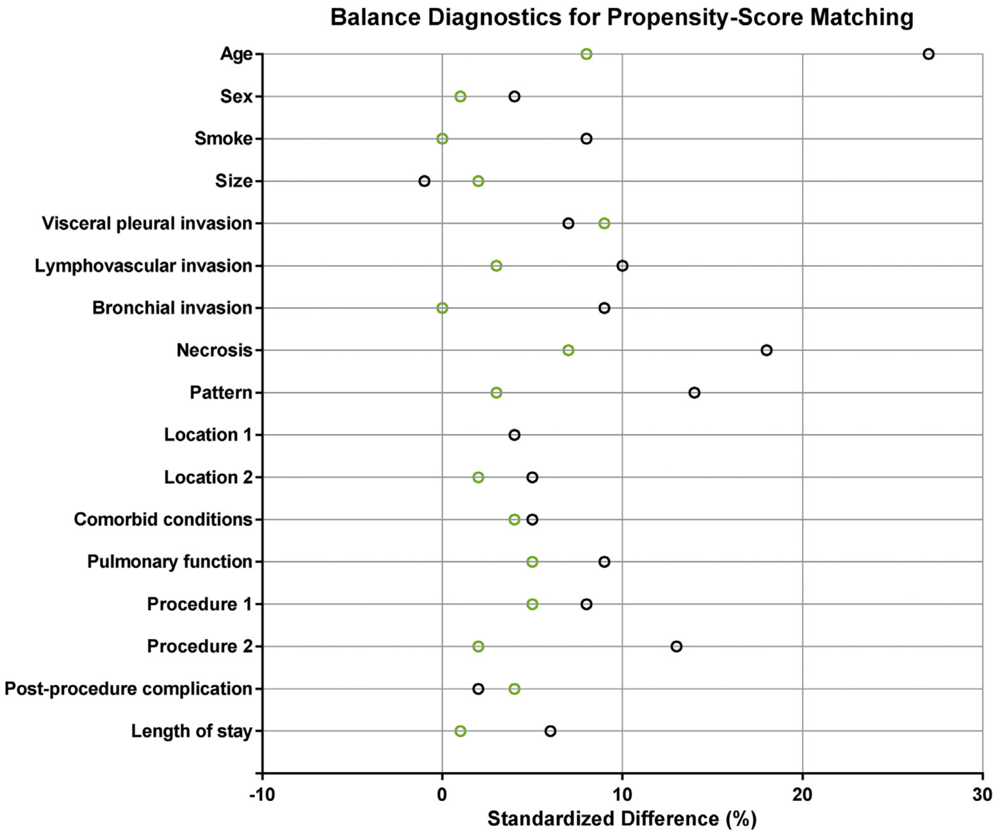

FIGURE E1. The standardized differences plots between patients who received observation and those who underwent adjuvant chemotherapy after surgery. *Black open circles* symbolize difference before propensity score matching (PSM), and *green open circles* symbolize difference after PSM.

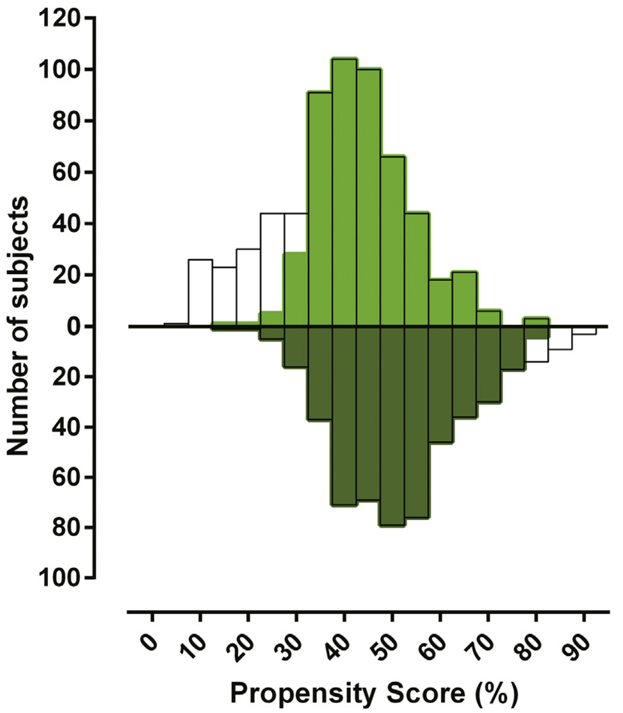

FIGURE E2. Mirror histograms of the propensity scores for patients who underwent observation (below the horizontal line at 0) and those who received adjuvant chemotherapy (above the horizontal line at 0). Matched patients are a subset of the original study population, and their volumes are highlighted in color.

000 Prognostic significance and adjuvant chemotherapy survival benefits of a solid or micropapillary pattern in patients with resected stage IB lung adenocarcinoma

Fangfei Qian, MD, Wenjia Yang, MD, Rui Wang, PhD, Jianlin Xu, PhD, Shuyuan Wang, MD, Yanwei Zhang, PhD, Bo Jin, PhD, Keke Yu, MD, and Baohui Han, MD, PhD, Shanghai, China

In patients with stage IB lung adenocarcinoma, a solid or micropapillary pattern was associated with worse survival. Adjuvant chemotherapy contributed to survival benefits in the predominantly solid or micropapillary pattern subgroup.
